# Non-dispersive phloem-protein bodies (NPBs) of *Populus trichocarpa* consist of a SEOR protein and do not respond to cell wounding and Ca^2+^

**DOI:** 10.7717/peerj.4665

**Published:** 2018-04-17

**Authors:** Daniel L. Mullendore, Timothy Ross-Elliott, Yan Liu, Hanjo H. Hellmann, Eric H. Roalson, Winfried S. Peters, Michael Knoblauch

**Affiliations:** 1 School of Biological Sciences, Washington State University, Pullman, WA, USA; 2 Biology Department, University of North Carolina at Chapel Hill, Chapel Hill, NC, USA

**Keywords:** *Populus trichocarpa*, P-protein, Sieve element, Non-dispersive P-protein body, Phloem transport, SEOR protein

## Abstract

Differentiating sieve elements in the phloem of angiosperms produce abundant phloem-specific proteins before their protein synthesis machinery is degraded. These P-proteins initially form dense bodies, which disperse into individual filaments when the sieve element matures. In some cases, however, the dense protein agglomerations remain intact and are visible in functional sieve tubes as non-dispersive P-protein bodies, or NPBs. Species exhibiting NPBs are distributed across the entire angiosperm clade. We found that NPBs in the model tree, *Populus trichocarpa*, resemble the protein bodies described from other species of the order Malpighiales as they all consist of coaligned tubular fibrils bundled in hexagonal symmetry. NPBs of all Malpighiales tested proved unresponsive to sieve tube wounding and Ca^2+^. The *P. trichocarpa* NPBs consisted of a protein encoded by a gene that in the genome database of this species had been annotated as a homolog of *SEOR1* (sieve element occlusion-related 1) in *Arabidopsis*. Sequencing of the gene in our plants corroborated this interpretation, and we named the gene *PtSEOR1*. Previously characterized SEOR proteins form irregular masses of P-protein slime in functional sieve tubes. We conclude that a subgroup of these proteins is involved in the formation of NPBs at least in the Malpighiales, and that these protein bodies have no role in rapid wound responses of the sieve tube network.

## Introduction

The structural differentiation of photosynthetic organisms into functionally distinct body parts depends on the ability to move the products of photosynthesis within the organism’s body (Knoblauch & Peters, 2013). In vascular plants, such long-distance transport of photoassimilates occurs in sieve tubes built of members called sieve elements (Behnke & Sjolund, 1990). Sieve elements are highly specialized cells that lose several cytoplasmic components including the nucleus during their differentiation (Heo, Blob & Helariutta, 2017). In most angiosperms, sieve element precursor cells produce significant amounts of phloem-specific proteins (Evert, 2006). These proteins assemble into dense bodies in the pre-sieve elements. In the most common course of development, these protein bodies disperse when the sieve elements mature (Cronshaw & Sabnis, 1990). However, the proteins released by the dispersion of the protein bodies do not dissolve in the translocation stream rushing through mature sieve elements, but rather form irregular masses visible in the conventional light microscope (Esau & Cronshaw, 1967; compare classical work, e.g., Strasburger, 1891). Both the irregular masses and the dispersive protein bodies from which the masses originate consist of filaments or tubular fibrils of varying sizes, as revealed by electron microscopy (Cronshaw, 1975). Therefore, the term *P-protein* was introduced to refer to both the irregular and the solid appearance of the structural phloem-specific proteins (Esau & Cronshaw, 1967). However, whether *P-proteins* represented a family of proteins with similar primary structure remained unclear.

Sieve tube occlusion following injury has been proposed as a function of P-proteins (Fischer, 1885; Ernst et al., 2012). Available evidence, however, is equivocal at best and the physiological role(s) of P-proteins remains obscure at this time (for critical discussion, see Sabnis & Sabnis, 1995; Knoblauch et al., 2014). A possible exception are forisomes, P-protein bodies restricted to the Fabaceae (bean family; Peters et al., 2010). Unlike the dispersive P-protein bodies (DPBs) commonly observed in pre-mature sieve elements, forisomes remain undispersed when the sieve elements mature (Laflèche, 1966; Lawton, 1978). Forisomes are capable of blocking sieve tubes (Knoblauch et al., 2012) due to their unique Ca^2+^-dependent but ATP-independent contractility (Knoblauch & Peters, 2004). Evidence concerning the physiological significance of this intriguing capability is ambiguous (Knoblauch et al., 2014), although an involvement of forisomes in responses to certain phloem-feeding aphids has been documented (Medina-Ortega & Walker, 2015). The identification of the component proteins of forisomes facilitated the molecular characterization of P-proteins in general. Forisomes consist of sieve element occlusion (SEO) proteins (Pélissier et al., 2008). While genes encoding SEO proteins are known exclusively from Fabaceae so far, very similar but unambiguously distinguishable SEO-related (SEOR) proteins and their genes are common in angiosperms (e.g., Pélissier et al., 2008; Lin et al., 2009; Huang et al., 2009; for review, see Knoblauch et al., 2014). The products of *SEOR* genes have been characterized in *Arabidopsis thaliana* (Froelich et al., 2011; Anstead et al., 2012), *Nicotiana tabacum* and *Cucurbita maxima* (Ernst et al., 2012). In all three cases, SEOR proteins form irregular masses in mature sieve tubes. In *Arabidopsis*, expression under the *AtSEOR2* promoter of a *GFP* (green fluorescent protein) gene tagged for ER retention of its product led to GFP fluorescence in sieve elements only (Rüping et al., 2010). Similarly, expression of *AtSEOR1* including its promoter fused with the *YFP* (yellow fluorescent protein) gene in *Arabidopsis* resulted in labeled protein exclusively in mature and developing sieve elements (Froelich et al., 2011). These findings suggest that the *Arabidopsis SEOR* promoters are active specifically in developing sieve elements that still have a functional transcription/translation machinery. Finally, no irregular P-protein masses were found in *SEOR* knock-out mutants of *Arabidopsis* (Froelich et al., 2011; Anstead et al., 2012). Thus, the irregular masses of proteinaceous phloem slime described in the older literature seems to consist of SEOR proteins, although we cannot exclude the participation of optional components unrelated to SEOR proteins such as the filament-forming phloem-specific proteins PP1 and PP2 (Clark et al., 1997). A certain complexity of the formation of P-protein masses is indicated by the fact that in *Arabidopsis*, both *AtSEOR1* and *AtSEOR2* are required; it is sufficient to knock out one of the two genes to prevent the development of P-protein accumulations (Anstead et al., 2012).

**Figure 1:**
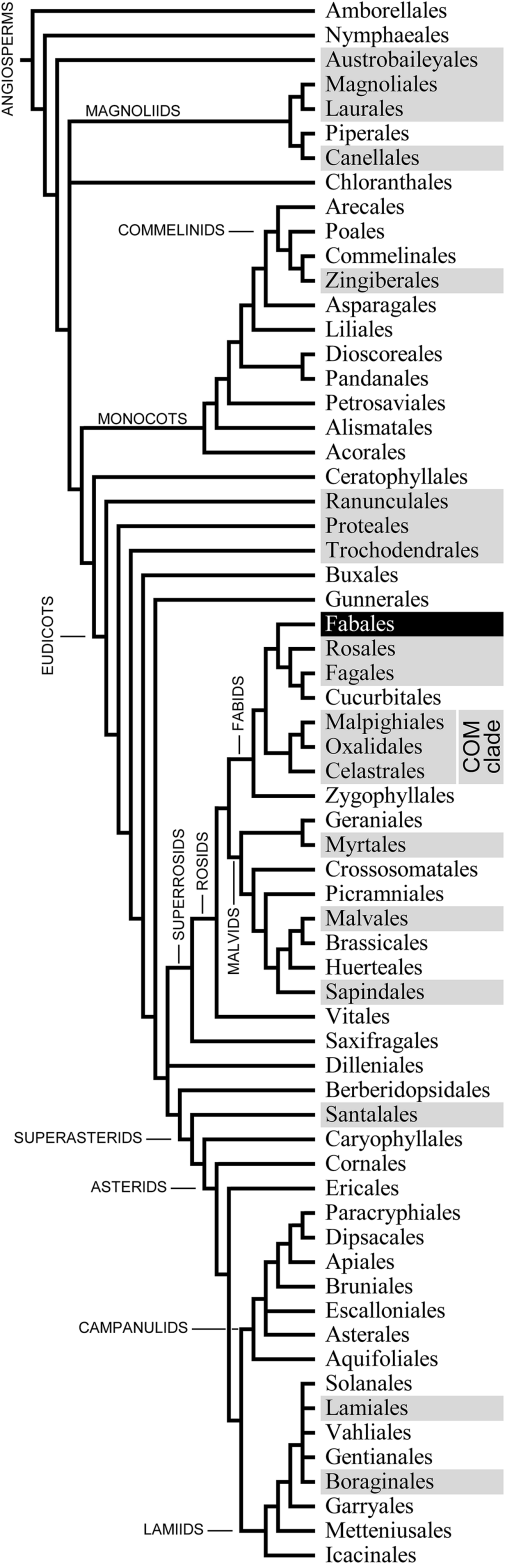
Phylogenetic relationships of the 64 orders of angiosperms according to Angiosperm Phylogeny Group et al. (2016; their Fig. 1). The 20 orders that include species known to possess non-dispersive P-protein bodies (NPBs; Behnke, 1991, 1994) appear on a dark background. One of these orders, the Fabales highlighted in negative print, includes the family Fabaceae, the only taxon known to possess contractile P-protein bodies (forisomes).

Before forisome contractility was discovered, these P-protein bodies of the Fabaceae had been considered examples of so-called non-dispersive P-protein bodies (NPBs; Behnke, 1991). In contrast to DPBs, which disperse during the differentiation of young sieve elements, NPBs retain their comparatively solid structure, thus deviating from the standard developmental pattern (Cronshaw & Sabnis, 1990). Initially NPBs were interpreted as remnants of the degradation of the nucleus, or “extruded nucleoli” (Esau, 1947; Kollmann, 1960; Mishra & Spanner, 1970), until ultrastructural and cytochemical investigations revealed their nature as protein agglomerations (Deshpande & Evert, 1970; Wergin & Newcomb, 1970; Ilker & Currier, 1975; Oberhäuser & Kollmann, 1977; Esau, 1978). A comprehensive compilation of information concerning the taxonomic distribution of NPBs was presented by Behnke (1991), but subsequent progress in angiosperm phylogenetics drove significant taxonomic revisions. Combining Behnke’s (1991, 1994) species-level information with the latest phylogenetic hypothesis on the level of taxonomic orders (Angiosperm Phylogeny Group: Angiosperm Phylogeny Group et al., 2016) produces an apparently random distribution of NPB occurrences across the cladogram (Fig. 1). This pattern may indicate that NPBs are more widely distributed than is known, and that the gaps reflect incomplete sampling. NPBs of two members of the order Rosales (*Rubus fruticosus*, Rosaceae; *Urtica dioica*, Urticaceae) have been subjected to functional tests and proved unresponsive to Ca^2+^ and sieve tube injury (Knoblauch et al., 2001). However, Rosales are not necessarily representative of all non-fabacean taxa with NPBs, and a wider taxonomic spread of functional data would seem desirable. In this context it should be noted that the molecular nature of non-forisome NPBs remains obscure at this stage; whether PP1 or PP2, SEO, SEOR, or other proteins are involved in forming these bodies is unknown.

NPBs were described in several species of the family Salicaceae in the Malpighiales (Crafts, 1939; Deshpande & Evert, 1970; Mishra & Spanner, 1970; Nehls, Schaffner & Kollmann, 1978; Behnke, 1991). Another member of the family, Black Cottonwood (*Populus trichocarpa*), has become an experimental “model tree” after its full genome data became available a decade ago (Tuskan et al., 2006). We expected this source of molecular information to facilitate the identification of the protein(s) forming NPBs in *P. trichocarpa*. First, we characterized the protein bodies in this species cell biologically and ultrastructurally, so that they could be compared to those of other Malpighiales in order to establish a comparative basis for possible generalizations of our anticipated functional and molecular results. We then tested the responsiveness of *P. trichocarpa* NPBs to stimuli known to induce forisome contraction. Finally, we performed mass isolation of these protein bodies, leading to the identification of a homolog of AtSEOR1 as their main (or only) component.

## Methods

### Plant material

*Ceiba pentandra* (L.) Gaertn., *Theobroma cacao* L. (both Malvaceae, Malvales), *Passiflora incarnata* L. (Passifloraceae, Malpighiales), *Pombalia* (*Hybanthus*) *communis* [A.St.-Hil.] Paula-Souza and *Viola tricolor* L. (both Violaceae, Malpighiales) were permanently available through the instructional plant collection of the School of Biological Sciences, Washington State University. *Populus trichocarpa* Torr. & A. Gray ex Hook (Salicaceae, Malpighiales) was grown in the open on the WSU campus (46°44′34″N, 117°8′10″W; elevation 789 m). *Vicia faba* L. (Fabaceae) was grown in a greenhouse as described before (Knoblauch et al., 2003).

### Light microscopy

Shallow periclinal cuts were made on the main vein of detached leaves to access active sieve tubes (compare Knoblauch & Van Bel, 1998). The leaves were fixed upside down on microscope slides with double-sided adhesive tape. Cut surfaces were covered with standard medium (10 mM KCl, 10 mM CaCl_2_, 5 mM NaCl, 50 mM HEPES at pH 7.5) or Ca^2+^-free medium (10 mM Na_2_-EDTA instead of CaCl_2_). Sieve elements were observed with a Leica DM LFSA microscope equipped with water immersion lenses not corrected for coverslips (HCX Plan APO U-V-I series) and a Leica DFC 300 FX camera (Leica Microsystems, Wetzlar, Germany). Confocal laser-scanning microscopy was performed with a Leica TCS SP8 (Leica Microsystems, Wetzlar, Germany) on specimens stained with aniline blue and synapto red.

### Electron microscopy

For scanning electron microscopy (SEM), pieces of *P. trichocarpa* mature petioles and leaf main veins 3 cm long were fixed for 24 h in 3% glutaraldehyde and 2% paraformaldehyde in either 50 mM cacodylate buffer, standard medium, or Ca^2+^-free medium (see above). The tissue was rinsed with cacodylate buffer and postfixed for 2 h in 2% OsO_4_ in 50 mM cacodylate buffer. After rinsing two times in 50 mM cacodylate buffer and two times in water, the specimens were lyophilized for 12 h using a VirTis lyophilizer (VirTis, Gardiner, NY, USA) and mounted on aluminum specimen mounts with carbon Pelco tabs (TED Pella, Redding, CA, USA). Approximately 20 nm gold were applied with a Technics Hummer V sputter coater (Anatech, Hayward, CA, USA), and specimens were examined with a Quanta 200F SEM (FEI, Hillsboro, OR, USA).

For transmission electron microscopy (TEM), excisates of 3 × 3 × 3 mm from petioles and leaves were fixed as described above. Ultrathin sections (70–100 nm) were produced from tissue embedded in Spurr’s resin with an ultramicrotome (Reichert Ultracut R; Leica Microsystems, Wetzlar, Germany) and were transferred onto formvar-coated slot grids. After staining with 1% uranyl acetate and 0.01% KMnO_4_ for 10 min and poststaining for 6 min in Reynolds’ lead citrate (Reynolds, 1963), the sections were imaged on a Philips CM200 and on an FEI Tecnai G2 20 Twin (FEI, Hillsboro, OR, USA).

For analysis by electron tomography, sections of about 150 nm were taken from the resin-embedded samples and placed onto formvar-coated grids. A single axis tilt series of ±55° was taken at 50,000 magnification on an FEI Tecnai G2 20 Twin (FEI, Hillsboro, OR, USA) using a high tilt holder. Images were taken every 2° to ±30°, and every 1° at higher angles. The pixel size of the original images (4,096 × 4,096 pixels) was 0.22 nm. The tomographic volume was acquired using FEI Automated Tomography software and reconstructed with Inspect3D after binning by four to produce a final tomography of 512 × 512 pixels with a pixel size of 0.88 nm. A voltex rendering of the reconstructed tomography was performed with Amira 2.0 (FEI, Hillsboro, OR, USA).

### Isolation of individual NPBs

Individual NPBs were isolated from sieve elements using fine-tipped (1–2 μm tip diameter) borosilicate glass capillaries (Clark Electromedical Instruments, Reading, UK) produced with a microelectrode puller (Model P-200; Sutter Istruments, Novato, CA, USA). The capillaries were mounted on a four-axis micromanipulator (MMW-204; Narishige, Tokyo, Japan) and the protein bodies were extracted from sieve elements as detailed by Knoblauch et al. (2003).

### Mass isolation of NPBs and molecular characterization

Segments of 30 cm length from de-leafed *P. trichocarpa* stems of about 2 cm diameter were scored through the bark into the secondary xylem with a single edged razor blade. Bark strips were peeled from the wood; the phloem was scratched from the periderm using surgical scalpels and placed in extraction buffer (10 mM Tris–HCl at pH 8.5, 200 μM EDTA, 8 mM K_2_SO_3_) in a mortar. The isolated phloem was squeezed with a pestle for 3 min, and the tissue was strained using 20 μm CellMicroSieves (BioDesign, New York, NY, USA). The remaining tissue in the sieve was ground three more times in fresh extraction buffer, progressively applying more pressure with the pestle in each round. The crude NPB extraction was centrifuged at 100 × *g* for 10 min. The supernatant was discarded and the pellet re-suspended in the extraction buffer and 3.75 ml 80% Nycodenz (Axis-Shield PoC, Oslo, Norway) solution. Using another 15 ml Nycodenz solution, a density gradient (20–80% Nycodenz) was produced with a custom-built gradient mixer. The NPB solution was applied to the gradient and centrifuged at 1,650 × *g* and 20 °C for 3 h (5810R centrifuge; Eppendorf, Hamburg, Germany). One milliliter aliquots were transferred consecutively from the top of the gradient into 2 ml microcentrifuge tubes. Small sub-samples were checked microscopically. The tubes containing the samples with the highest NPB densities were centrifuged (22,000 × *g* at 20 °C) for 10 min to obtain purified NPBs.

The purified proteins were denatured at 98 °C for 10 min in 300 μl loading buffer (62.5 mM Tris–HCl at pH 6.8, 10% glycerol, 2% sodium dodecyl sulfate, 5% β-mercapto-ethanol, 0.05% bromophenol blue) and separated by SDS–PAGE. The gel consisted of a 4% stacking gel (pH 6.8) and a 12% separating gel (pH 8.8). The proteins were separated for 45 min at 15 mA and variable voltages, and then fixed in the gel and stained by incubation in five parts methanol/five parts distilled water/one part glacial acetic acid and 0.05% coomassie blue overnight at room temperature. The gel was destained with the same solution lacking coomassie blue, and rehydrated in distilled water. Protein bands were excised and a trypsin digest tandem mass spectrometry (MS–MS) analysis was performed at the Molecular Biology Core of the Center for Reproductive Biology (Washington State University, Pullman, WA, USA). The peptides obtained were blasted against the National Center for Biotechnology Information (NCBI; https://www.ncbi.nlm.nih.gov) database to determine possible protein matches. Two peptides matched sequences in the hypothetical protein referred to as Potri017G071000.1 in the current version (November 2017) of the *P. trichocarpa* genome database.

Based on the genomic sequence of the corresponding gene, primers (forward with a 5′ *Xba*I restriction site: 5′-TTCTCTAGAAGCCAATCTTCTTACTCAGC-3′; reverse with a 3′ *Hind*III restriction site: 5′-TTCAAGCTTCGCACTCAACAACATTATTTAG-3′) were designed to obtain the full-length DNA from our plants. Genomic DNA was isolated from 50 mg fresh tissue homogenized with liquid nitrogen. Genomic DNA was isolated with a commercial kit according to the manufacturer’s instructions (Promega #A1120, Madison, WI, USA). The gene was amplified from a 1:100 genomic DNA dilution (1 μM final primer concentration) for cloning into the AKK 1408 shuttle vector (Pélissier et al., 2008). The amplified band was gel purified (#20021, QIAGEN Sciences, Germantown, MD, USA) and digested with 1 U each of *Xba*I and *Hind*III fast digest enzymes (Fermentas, Baltimore, MD, USA) for 3 h at 37 °C. The AKK 1408 shuttle vector was also digested with the restriction enzymes and the gene was inserted; ligation was performed overnight at 4 °C with T4 DNA ligase (Invitrogen, Waltham, MA, USA) and the vector was transformed into DH5α *Escherichia coli*. Successful transformants were grown overnight at 37 °C on LB medium with 50 μg/ml ampicillin. When colony PCR confirmed insertion, plasmids were isolated from cultures incubated overnight with the Promega SV Wizard Miniprep kit according to manufacturer’s instructions (#A1330 Promega, Madison, WI, USA), and the gene was sequenced using the flanking *M13F*: 5′-GTAAAACGACGGCCAGT-3′ and *M13R*: 5′-GGAAACAGCTATGACCATG-3′ primer sites by Eurofins Genomics (https://www.eurofinsgenomics.com).

### Sequence data analysis

Amino acid sequences of proteins and genomic nucleotide sequences were downloaded from the NCBI (https://www.ncbi.nlm.nih.gov) and phytozome databases (https://phytozome.jgi.doe.gov) for further analyses. Sequence alignments for conserved structure comparisons were conducted using CLC Sequence Viewer v.7.8.1, or earlier versions (QIAGEN, Aarhus, Denmark) with default settings. Phylogenetic analyses of the SEO/SEOR gene family for angiosperms used all putative gene family members from the above databases with gene copies aligned with MUSCLE (Edgar, 2004). Maximum likelihood (ML) analyses were performed using the JTT model in RAxML (Stamatakis, 2006) with 100 bootstraps. The tree was rooted on the branch placing the single SEOR gene copy from *Amborella* sister to the rest of the angiosperm gene copies, based on the strong independent evidence for that relationship (Angiosperm Phylogeny Group et al., 2016).

## Results

### NPBs in sieve elements of *P. trichocarpa*

**Figure 2:**
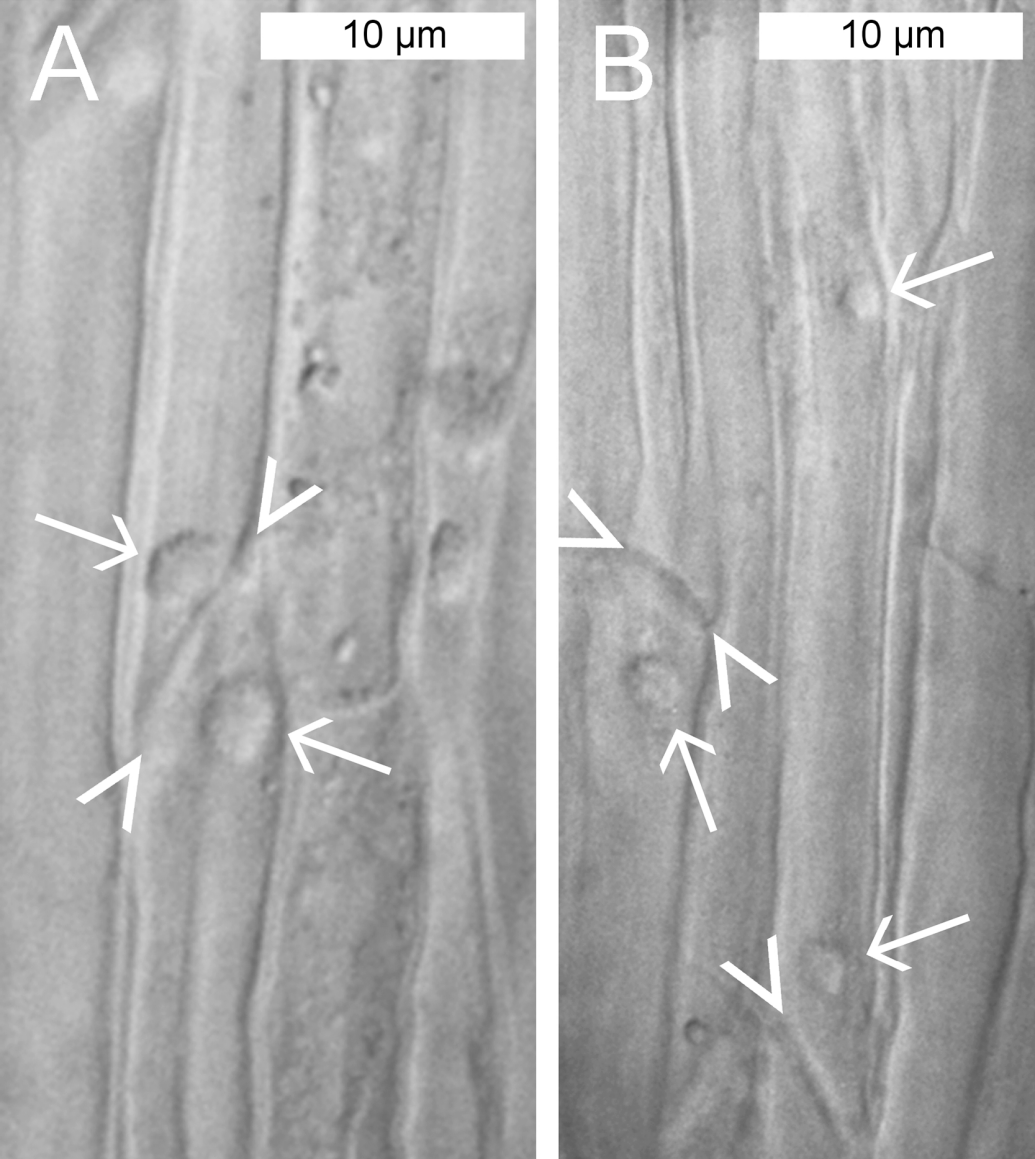
Standard brightfield micrographs of live sieve elements in *Populus trichocarpa* leaves, showing sieve plates (arrowheads) and non-dispersive P-protein bodies (NPBs; arrows). (A) NPBs located on both the upstream and downstream sides of one sieve plate. (B) On the left, an NPB is seen at the downstream side of a sieve plate. On the right, a rare case of a sieve element harboring two NPBs is seen. One of the bodies is located close to a sieve plate on its upstream side, the other at a distance in the center of the cell.

We applied conventional light microscopy to study the abundance and distribution of NPBs in the phloem of *P. trichocarpa*. A few cell layers were removed from the abaxial side of main veins of detached leaves to enable the observation of phloem tissue in situ. NPBs showed as spherical inclusions in live sieve elements (Fig. 2). Their diameters ranged from 1.8 to 5.5 μm and occupied between 25% and 50% of the diameter of their sieve elements (*n* = 28). Of over 120 sieve elements examined, about 90% contained one single NPB that was found close to a sieve plate (Fig. 2A). There was no consistency regarding the side of the sieve plate on which the NPB was located. Repeatedly we saw one NPB each on the upstream and downstream side of the same sieve plate (Fig. 2A), and NPBs at the upstream and downstream ends of sieve elements in parallel sieve tubes (Fig. 2B). About 3% of the sieve elements harbored two NPBs, and in roughly 10% an NPB was located at a distance from a sieve plate; an example of both features in the same cell is presented in Fig. 2B. No movements of NPBs ever were noticed.

### Structure of *P. trichocarpa* NPBs

**Figure 3:**
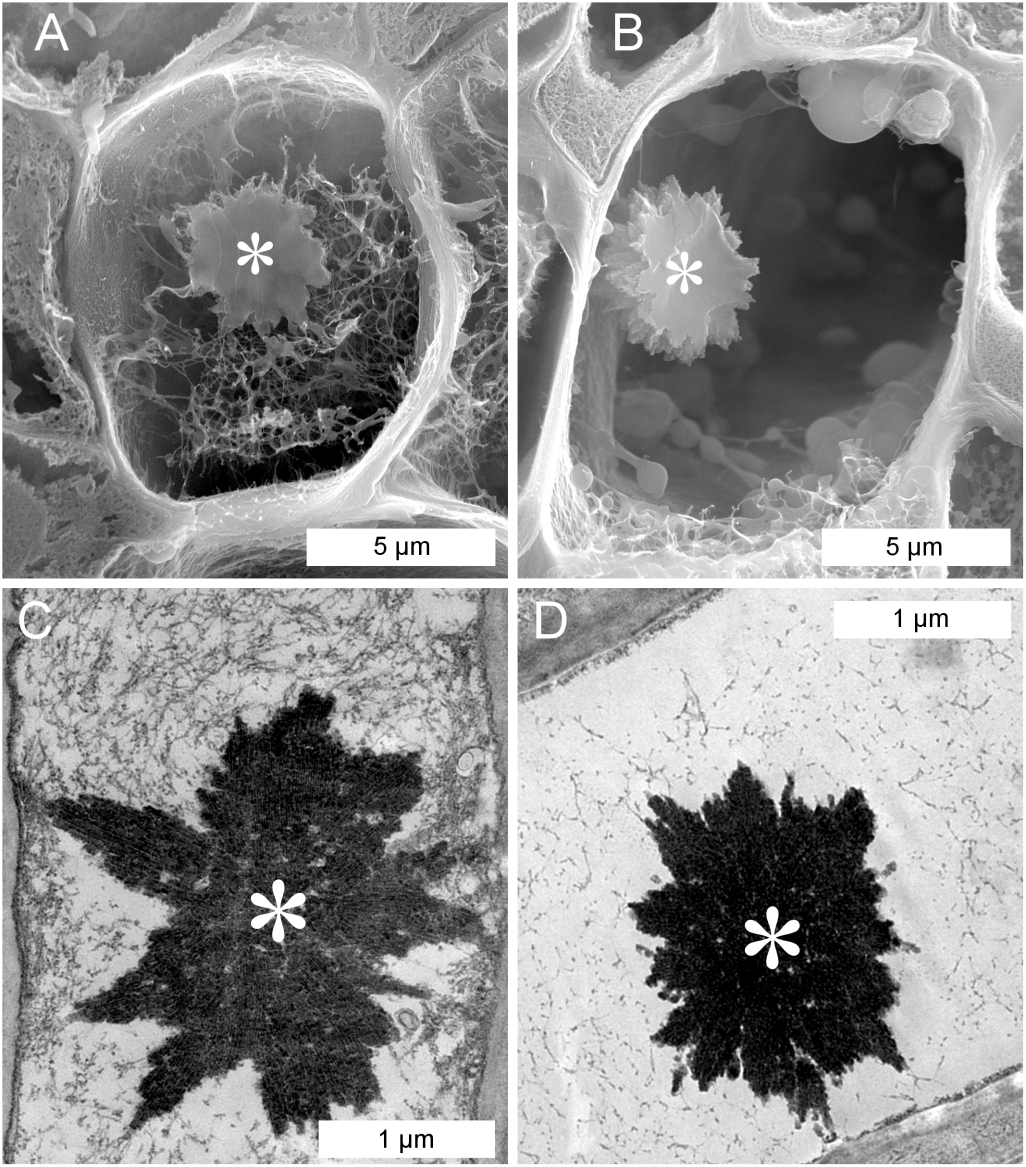
Scanning (A, B) and transmission (C, D) electron-micrographs showing non-dispersive P-protein bodies (NPBs) in sieve elements of *Populus trichocarpa*. In addition to NPBs (asterisks), sieve elements contain a network of fibrils of unknown identity; extreme cases with plenty (A, C) and few (B, D) of these fibrils are shown. Note that the NPBs in (A) and (B) are located in the planes of the cuts that opened the sieve elements; therefore we see the cut surfaces of the protein bodies.

In sieve tubes prepared for scanning electron microscopy, NPBs often appeared embedded in an irregular, fibrillar network of unclear identity that varied from relatively dense (Fig. 3A) to almost absent (Fig. 3B). Corresponding features were evident in transmission electron-micrographs. The lumen of the sieve elements around the NPBs exhibited various degrees of occupation by fibrillar structures from densely filled (Fig. 3C) to almost empty (Fig. 3D). We were unable to correlate this variation unequivocally with any obvious physiological or preparation-related parameter. Regardless of the presence of the irregular network in the sieve element, NPBs in both the TEM and SEM showed a rugged spiny surface (Fig. 3) that had not been resolved by in situ light microscopy (Fig. 2).

**Figure 4:**
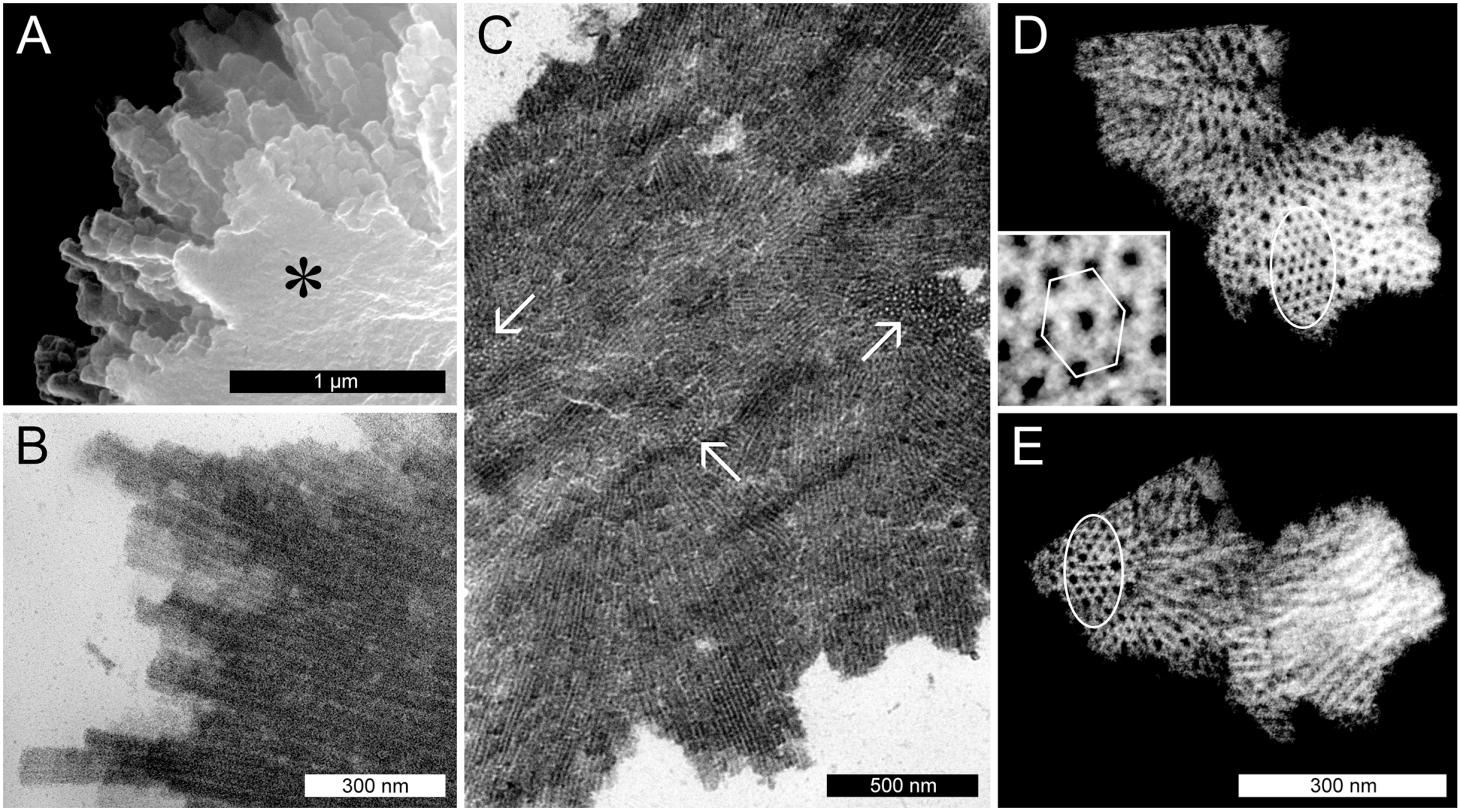
Ultrastructure of non-dispersive P-protein bodies (NPBs) from sieve elements of *Populus trichocarpa*. (A) Scanning electron-micrograph of rods with blunt tips that form the spiny surface structure of NPBs. The asterisk marks the cut surface of the NPB generated during preparation. (B) Longitudinal section (transmission electron-micrograph) of the surface rods, showing their longitudinal striation. (C) Section of an NPB viewed with the TEM. Numerous fields of locally co-aligned striations as well as groups of apparent globules (examples marked by arrows) are visible. The former are interpreted as bundles of tubules in longitudinal or oblique view, which reveal their tubular nature by showing as circular globules when cut more or less perpendicular with respect to their long axis. This interpretation is corroborated by electron-tomography of isolated NPB fragments (D, E): different parts of these fragments appear like regular arrays of globules (marked by ellipses in D and E), depending on the angle of view. The inset in (D) is a magnification from the marked zone that highlights the hexagonal arrangement of the tubules.

The spines on the surface of the *P. trichocarpa* NPBs consisted of groups of chimney-like rods, as revealed in the SEM. These rods differed in length and diameter, and terminated in blunt rectangular tips (Fig. 4A). In the TEM, the rods showed longitudinal striations (Fig. 4B). The striations continued into the interior of the protein bodies where bundles of rods appeared to be condensed into a solid mass. Consequently, cross-sections showed different zones of striations that varied in orientation (Fig. 4C). In rods that were sectioned perpendicular to their long axes it became evident that the longitudinal striations corresponded to tubules, since an electron-dense tubule wall could be distinguished clearly from an apparent cavity in the tubule center (examples marked by arrows in Fig. 4C). These tubules had outer and inner diameters of 20.8 ± 2.3 and 10.2 ± 1.7 nm (*n* = 50), respectively, suggesting a wall thickness of 5.3 nm. Electron tomography corroborated these findings and showed that groups of tubules in their most highly ordered state were packed in hexagonal arrays (Figs. 4D and 4E).

### NPBs in Malpighiales and Malvales do not respond to sieve tube wounding

**Figure 5:**
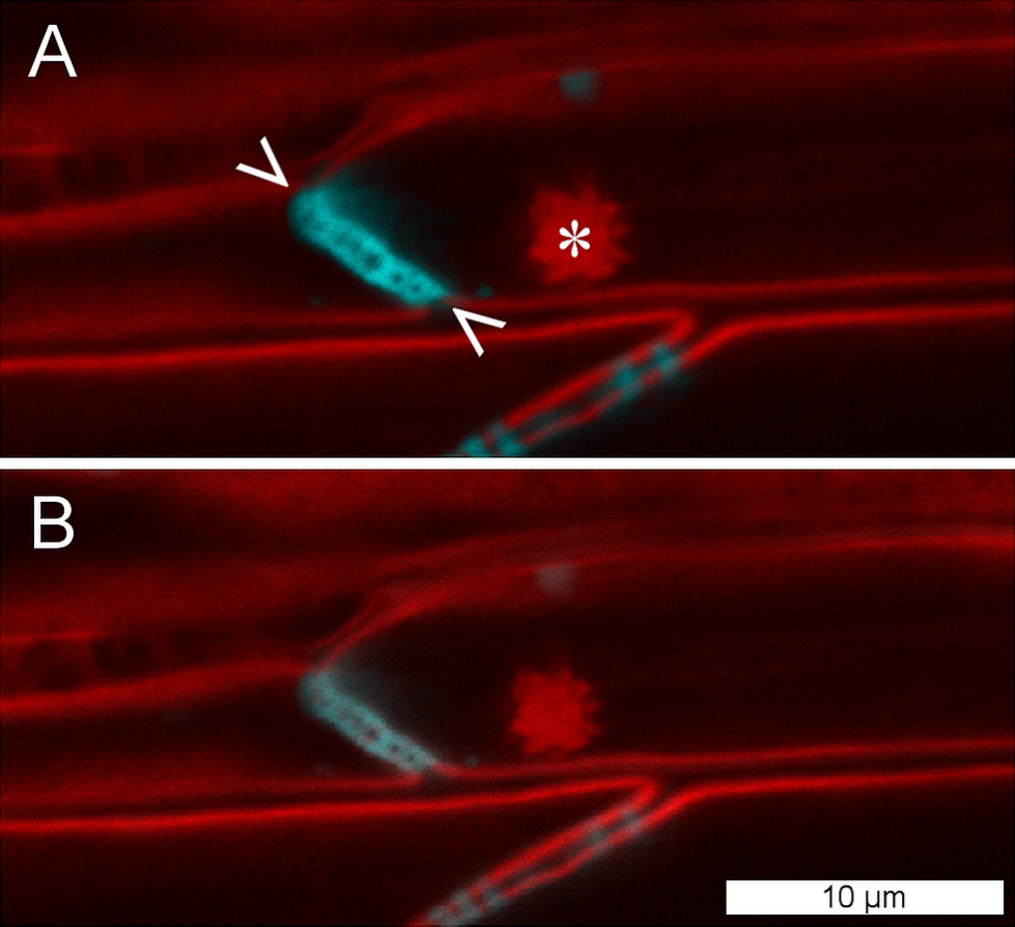
NPBs of *Populus trichocarpa* do not respond to sieve tube wounding. (A) In these CLSM micrographs, aniline blue, which stains callose, is rendered blue; it marks plasmodesmata and a sieve plate (between arrowheads). The plasma membrane and a non-dispersive P-protein body (NPB; asterisk) are stained by synapto red (rendered red). (B) The same cells after serious wounding of the tube by a microcapillary; the NPB shows no response. The experiment was repeated three times with identical results.

To establish whether *P. trichocarpa* NPBs were reactive to sieve element wounding like the forisomes of the Fabaceae (Fabales), we studied their responsiveness in situ by CLSM. NPBs appeared as spherical bodies of a diameter less than half that of their sieve element; the spiny surface structure was resolved (Fig. 5). We injured the sieve elements by puncturing individual sieve tubes with a dissection laser or with micro-pipettes. NPBs in the affected sieve elements never exhibited any response (Fig. 5 shows a representative example). To see whether this finding could be generalized for the order Malpighiales, we tested three additional members (*Viola tricolor* and *Pombalia communis*, Violaceae; *Passiflora incarnata*, Passifloraceae) with identical results. Moreover, NPBs in two members of the Malvales (*Ceiba pentandra* and *Theobroma cacao*, Malvaceae), more remotely related to Fabales but still in the rosid clade (Fig. 1), also failed to respond to any of the stimuli (see Fig. S1 for examples).

### Isolated *P. trichocarpa* NPBs are insensitive to Ca^2+^

**Figure 6:**
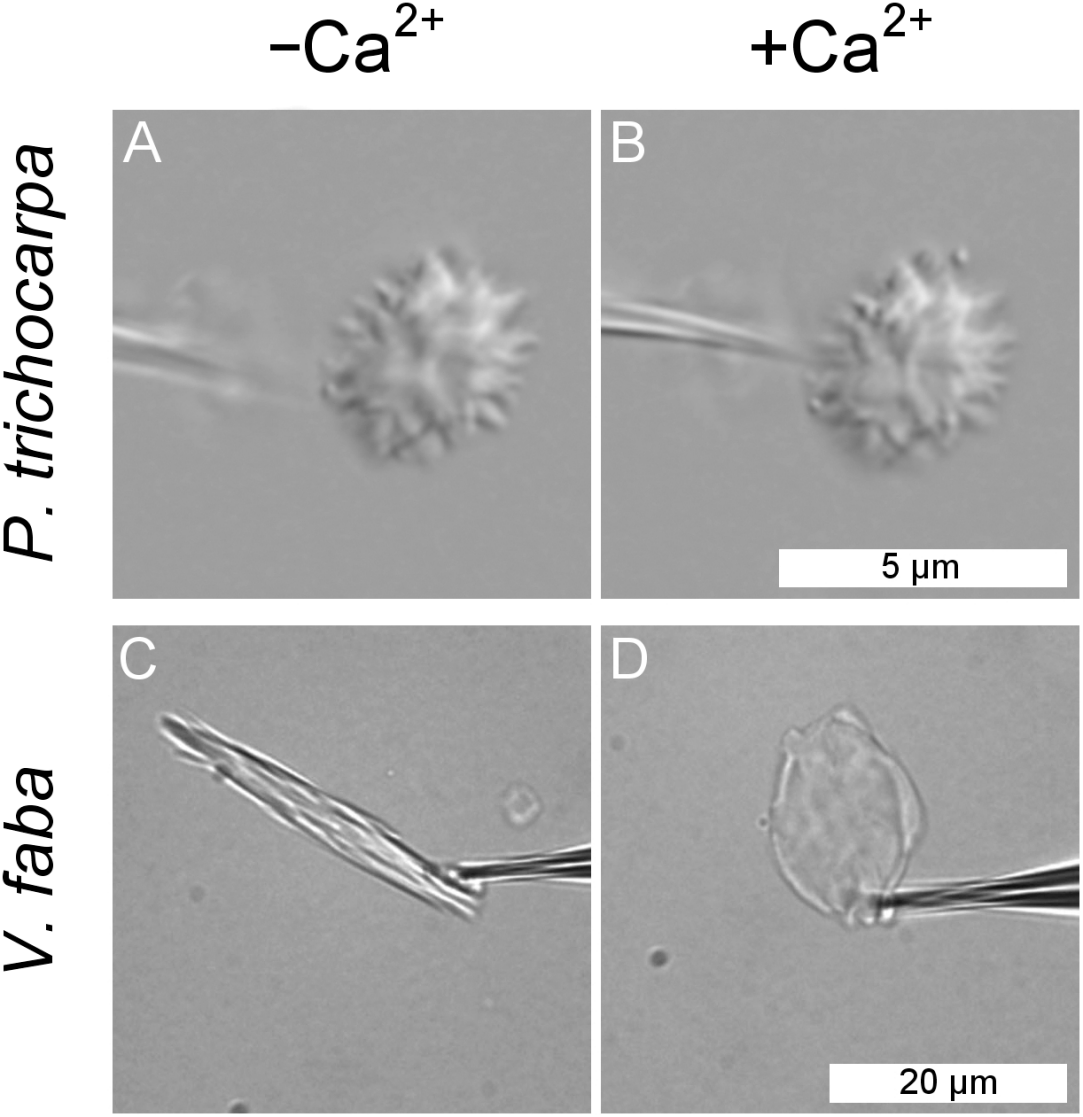
Lack of response of a non-dispersive P-protein body (NPB) isolated from *Populus trichocarpa* to calcium ions. An NPB is fixed on the tip of a micro-pipette and exposed to a calcium-free medium (A). Switching to a solution containing 10 mM Ca^2+^ (B) fails to evoke any response. The experiment was repeated four times with identical results. For comparison, the typical reaction of a forisome from *Vicia faba* also is shown (C, D).

Forisomes, the P-protein bodies of leguminous plants, respond to injuries of the sieve tube by a Ca^2+^-mediated swelling reaction. The fact that *P. trichocarpa* NPBs had not responded to sieve tube wounding (Fig. 5) did not necessarily rule out the possibility that they were reactive to Ca^2+^. We isolated individual NPBs mechanically and exposed them to Ca^2+^ concentrations of up to 10 mM. The NPBs remained unresponsive under conditions that triggered the full response in forisomes from *Vicia faba* (Fig. 6).

### *P. trichocarpa* NPBs consist of a SEOR protein

When isolated and purified *P. trichocarpa* NPBs were subjected to SDS–PAGE, they gave rise to two bands of just below and above 80 kDa, respectively. These bands were excised and analyzed by ESI MS–MS after trypsin digestion, leading to the identification of four peptides (1: LVSDSDIRK; 2: IPLEMVYVGK; 3: NKPLVVLDPQGK; 4: LVSDSDIYKLPYLHAIVK). BLAST searches failed to detect peptides 1 and 4 in the *P. trichocarpa* genome or any other genome database, and the origin and nature of these peptides remains obscure at this time. On the other hand, peptides 2 and 3 were identified in the amino acid sequence of the hypothetical protein, POPTR_0017s10720g, encoded by the gene called Potri.017G071000.1 in the current version 3.0 of the *P. trichocarpa* genome (https://phytozome.jgi.doe.gov). We cloned the gene from our plant material and sequenced it. The protein derived from the resulting DNA sequence had 716 amino acids and a molecular mass of 81.9 kDa. This result agreed well with the molecular masses of the protein bands derived from isolated NPBs, which supported the conclusion that the protein was a major component of *P. trichocarpa* NPBs.

**Figure 7:**
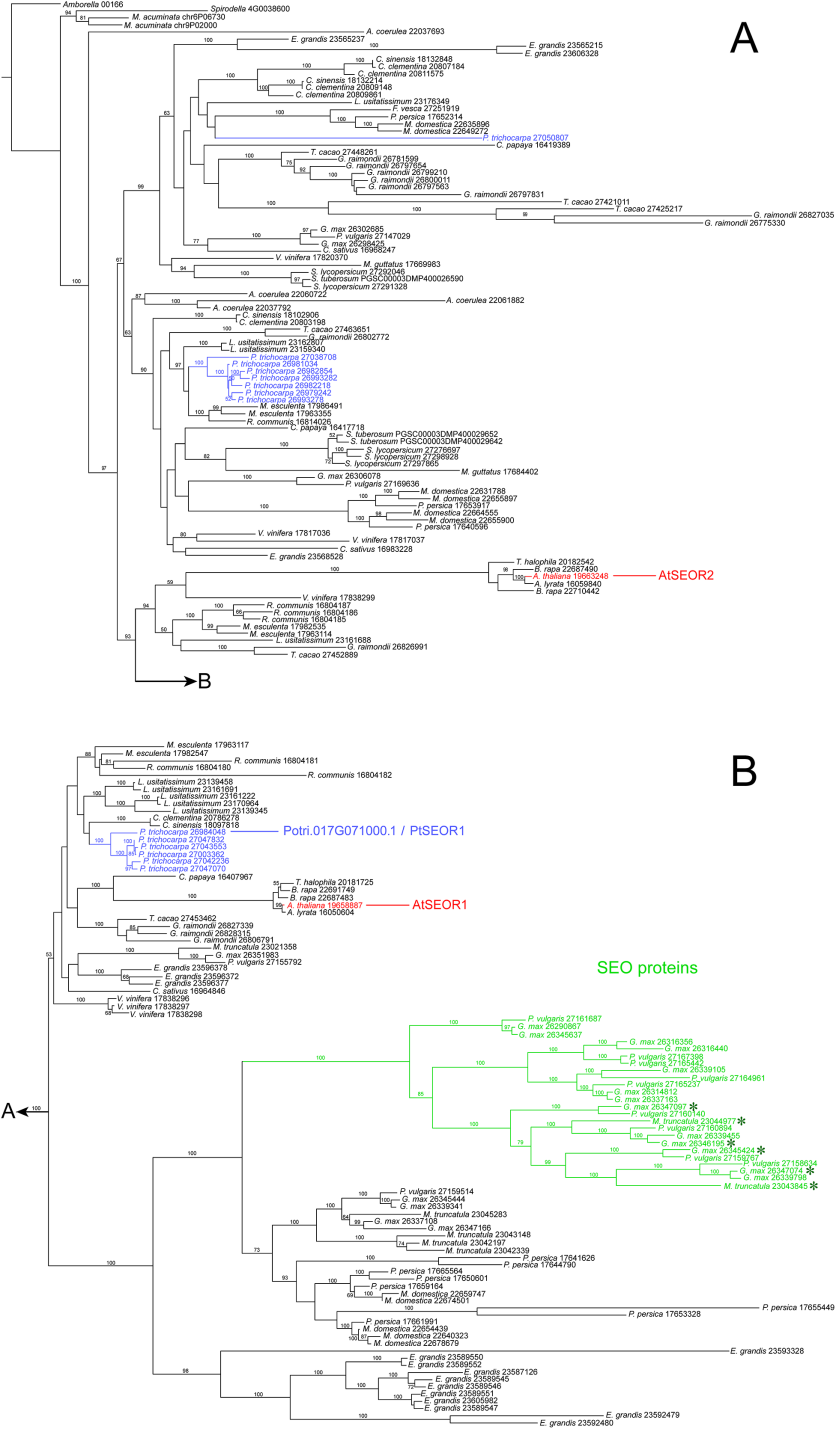
Phylogenetic hypothesis based on maximum likelihood analysis of 188 hypothetical SEO/SEOR proteins. (A) and (B) link to each other as indicated. Numbers above branches refer to bootstrap support. Hypothetical proteins from *Populus trichocarpa* (blue) and the two cytologically characterized SEORs from *Arabidopsis* (red) are highlighted. The new protein isolated from *P. trichocarpa* NPBs, called PtSEOR1, is positioned in the sister group of the branch that includes AtSEOR1. A single branch (green) includes all functionally characterized SEO proteins (forisome-forming proteins; asterisks) in our sample of sequences.

Our NPB protein was almost identical to the hypothetical product of Potri.017G071000.1 (Fig. S2), which in the genome database (https://phytozome.jgi.doe.gov) is annotated as an ortholog of At3g01680, the gene encoding the SEOR1 protein in *Arabidopsis*. Our rooted phylogenetic hypothesis of relationships among almost 200 hypothetical SEOR and SEO proteins from 29 species (Document S1) detected two clusters consisting of six and seven gene copies, respectively, of *P. trichocarpa*, while another gene sequence of this species assumed a position remote from the clusters (Fig. 7). Potri.017G071000.1 was localized in the six-protein cluster that was most closely related to AtSEOR1. We concluded that *P. trichocarpa* NPBs consist mainly or entirely of a P-protein whose primary structure closely resembles that of previously characterized SEOR proteins, in particular AtSEOR1. Consequently, we designated the new protein PtSEOR1.

## Discussion

### NPB localization in sieve tubes

Free-floating large objects exposed to the sieve tube flow will move to the downstream ends of sieve elements and accumulate there, at the upstream sides of sieve plates. In organs with a clearly defined expected flow direction, for instance mature photosynthesizing leaves, we would expect the objects to accumulate at the proximal ends of the sieve elements (i.e., the ends directed toward the petiole). This effect should be reinforced when a petiole is cut to obtain a leaf for microscopic examination (as in Fig. 2), because the opening of the sieve tube system will induce a turgor-driven surge of the sieve tube contents toward the cut. The NPBs in detached leaves of *P. trichocarpa* showed no such consistent localization. While they mostly were found close to sieve plates (Figs. 2 and 5), they were located randomly on the expected upstream or downstream sides of the plates. Consequently, sieve plates with one NPB on each side were observed regularly in *P. trichocarpa* (Fig. 2A). Similar observations have been reported from the related species *Passiflora coerulea* (Oberhäuser & Kollmann, 1977), *Salix nigra* (Deshpande & Evert, 1970) and *S. caprea* (Mishra & Spanner, 1970). The latter authors argued that the presence of NPBs (referred to as extruded nucleoli at the time) on both sides of sieve plates showed that no turgor-driven surge had been triggered by the preparation procedure. This seemed to imply further that no bulk flow had occurred in the sieve tubes prior to preparation, an important argument in favor of Spanner’s electro-osmotic theory of phloem transport (Spanner, 1979; for a review of the historical context, see Knoblauch & Peters, 2017). However, the unexpectedly inconsistent localization of NPBs under an assumed rapid bulk flow could be explained by structural anchors, as shown for various sieve element organelles (Ehlers, Knoblauch & Van Bel, 2000). It remains to be established whether an anchoring function can be ascribed to the irregular fibrillar networks in which NPBs seemed embedded to various degrees in preparations for electron microscopy (Fig. 3). The nature of these fibrils is obscure. One may suspect that they consist of structural cytoskeletal or SEOR proteins. However, the only such proteins detected in phloem exudates from hybrid poplar (*P. trichocarpa* × *P. deltoides*) were α- and β-tubulin (Dafoe et al., 2009). While hybrid poplar is not necessarily a valid model for its parent taxa, we note that the fibrils do not resemble microtubules (Fig. 3).

### NPB structure

Non-dispersive P-protein bodies of *P. trichocarpa* consist of numerous bundles of tubular protein fibrils (Fig. 4), resulting in a spiny surface of the NPB (Figs. 3A, 3B and 4A) and conspicuous striations visible in sections of NPBs in the TEM (Figs. 4B and 4C). Within each bundle, the fibrils form arrays with a hexagonal symmetry (Figs. 4D and 4E). All these structural characters have been documented previously in other species of the Malpighiales, including *Passiflora coerulea* (Passifloraceae, Kollmann, 1960; Oberhäuser & Kollmann, 1977) and the members of the Salicaceae, *Idesia polycarpa* (Behnke, 1991), *Salix caprea* (Mishra & Spanner, 1970), *S. sachalinensis* (Nehls, Schaffner & Kollmann, 1978), *S. nigra* and *Populus deltoides* (Deshpande & Evert, 1970). For the tubules forming NPBs in *S. sachalinensis*, Nehls, Schaffner & Kollmann (1978) reported an outer diameter of 17–18 nm and a wall thickness of 5–6 nm, similar to the values of 20.8 and 5.3 nm, respectively, we found in *P. trichocarpa*. These authors applied a negative staining protocol that allowed them to detect oblique striations on the walls of the tubules, prompting the hypothesis of a helical arrangement of substructures (Nehls, Schaffner & Kollmann, 1978). We were unable to reproduce this result in *P. trichocarpa*, despite significant efforts. Nonetheless, the pronounced similarities between NPBs of all Malpighiales examined suggest that these protein bodies are essentially the same structures in the species of this order.

The maximum packing density of cylinders is achieved by co-alignment in hexagonal arrays (Bezdek & Kuperberg, 1990). This conclusion holds when the cylinders are curved or flexible as is the case with cytoskeletal filaments and other proteinaceous fibrils (Starostin, 2006). Therefore the occurrence of hexagonal arrays in NPBs (Fig. 4) as such is not surprising. Somewhat irregular hexagonal patterns can be found even in the P-protein masses in *Arabidopsis* sieve tubes (Fig. S3; compare Froelich et al., 2011), which are capable of flowing along sieve tubes (Knoblauch & Peters, 2017). What calls for an explanation, though, is the difference in the stability of the filament arrays in *P. trichocarpa* NPBs as compared to the viscous sieve tube “slime” in *Arabidopsis*.

### Responsiveness of NPBs

Forisomes, the P-protein bodies of legumes, are SEO protein complexes that respond to injuries of the sieve tube by a Ca^2+^-mediated swelling (Knoblauch et al., 2001, 2003), which probably results in an occlusion of the tube (Knoblauch et al., 2012). Forisomes consist of fibrils arranged in pseudo-crystalline arrays (Laflèche, 1966; Lawton, 1978; Knoblauch et al., 2001). Poplar NPBs possess a similarly regular ultrastructure (Fig. 4), but seem to consist of a SEOR protein (Fig. 7). SEOR proteins have failed to produce any responses to Ca^2+^ or sieve element injury so far (Knoblauch et al., 2014). *Populus trichocarpa* NPBs showed no response to sieve element wounding in vivo (Fig. 5), and neither did NPBs in three other members of the Malpighiales. Together with *C*elastrales and *O*xalidales the *M*alpighiales form the COM clade, which is part of either the fabid branch (as shown in Fig. 1) or the malvid branch of the rosids, depending on whether the analysis is based on plastid DNA or low-copy nuclear and mitochondrial genes (Angiosperm Phylogeny Group et al., 2016). Therefore it is of interest that NPBs in two species of the Malvales also failed to respond to sieve tube injury. Considering also that NPBs in members of the Rosales, part of the sister clade of the Fabales, exhibited the same lack of responsiveness (Knoblauch et al., 2001), we conclude that NPBs of *P. trichocarpa* are representative of P-protein bodies of the entire rosid clade, except for Fabaceae, with respect to wound responses in vivo. Furthermore, *P. trichocarpa* NPBs in vitro did not respond to Ca^2+^ concentrations of up to 10 mM (Fig. 6), over a 100-fold the level required for the maximum forisome reaction under similar conditions (Knoblauch et al., 2005). Taken together, our findings provide no support for the assumption that SEOR proteins are involved in active wound responses or in any kind of Ca^2+^-mediated activities in live plants. They rather are in line with the hypothesis that Ca^2+^-dependent contractility of P-proteins is a synapomorphic trait of the Fabaceae (Peters et al., 2010), implying that the resulting possibility of an involvement of P-proteins in active wound responses is also restricted to this family. Unfortunately, this implies that the biological function of NPBs in *P. trichocarpa* and other species remains in the dark at this time.

### Relations between PtSEOR1 and other SEOR and SEO proteins

Two peptides derived from isolated *P. trichocarpa* NPBs also were found in the amino acid sequence of the hypothetical *P. trichocarpa* protein, Potri.017G071000.1. From the experimentally established sequence of the gene in our plants, we derived an amino acid sequence that was slightly shorter than that of Potri.017G071000.1 (716 compared to 752 residues), but exhibited identity with Potri.017G071000.1 in 712 of its 716 residues (99.4%; Fig. S2). Both sequences were closely related to that of AtSEOR1 (Fig. S2; Fig. 7). Taken together, these findings support the conclusion that an important or even the main component of NPBs in *P. trichocarpa* is a SEOR protein. This protein derived from live plants evidently represents the hypothetical Potri.017G071000.1, and we call it PtSEOR1.

The *P. trichocarpa* genome database contains several hypothetical proteins exhibiting sequence similarities with *Arabidopsis* SEORs, and thus with PtSEOR1. Phylogenetic analysis places most of these hypothetical proteins in two dense clusters of proteins with closely related primary structures (Fig. 7), which may reflect recent gene multiplication events. The promoter region of one of the corresponding genes, Potri.001G340200.1, recently has been shown to be active specifically in phloem tissue (Nguyen et al., 2017). Unfortunately, the resolution of the histological approach did not allow these authors to differentiate between the cell types of the phloem, and it remains unclear whether the promoter, denoted *PtrDP3*, is sieve element-specific like the promoters of *VfSEO1* (Noll et al., 2007), *AtSEOR1* (Froelich et al., 2011), *AtSEOR2* (Rüping et al., 2010) and *MtSEO1* (Bucsenez et al., 2012). Nguyen et al. (2017) determined that the sequence located within −200 to −300 bp from the translation initiation codon was sufficient to render the *PtrDP3* promoter phloem-specific. An alignment of the putative promoter sequence of the *PtSEOR1* gene Potri017G071000.1 and that of *PtrDP3* revealed a high degree of homology between the 100-bp sequence conferring phloem-specificity to the latter and the corresponding sequence of *PtSEOR1*, including a conserved TATA-box motif (Fig. S4). Therefore it seems a plausible hypothesis that the *PtSEOR1* promoter might be active specifically in the phloem as well. In the future, the comparative functional analysis of different *SEOR* promoters can be expected to help elucidating the mechanisms of phloem- and sieve element-specific gene expression.

## Conclusion

So far, SEOR proteins had been characterized as essential components of the irregular protein masses earlier researchers had observed in sieve tubes. Our study in *P. trichocarpa* revealed the first case of a SEOR protein forming part of or possibly even the entire structure of a non-dispersive P-protein body (NPB). Since NPBs of all Malpighiales resemble those in *P. trichocarpa* in consisting of similarly sized proteinaceous tubular fibrils densely packed in hexagonal arrays, the hypothesis is justified that they all are made of SEOR proteins. Intriguingly, when forisomes develop in immature sieve elements of legumes, significant parts of their bodies seem to form through compaction of coaligned tubular P-protein fibrils initially arranged in hexagonal arrays (Wergin & Newcomb, 1970; Palevitz & Newcomb, 1971). On electron-micrographs these arrays look conspicuously similar to the NPBs of the Malpighiales (compare micrographs in Wergin & Newcomb, 1970, with those in Oberhäuser & Kollmann, 1977; Nehls, Schaffner & Kollmann, 1978; Deshpande & Evert, 1970; and our Figs. 4C–4E). For analytical purposes, we therefore can subdivide the SEO/SEOR protein family into three functionally defined groups. First, SEOR proteins forming filaments that may arrange in more or less ordered symmetries (as in *Arabidopsis*, see Fig. S3), but which remain unstable and present themselves as viscous slime masses capable of moving with the flow. Second, SEOR proteins forming tubular fibrils that arrange themselves in hexagonal arrays stable enough to give rise to long-lived, non-fluid NPBs. Third, SEO proteins that resemble the previous type but in addition can condense into stable bodies exhibiting Ca^2+^-dependent contractility. On the molecular level, the structural basis for the differences between these proteins must be sought in the deviations of their amino acid sequences from the common pattern. Such informative deviations will become easier to identify with an increasing number of structurally and physiologically characterized SEOR/SEO proteins in a variety of species.

## Supplemental Information

10.7717/peerj.4665/supp-1 Supplemental Information 1 Examples of non-dispersive P-protein bodies (NPBs) that do not respond to wounding of sieve elements by puncturing with micro-pipettes. The CLSM micrographs were taken after a sieve element had been severed with a micro-pipette; arrowheads point to unresponsive NPBs. In all cases, the NPBs are located close to a sieve plate (compare Fig. 5). (A) *Theobroma cacao* and (B) *Pombalia communis* stained with aniline blue and synapto red. (C) *Viola tricolor* stained with CDMFDA. The micro-pipette is visible in this image (white arrows). Scale bars: 10 μm.

10.7717/peerj.4665/supp-2 Supplemental Information 2 Comparison of the hypothetical product of gene Potri.017G071000.1 (from *Populus trichocarpa* v3.0, https://phytozome.jgi.doe.gov/pz/portal.html), PtSEOR1, and AtSEOR1. Positions in which Potri.017G071000.1 and PtSEOR1 differ show in red, residues that are identical in the two are shown in blue. Positions in which AtSEOR1 matches the consensus of the *P. trichocarpa* sequences also appear in blue. The alignment was produced with CLC Sequence Viewer v. 7.8.1.

10.7717/peerj.4665/supp-3 Supplemental Information 3 TEM micrographs of a sieve element in an *Arabidopsis* leaf with bundles of SEOR protein filaments in longitudinal and perpendicular section. (A) cross-section of a sieve element with two slime masses consisting of filaments of SEOR protein (arrows). (B) zoom into one of the protein masses, showing longitudinal or oblique sections of the winding SEOR filaments as well as sections perpendicular to the filament axes. (C) zoom into that part of the protein mass where filaments are sectioned more or less perpendicularly. The distribution of the filament cross-sections does not follow a clear geometric pattern, indicating that the packing density does not approach its theoretical maximum. Nonetheless an early stage in the development of a hexagonal arrangement is suggested. For methods, see Froelich et al. (2011), Plant Cell 23, 4428–4445.

10.7717/peerj.4665/supp-4 Supplemental Information 4 Comparison of the putative promoter sequences of the *P. trichocarpa* genes Potri.001G430200.1 and Potri.017G071000.1, the *PtSEOR1* gene. Identical bases appear in blue, different bases and gaps are shown in red. The 100-bp sequence that confers phloem-specificity to the Potri.001G340200.1 promoter (Nguyen et al., 2017) and the corres-ponding sequence in Potri.017G071000.1, the *PtSEOR1* gene, are shown on yellow background. Of the 100 base pairs, 71 are conserved. Note the conserved TATA-box motif at position −228. The alignment was produced with CLC Sequence Viewer v. 7.8.1.

10.7717/peerj.4665/supp-5 Supplemental Information 5 Amino acid sequences of the proteins analyzed in Fig. 7.

## Additional Information and Declarations

### Data Availability

The following information was supplied regarding data availability: The amino acid sequences of all hypothetical proteins used in the analysis resulting in Fig. 7 are provided as a Supplemental Dataset.
