# Supplementary figures and images for: Non-dispersive phloem-protein bodies (NPBs) of Populus trichocarpa consist of a SEOR protein and do not respond to cell wounding and Ca2+
Source: PeerJ. 2018 Apr 17;6:e4665. doi: 10.7717/peerj.4665 (PMC5909683; doi:10.7717/peerj.4665)

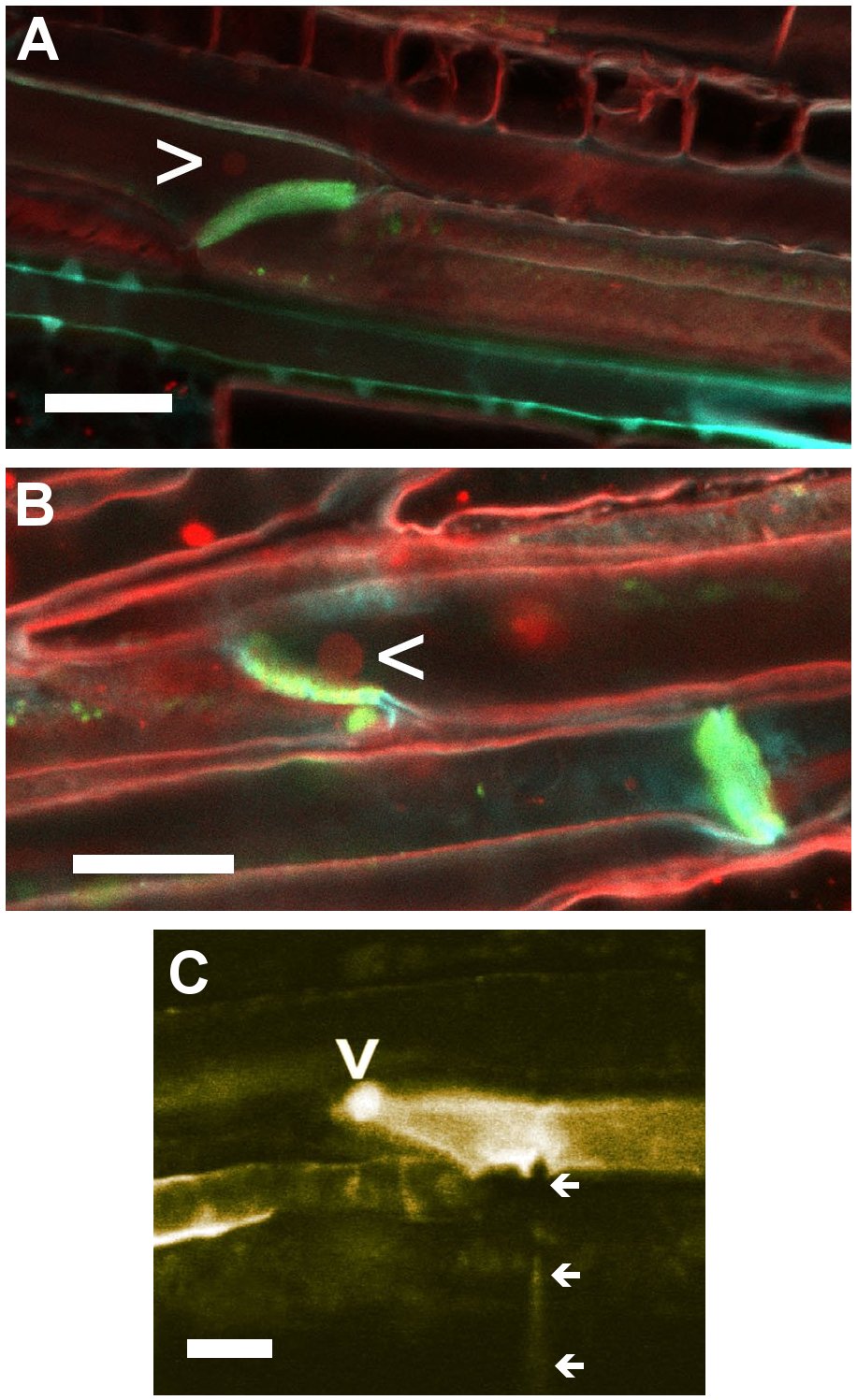

Supplement: Supplemental Information 1 — The CLSM micrographs were taken after a sieve element had been severed with a micro-pipette; arrowheads point to unresponsive NPBs. In all cases, the NPBs are located close to a sieve plate (compare Fig. 5). (A) Theobroma cacao and (B) Pombalia communis stained with aniline blue and synapto red. (C) Viola tricolor stained with CDMFDA. The micro-pipette is visible in this image (white arrows). Scale bars: 10 μm. [file peerj-06-4665-s001.jpg]

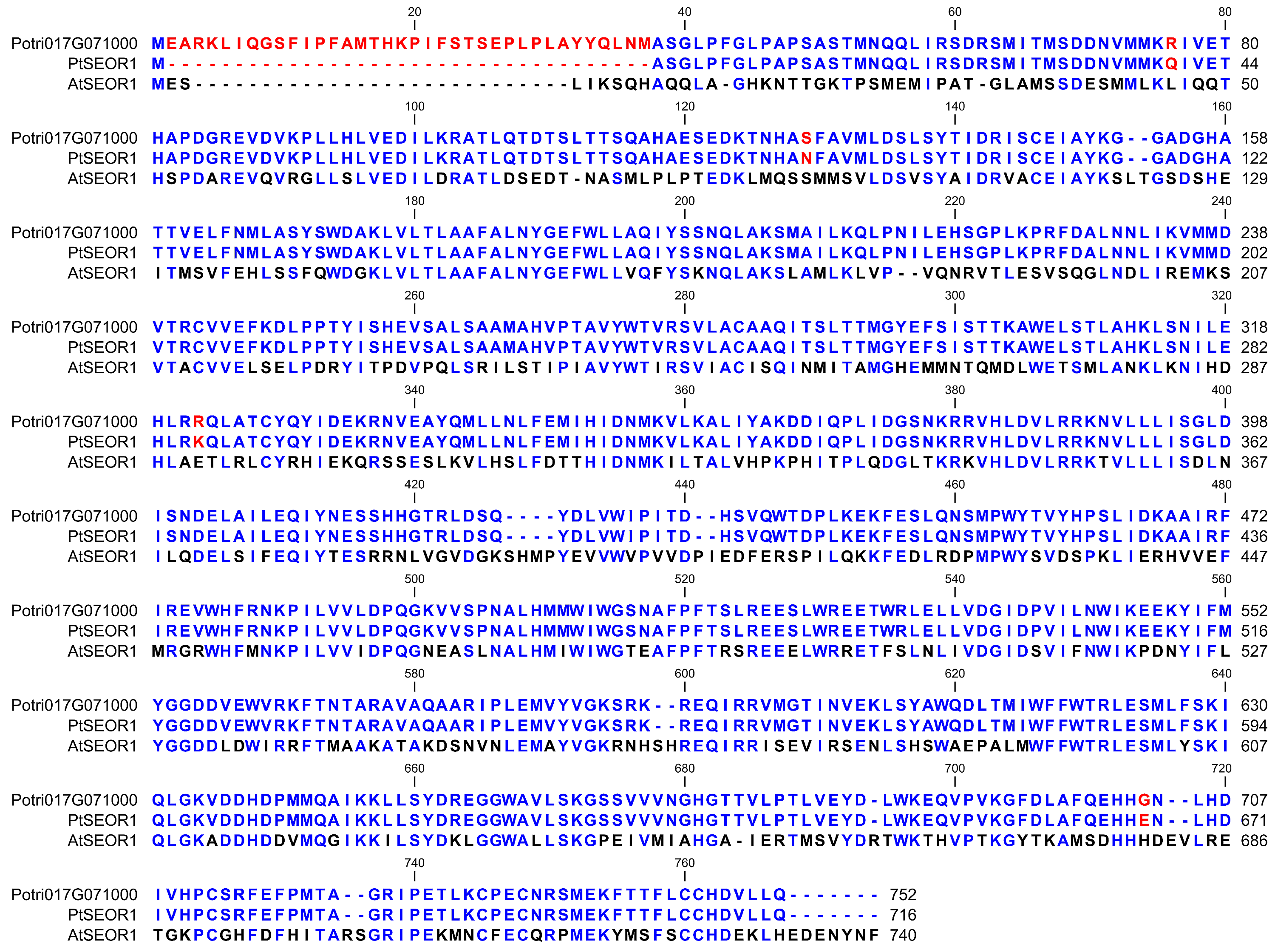

Supplement: Supplemental Information 2 — Positions in which Potri.017G071000.1 and PtSEOR1 differ show in red, residues that are identical in the two are shown in blue. Positions in which AtSEOR1 matches the consensus of the P. trichocarpa sequences also appear in blue. The alignment was produced with CLC Sequence Viewer v. 7.8.1. [file peerj-06-4665-s002.png]

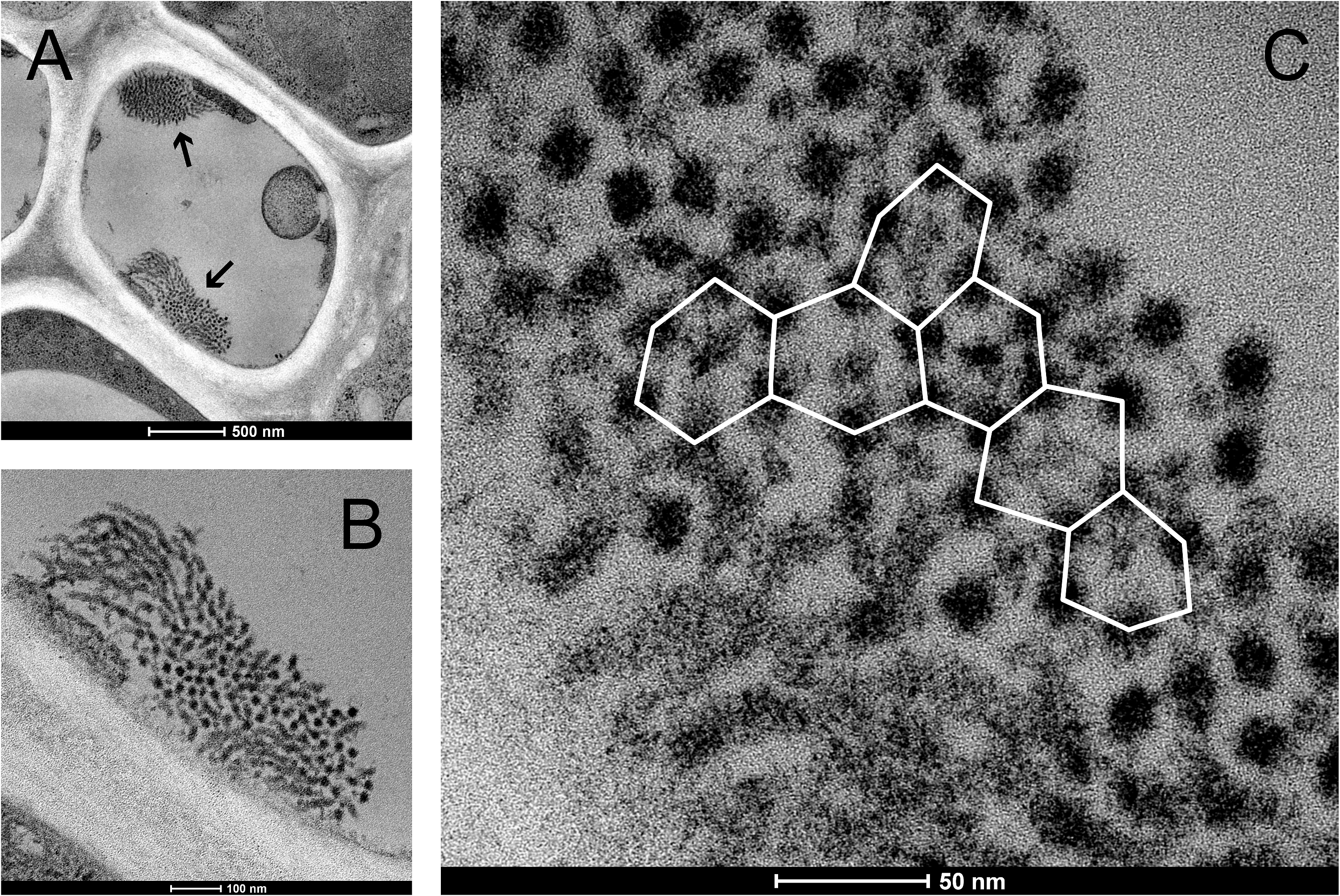

Supplement: Supplemental Information 3 — (A) cross-section of a sieve element with two slime masses consisting of filaments of SEOR protein (arrows). (B) zoom into one of the protein masses, showing longitudinal or oblique sections of the winding SEOR filaments as well as sections perpendicular to the filament axes. (C) zoom into that part of the protein mass where filaments are sectioned more or less perpendicularly. The distribution of the filament cross-sections does not follow a clear geometric pattern, indicating that the packing density does not approach its theoretical maximum. Nonetheless an early stage in the development of a hexagonal arrangement is suggested. For methods, see Froelich et al. (2011), Plant Cell 23, 4428–4445. [file peerj-06-4665-s003.jpg]

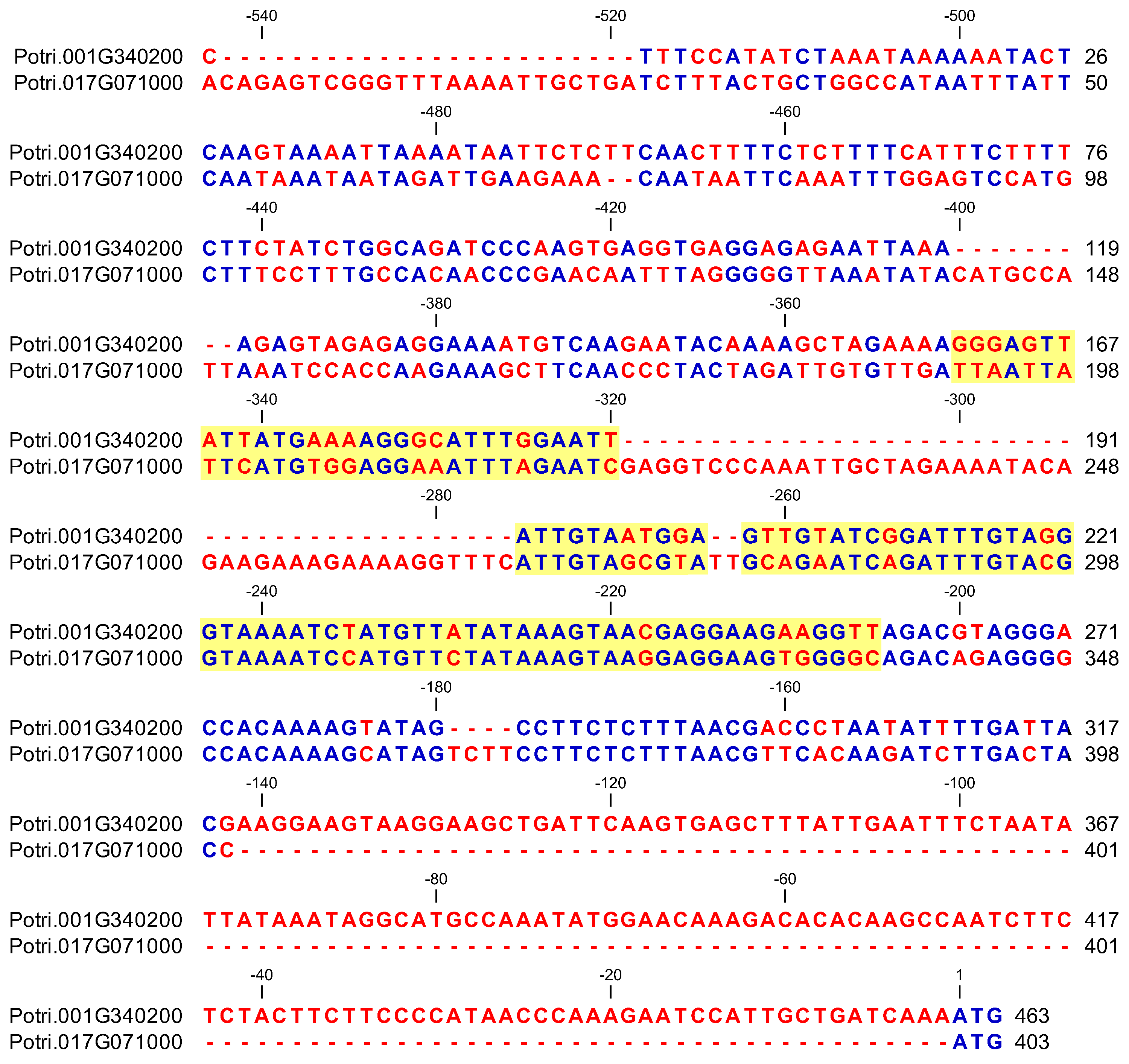

Supplement: Supplemental Information 4 — Identical bases appear in blue, different bases and gaps are shown in red. The 100-bp sequence that confers phloem-specificity to the Potri.001G340200.1 promoter (Nguyen et al., 2017) and the corres-ponding sequence in Potri.017G071000.1, the PtSEOR1 gene, are shown on yellow background. Of the 100 base pairs, 71 are conserved. Note the conserved TATA-box motif at position −228. The alignment was produced with CLC Sequence Viewer v. 7.8.1. [file peerj-06-4665-s004.png]
