# Supplementary material for: Non-dispersive phloem-protein bodies (NPBs) of Populus trichocarpa consist of a SEOR protein and do not respond to cell wounding and Ca2+
Source: PeerJ. 2018 Apr 17;6:e4665. doi: 10.7717/peerj.4665 (PMC5909683; doi:10.7717/peerj.4665)
Supplement: Supplemental Information 5 [file peerj-06-4665-s005.docx]

**Amino Acid Sequences Analyzed for Figure 7**

Species are listed at the end of this document.

**16050604_peptide|Alyrata|477347|477347**

MESLIKSQHAQQLAGHKNTTGKTPSMEMIPATGLAMSSDENMMLKLIQQTHSPDAREVQVRGLLSLVEDILDRATLDSEDTNASMLPLPTEDKLMQSSMMSVLDSVSYAIDRVACEIAYKSLTGSDAHEITMSVFEHLSSFHWDGKLVLTLAAFALNYGEFWLLVQFYSKNQLAKSLAMLKLVPVQNRVTLESVSQGLNDLIREMKSVTACVVELSELPDRYITPDVPQLSRILSTIPIAVYWTIRSVVACISQINMITAMGHEMMNTQMDLWETSMLANKLKNIHDHLAETLRLCYRHIEKQRSSESLKVLHSLFNTTHIDNMKILTALIHPKPHITPLQDGLTKRKVHLDVLRRKTVLLLISDLNILQDELSIFEQIYTESRRNLVGVDGKSHMPYEVVWVPVVDPIEDFERSPILQKKFEDLRDPMPWYSVDSPKLIERHVVEFMRGRWHFMNKPILVVIDPQGNEASLNALHMIWIWGTEASPFTRSREEELWRRETFSLNLIVDGIDSVIFNWITPDNYIFLYGGDDLDWIRRFTMAAKATAKDSNVNLEMAYVGKRNHSHREQIRRISEAIRSENLSHSWAEPALMWFFWTRLESMLYSKIQLGKADDQDDVMQGIKKILSYDKVGGWALLSKGPEIVMIAHGAVERTMSAYDRTWKTHVPTKGYTKAMYDHHHDEVLRETGKPCGHFDFHITARSGQIPEKMMCFECQRPMEKYMSFSCCHDEKLHEDENYNF

**16059840_peptide|Alyrata|477348|477348**

MAQRFQLNPKTLPTADPLKRVSLIPRSAEQKLADNSGERRPLAPRTHEDSPFGDHTDDHNVSAPADHNKVMDHETEKLGSIVPKTANHPHPSEDVLDANIRHSMVPKSLGHNSLGGRFGPGKKQAFHRNGRPMFSLSDDRVMADRVLKTHSPDMIFFDVKSLLSVVDDIFKSHVPSVDDSAPKPTLVFKDYADHTSFETFADVIDQISCEIDCKCLHGGESHGMMTSGLHLDSRNTTTFSVLSLVSKYRWDAKLVLVLAALAVKYGVFLLLAETHATNQLTKSLALIKQLPSIFSRQNALHQRLDKTRLLMKEMVALTTTIIEIYQLPPNHITTAFTDHVPTAVYWIVRCVLICVSHLSGASGFRQDQIMSFMEVSEIHENSERLRKINDYLKEQLRKSRLTIEDGIIEEEYQELIQTFTTIIHVDVVPPLLRLLRPIDFLYHGAGVSKRRVGINVLTQKHVLLLVSDLENIEKELYILESLYTEAWQQSFEILWVPVQDFRTEADDAKFEALHMNMRWYVLGEPRKLRRAAIRFVREWWGFKNRPILVALDPKGQVMSTNAFPMVWIWQPFAHPFTTARERDLWSEQEWNLEFLIDGTDPHSLNQLLDGKYICLYGGEDLQWIKNFTSLWRNVAKAANIQLEMVYVGKRNPKNGILPIINTIRDENISHTLPDLFQIWFFWTRIESMWESKQRMLKARGIKGREGFKEEEKDLVLQEVVAMLGYGGEGDGWGLVSKASDLMVRAKGNLFSRGLSEFNEWEVNIPTKGFLTALNDHLLMRLPPHHCTRFMLPETAGIIPNEVECTECRRTMEKYYLYQCCLE

**16407967_peptide|Cpapaya|evm.TU.supercontig_130.60|evm.model.supercontig_130.60**

MDLLLKSVATAGQQKPATSNGKSMLTASNENLANKPATAAADQKLMESDRKIVSPPRDILGLIRSNPSSSQKLVKSTDHRSMLTMSDDTMMMKQIQATHTPDGREVEVRPLFGLVEDILNRSTLPIDAVLAGPQKHMENLEDKSDQASFIDMLEALSFTIDRIACEIAYKALSGSDVHATTMSLCHLLSNYQWDAKLVLTLAAFALNYGEFWLLAQIYSSNQLAKSMAILKQVPNILEHSAALRSKFNALNTLIKVMMDVTRCIVEFRELPDAYISEEVPEFSTAMAHIPTAVYWTIRSAVACAAQITMITSMSHELAYSPTESWELSTLAHKLNTIHDHLNERLKLCNRFIEEKKNLEALKMLHDLFRMTHIDNMRVLKAIIYAKDDVQPLVDGGANMKRVNLEVLRRKNVLLLISGLNISQDELSILEHIYTESRQHLTRPERDGLFELVWIPIVDPAVEWNEKQSLQNRFEALQQTMPWYSVYHPKIIQKPVIDFVKEEWHFRNKAILVVLDPQGKVVSPNAIHMMWIWGSAAFPFTSLKEETLWREETWRLDLLVNGIDQVILNWIREGRYIFLYGGEDIEWIRKFTSAARFVARDSNIPLEMVYVGKSSHREQVRKVTATIAAEKISHCWQDPAMVWFFWTQATKHVVLQDPARQSR

**16417718_peptide|Cpapaya|evm.TU.supercontig_33.72|evm.model.supercontig_33.72**

MASSQSQLVPQQPPAGRRRERFLFSSSDDNAMIKQIEATNAPDDRDFTVKPLLEIVEDIFHRSTPAAPGVALEMKSLEAVDEKALHSGFDDMFEFVSRTIYRIYCELGSKCFSAADAHTTTIAILNILSNYKWDAKVVLSLAGFALIYGEFWLDAQLYQTNPLAKGVAILKHLPTILERSDTLKPRLEAVTKLIRAMLDVSRCIVEFKQLPSQYVAQDSPELSTATAHIPIAVYWTIRSTIASAAQIIGLIGMGYEFIASTQETWELLSLAHKLSSIHDHLNDQLKICHQLIEERKHIEAYQTLVRLFKTAHIDNMKILRALFYAKDGLPLYNCSSGKKVSIDVLKKKLVLLFISDLELSVDELQILLLMYNESKQNMRPESEYEVVWVPVVDRSVAWNEERQKHFEYLQSMMPWYMVNHPSIIDPAVIRYIKEEWLFNKKPLLVVLDPQGREVNRNAIHMMWIWGGLAYPFTRAREEAIWKQQSWSLELLADNIEPLIFTWMKDGKYICLYGGEDIEWIRKFTVTAQAVAEAAKIGLEMLYVGKSNPRERVRKNNAIIQVETLSHTLPDLTLIWYFWVRLESMWHSKTQQDKQVDDDPIMQEVMTMLSFDGSDIGWAVIFKGSNEMARAKGDTFLKCLNEYTLWKDRVGPNGFVPAMNEHLDAIHSPHHCNRLILPGTTRHIPERVVCSECRRPMEKFLMYRCCTD

**16419389_peptide|Cpapaya|evm.TU.supercontig_4.9|evm.model.supercontig_4.9**

MYKEIVTKRDVKLRHLRSTGDVPAPSPSAVRYLVHQPSTIVRDVTVTADDSLLPMAIMAAHSPDPQQHLDVKPVFFYIVQDILLRGIPDAPKNLIVNMEALKEKNGNAVLEASADIIEAICCEMVCKCSGGHDAGATTKAIFDILAIYSWEAKVVLGMAAFAVIYGEFWLLSELYATNSLAKSLALLKQLPIVYEHSIWVKPVLDELNNLIKAMIRNVNYIITISEKSPQNISITTANSSNLPLIPMAAYWTMRSIVACASKVASLSGPGVDEHITDISELSSLVMRLHEYTVRFEELRDEEAYIGLVQIFRISPQSNITIIEALINPYNTKDIDHQNALMDCSTKKMVQIDVLRGKHVVLLISGLDVATTEEFEVLKQQYKESRGKVEYQYEVVWIPIVDTSVPYWPGDFEKLRATMPWLALHPTHIKAPAIKYIKNVWQFTKKPVAVTMDAQGKVVCKNSLHMFWIWGNKAFPFSDQIEETLWQKESWKLDLFIYGITDVEIHRWRTEKRFICLYGGEDIKWITEFTNAVRAFANTVNLSHELIYVGKRNSPRAKQVAAIIVEKQLSQCWPGLLKDSGCSFWSRLESILYSKLKHFKASDKYDILMKEAMAILGFDGSDNEWAIFSEGASGMARAKGDMALHCMTNYHAWYNHETCKDSFMTALNTYLLKQHSSKHCYRLMFPEVNWGVPDKMVCFECGRLMEKYIMYRCCDD

**16804180_peptide|Rcommunis|29633.t000039|29633.m000920**

MAFRSLSAASTGTAAQQLIRSDRSMFALSDENSMMKQIQATHAPDGREFDVRPLLNIVADILSRANIPHADTALTASQTHAEMEDKNRQANFIAMLEALAHVIDRIACEISYKALSGSDPHATTMSLLNMLSSYNWDAKLVLTMSAFALNYGEFWLLAQIYSSNPLAKSMATLKQLPYILEHTVPLKPRFDALNKLIGVMMDVTNCVVELRELPTAYISAETGALTTAIAHVPTAAYWTIRSILACASQITSLTTLGHEFATTEVWELSTLAHKLQNIDDHLRKQLSLCHQHIDERRNVESYQMLLNLFDMIHIDNMKILKALIYPKDDIQPLVDGSTKRRVNIDVLRRKNVLLLISGLNISHDQLSILEQIYNESRIHATRMDSHQYEVVWIPVVDRTVQWTDPMQKQFEALQATMPWYTVYSPTLIDKVVIRFIKEVWHFRNKPILVVLDPQGKVACPNALHMMWIWGSTAFPFTTFREESLWREETWRLELLVDGIDSTILTWIKEEKYILLYGGDDVEWVRKFTNTARAVSQAARIPLEMVYAGKSSKRDKVQSIIAAIPVEKLSQYWDPTMIWFFWTRVESMLFSKIQLGKIDETDPMMQEIKKLLSYDKEGGWAVLSRGSNVVVNGYSSTMLLTMIEYDLWKDKVPVNGFDLSFKEHHNKLHDLAHPCCRLEFHSTTGRIPERLKCPECLRSMEKYITFRCCHEEEAAEILY

**16804181_peptide|Rcommunis|29633.t000040|29633.m000921**

MAYAASTSASQHLIKSEGSMAALSDEDSLMKLVQETHAPDDRKFDVRTLLNVLEDILINCESREIESIMPATQTHKETPDNSREVLESLSYIIDKLSSEISYKVLSGADGHRTTISFLNMLSNYSWDSKLVLIMLAFALNYGEFWLIAEIRFSNPFAKTMATLKQFRPFILEYAATSLKPTFDALNNLIRVMREVTKCVVEVGELSSEIPAYLELSALVQRATYWTTISAMACATQINTLAKLDNADQLAGELSTLADKLQNIHDRLRSQLTICYQQKDDMSYQMLLNLFKSVHIDNMKILKALICSKNDIQPLFDGYTKKRVNIDVLRQKNVLLLISDLHIPDYEIFFLETHYRITGNHLFEVVWIPIMDRTIKWNDLGQKQFESLQSKMPWYTVYHPTQIDKVVIKFIKEVWHFNNKPILVVLDPHGKVVSPNALHMMWIWGSHAFPFTSLREEMLWKEETWRLELLVDGIDPMLVNWVGEEEYIFLHGGDDVEWVKEFTEMVRKVSQAAQKPVEMVYLGKSYKKDKVRKIAKTITDEKLGHSWDPTMIWFFWTRLDSMLFSKIQLRKIDENDTLTHEIKKLISYDKEMGWALLSKGPNIVVNGHSTTVLRALTEYDKWKENVPVKGFDLSFKEHHDKLQNTNGPCCRFEFPSTFGEIPEHLKCPECLRSMEKHMAFRCCHDC

**16804182_peptide|Rcommunis|29633.t000041|29633.m000922**

MVMWLCQYLQSTINAEAEAEGQKEEKALVTKLSFEAESEAERSKEHKALVAPLPSDIDRISSEICYSTQINGADVDATTLSLFNMLAKYSWDAKLVLTMAAFALNYAKFFLLLRLYPSTNRTIIKTLATIKGLPFIFEYTNESIKCRSDEIDKLIQAMMDATRSVVKFRKLPPVYISLEASALSTALAHIPTVVYLIIRSIVACSTEFASFTNVALGTVRELSELTEKLVQRCNVLKQQLEICQEHIEKKRNVEAYLKLLNCFDTANKDNIESLKAFIKAKDGDLPLFNGATKKEVDINVLRRKNVLLLISGLDISQDELWILKLIFREANIIATRHERQYEVVWVPITNHSVQRTDLMENEIIKNLKYTMPWYSVQNPTLIDKVVIKLIKEVWHFRNNTVLVALDSQGRVVSPYALHLMWIWGSHAFPFTRSRQESLWKDETWRLELLVDGLDATILRWAFEEKHIFIFGGDDVEWVKTFTATAREVAHAARFQLELVYVGNRSKRDKIKQIIDSIEKDKLNTYFWHDLTAIWYFWTRLESMLFCKIQLGNKFEENDGIMQELKKLLSYEKEGRWAMLTRGSNIMVNGAGAKVLHALTEYDPLNDLNSPNQDFGLSFKDHYNKINTGTSVHSCCRFSFPTAARRFPKRTTCPECHRIMAKQIVLSCCHKGISEIPF

**16804185_peptide|Rcommunis|29633.t000044|29633.m000925**

MANQTLANMQQLMNKNQPNVQQPLPNQAQVNLHQLMNPTPSNMQQTPATMQQLTNPAPANMQQLINPTPAHMHHHINSNPANTQQLIKQTPFTMQQLLKQTPASAHQLIKADRLLFSSSDDNAMTKQIQATHSPDGREFDVKPLLNIVEDIFDRAAPAIESLALPAAAHHARNEALDDNTYHSSVMAMLESLSFVIDRVASEITYKCSSGGEAHAITMSILNTLSSYTWDAKLVIALAAFAMTYGEFWLVAQNYTSNQLAKSMAILKHMPDILEHSSMLKPRFDSVKNLITVMLAIAKCIVEFQELPPQYITMDVPALSAAMAHLPISVYWTIRSIVACASQVIGLIGLGHEHVASTTEAWELSSLAHKLSNMQSHLQNQLGLCYKHIDERKHMEIYQNLIRLFEMAHIDNMRVLKALIYSKDDIQPLLEGTTKRRVNIDVLRRKNVLLLISDLDITQDEISILEQIYNESRLHPSRQESQYEIVWLPILDQAVPFNDNMLKKFEALQSVMTWYSIHHPSLIDRAVIKFVKEKWNFGKKPILVVLDPQGRVACPNAVHMMWIWGSLAFPFTTIREEALWKEESWRLELLVDGIDPIITNWIEEGRYICLYGGEDMEWIRKFTSTARAVAQAAGIPLGMVYVGKSNPKERVRRNIATIMVEKLSHYWQDLTSIWYFWVRIESMWRSKNQLGKTSENDSLMKEIMAMLSFDSSEGGWAIFTGGTDEIVKAKGSIFLTCLSEYTSWKDQIQQKGFLPSLKDYLKGLHTDHHCNRLILPGSAGTIPERIVCSDCSRNMERYIMYKCCDE

**16804186_peptide|Rcommunis|29633.t000045|29633.m000926**

MANQPSVQQPLTNQTQANLQQLMNPTPANTQQLNNPTPSTLQQLANLTPANMQQLINSTPARMQHHPINPTPTNMHHPINSTPTNMQQPTKQTPTTMQQQLKQSPASAHQLIKGDRLLFSSSDENAMTKQIQATHSPDGREFDVKPLLNIVEDIFERAAPTIESLALPAAGHQARPEVLDDKIYHSSSMAMLESLSFIIEQIASEISYKCSGGGDAHATTMSILNMLSSYTWDAKLVIALAAFSMTYGEFWLVAQNCTSNQLAKSVAILKQLPDILEHSSMLKPRFDSVKSLIKVMLAIAKCIVEFQDLPPRYITMDVPALSTAMAHLPISVYWTIRSIVACASQIIGLIGLAHEHIASTTEAWELSSLAHKLSNMQSHLQNQLGLCYKHIDERKHMETYQNLIHLFDMAHIDNMRVLKALIYSKDDIQPLLEGTTKRRVVNIDVLRRKNVLLLISDLDITQDEISILEQIYNESRLYPSKQESQYEIVWLPILDPAVPFNDIMLKKFEALQSVMTWYSIHHPSLIDRAVITFVKEKWNFGKKPILVVLDPQGRVACPNAVHMMWIWGSLAFPFTTIREEALWKEESWRLELLVAGIDPIITNWIEEGRYICLYGGEDMEWIRKFTSTARAVAQAAGIPLGMVYVGKSNPKERVRRNVSTIMVEKLSHYWQDLTSIWYFWVRIESMWRSKNQLGKTSENDSLMKEIMAMLSFDSSEGGWAIFTKGTEELVKAKGSISLTCLSDYTIWKDQIQQKGFLPSLKDYLKSLHTEHHCNRLILPSSAGMIPEKIVCTECSRNMERYILYKCCDE

**16804187_peptide|Rcommunis|29633.t000046|29633.m000927**

MANQTPTDMQQLKNQNQLSVQQPPPNQAQANQQQLMNPAPANTQQLTNPALTTMHQLPSAKMQQLTNPAPTTMHQLPSAKIQQLIKQTPASAHQLIKGDRLLFSSSDENAMTKQIQATHSPDGREFDVKPLLNIVENIFDRAAPTIESLALPAAAHQARPDALDDKTYHSSFMAMLESLSFVIDRVASEITYKCSSGGEAHAITMSILNTLSSYTWDAKLVLALAAFAMTYGNFWLVAQNYTLNQLAKSMAILKHMPDILEHSSMLKPRFDSIKHLIMVMLAIAKCIVEFQELPPQYITIDVPALSAAIAHLPISVYWTIRSIVACASQITGLIGLGHEHIASTTEAWELSSLAHKLSNMQSHLQNQLGLCYKHIDERKHMETYQNLLRLFEMAHIDNMRVLKALIYSKDDIQPLLEGTTKRRVNIDVLRRKNVLLLISDLDITQDEISILEQIYNESRLHPSKQESRYEIVWLPIRDPAVPFNDNMLKKFQALQSGMTWYSIYHPSLIDRAVIKFIKEEWNFGKKPILVVLDPQGRVACPNALHMMWIWGSVAFPFTTIREEALWKEESWRLEILVDGIDPIITNWIDEGRYVCLYGGEDMEWIRNFTNTARAVAQASGIPLGMVYVGKSNPKERVRRNVSTIMVEKLSHYWQDLTSIWYFWVRIESMWRSKNQLGKNSENDLVMKEIMSMLSFDSSEGGWAIFSRMADEVVKAKGNIFLTCLSDYTVWKDQIQQKGFLPSVKDYLKGLHTEHHCNRLILPSSAGMIPEKIVCTDCGLNMERYILYKCCDE

**16814026_peptide|Rcommunis|29929.t000291|29929.m004788**

MAVVPHRSNPRGERHMFSTSDDNAMMKQIQATHAPDGREFDVRPLLNVVEDVFQRAVPPSGLATIVQPQEKTLQNGFYEMLDLLSYTINKISCEIACKCSGGGDAHATTLAIFNLVSSYSWDAKLVLALAAFAVNYGEFWLVAHLYLTNPLAKAVALLKQLPDILERADALKPKFEAVSSLIRAALDVAKCIVEFKELPPQYITPDAPEMLIATAHIPTAVYWTIRSIVACATQIIGLIGMGHEYMASTTEAWELSSLAHKVRSIHEHLMRQLTLCYHHIDEKRHVEAYQTLIRLFDTIHIDNIKILRALIYAKDDQLPLYDGHNKKRASLDVLRRKNVLLYISDLDLPHEELSMLEQMYSEARQNPARTESHYEVVWLPVVERSTAWNDAKQKQFENLQSVMPWYTVYHPSLLDPAVIRYIKEFWKFNKKPLLVVLDPQGKVVNPNAIHMMWIWGSAAFPFTSVREEALWRAENWKIDLLADTIDPIIHSWIQQGKYICLYGGEDIEWIRKFTMTANALAQAAGIDLEMLYVGKSNPREKVRKNNIIIQNEKLSHVLQDLTLIWFFWVRLESMWHSKVQHNRTVENDIIMQEIVTMLSFDGSDQGWAVISKGSGAENRQLAKAKGSDILNCFDDYQSWREIAEEEGFVPAILDYLHGHHNPLHCNRLILPGTTGSIPEKVVCAECSRPMEKFIMYRCCTD

**16964846_peptide|Csativus|Cucsa.156170|Cucsa.156170.1**

MDSGNKFSLASRHQLAKGNKFLSAISDDNVMMKQILATHDPDDRDVDTRSLLRLVENILKRATLAADATGSYEQLESLEETGTHQAGLTTMLEALSYTIDRISSEISYKALEGIDPHATTLAIFNMLASYRWDAKLVLTLAAFALNYGEFWLLAQIYSQNQLAKAMAILKQLPGIFEHSIALKPKFDALKELVAAILDVTWCIIDLKELPSAYISQEVPAMSTAVAHIPTAVYWTIRSIVSSATQITSLTSMGYELALSTSTDAWELSTLAHKLKNICDHLKKKLVLCHQHIEEKKDIESFQMLINLFEMNHLDNMKVLKALIYPKDDLQPLVDGSTGQRVNLDVLKRKNVLLLISDLNISHDELSILDQLYNESRAQGMRVESQFEVVWIPIVDHSIKWNDSMQKRFEYLLSIMPWHIVHHPTLISKAVTRFIGEVWQFRNRPILVVLDPQGKVVSPNAIHMMWIWGSLAFPFTSVKEEVLWKEETWRLELLVDGIDPAVLNWIKEERYIFLYGGDDIEWIRKFTTTAKTVAQAARIPLEMVYVGKSSKRERVKKIITTITTEKLGYCWQDLTMIWFFWTRIESMLYSKIQLGKADDCDPLMQEIKKLLSYDKEGGWAVLSKGSNVILNGHSTTMLPTLGSFDSWKQEATDKGFDIAFKNHHDELQGITHPCCRFEFPHTSGRIPENFKCPGCDRQMEKLTTFLCCHDENSNE

**16968247_peptide|Csativus|Cucsa.196320|Cucsa.196320.1**

FGRPALKMHQLTKTDRRMLPVSDDNAMTKQILATHSPDSHKVDVKPILLIVEEVIRHATPDIIAKGNGQLDDQLGLAEMDGMLEPLAHVVQKVGAELACKCSGGDAHATTMAILNLLSNYSWDAKVVITLAAFSVTYGQYWLLAQLYTTNMLAKALALLKQLPDVIEHSNSLKPHFDALSKLIAAILNVTKCIVKFTELPSQYISSDTPAMSVALASFPTAAYWTIKSLVACTSLIESLVHLEILKRKHVLLLISDLDIPHEEVMILDNLFKEAHQRPEIRYEIVWIPIIDPAIEQHSKSKHKFEELKQLMPWFSVYDPSIIELSTIRFIKEKWNFRKKTILVALDPQGKVSSTNALHMIWIWGNLAFPFTSEREEELWKTESWRLELLIDGIDFSILDWAAEGRYICIYGGEDTEWIKEFTSKTKKVAETANVDLQMAYVGKNNAKERVRKISIMISDNKLSHYWADSTLVWFFWARLESMMYSKLNYGKTVENDPIMQEIMTLLSFDGSDKGWAIFFGRAGETTRAKGETVLSCILAFDQWKEEVEEKGFVKALADYLQQLKTPHHCNRLILPGLAGNIPENVVCAECGRAMEKYLMYRCCVE

**16983228_peptide|Csativus|Cucsa.394600.1|Cucsa.394600.1**

MAVAAPRKLSLIKPDRQLFAGGDENALTKQVLATHSEEPLEFPVTPLLSLVEQIFLRAKLNTLQAYTCALISGTTRAQLEAIEDKSPSPTDLLDLLDFVSFTINRVSNEIQYKCSGAGDPHTVTMEVFNLLSSWPWDAKVVLALAAFAINYGEFWLLVQQSSTDLLAKDISLLKKLPEIFERVDIVKQKFEALDKLIKSLVDVAKCIVDFKMLPPHYITPDTPEMKSATTLIPTAIYWTIRSIVACAAQNAGLIGVGHEYLASASETWELSSLAHKIDNIRKHLEQLLLACHHYINEKMHHEAYMNLVRLFEIPHIDNNKILRALIYSKDDKPPLLDGLSKEKATLEVLRKKNVLLLISDLDLSIVELSMLDQIYRESRQNKTRSESDYEVVWMPIVESPWTEDKQVKFEALLGLMPWYSVAHPSLIESAVIKYVRQVWNFIKKPLLVVLDPQGKVVNTNAVHMLWIWGSLAYPFTSAREESLWKEETWRLELLVDSVEPLIFQWMEAGKYICILGGEDLAWIRGFSAKALGVAKDAGINLEILYVGKSNPGEKIKKNIAGILADKMIRTLVDPTLIWFFWVRLESMWYSKTQRGNTIEDDPVMQETMTMLSFDSGDQGWALFCKGSTDILRAKAETITNVVDGYEERWKVHVKEEGFIPAMTKDLQDIHTPEHCNRLILPSSNGTIPEKVVCSECGSAMEKFIMYRCCND

**17640596_peptide|Ppersica|ppa001579m.g|ppa001579m**

MALAPQNFNRAPAPPQIYNTPVPTAQNFNTTPQPAAQNYSTAPVPAAQNYNPALAPAAQSYDPALAPAAQSYNPAFAPAAQNYNPAFAPAAQNYYKPPTTTRRGGDSYNRPGRRQFSATSDDSALTNQILATDRSRDRPYDVKSIPLKHILQTVDVILARVTKPDIHGAFLVPIFGKWLSGEDPNKTTMDILDIVQHHDWDEKVVLVLGAFAVKDGEFWLVAQLYTTNPLAKAIGQLKQVQEILERAGTTLKPKFESYNNLVRAIINVTKCVVQLHDLQRDPHVTTEHESAATTAHIPTAVYWTIRSIVVAASQLLGITGMGPDDKFIVFHFYLASIRYLTETWELSSLAHKLENIHSHLQENLDRLYEIIKRKKDDEALAAIAYILETPHIDNVKPLRVLFYKDDLPALYDCYNKKRVDIDVLKRKTVILFISDLDVVNENEYMIVQNMYMEKRHSPVRPESQYEVVWVPIVDTWTDAKYQQFEELRRNMEWYTVFHPSVVSPTVIRYIRKKDKWNFQKKPLLVVMDPQGKIVHTNAVHMMCIWGSVAFPFTSSKEKLLWEEETWRMELLADSLDQNLINWIAEGKYICLYGGEDIEWIRNFTRAAKKVAAESGIQLELLYVGKSKPKEKVVKNIMTTIQFEKLSHTLEWNLIWFFWVRLESMWQSRGQQLQTEALRSGRLTDSLKSDLVMQGIISMLSFGSSDRGWAVIGTGSAGMSKANGEHMFRSLNEFNLWNRRVNEIGFVPALNEYLDGVYKQAPHHCTNLILPATGLMPETVACAECGRLMERFTMFRCCTD

**17641626_peptide|Ppersica|ppa026573m.g|ppa026573m**

MDVLTFKKPVSQVDSNNSVENAEAADQKSLFARTDTEILEGIYATHVVCHEHDALNVDSLFSITESILKCSKQIVDHIEQMAPDEKNSHDATLKILSKVPKYSWEAKAVLALASFSLEYGEFWLTAQHQRSDQLAKSVACLKGVPVLLKPENLKQRGKSVAQLNNVIMSTLEVIDCILELEKLSSIYNEVKELREILTSARKDISVNVYWCIITTVACGTNFTLLTSDEGKSHDLVQYAQKITIILKNLKGQLKSCNKEIEKLQIYMKLKQLFQIPTEIMEAIKTLIFFKDDMKTTIFDGFTKKLVQIDILRTKNVLLFISSLNISDGYISLLEPIHELTKKNDEYKIVWIPIAEEWHEQQQIKFQTLRDKMPWYTINQTNAHISGIKYVKEDWNFKGKPMLVVLNKKSQLQHSNALHMISMWGCKALPFTQKKQEELLLSLHDTWFAEVVGEIHTSVSKWMKEEKYIFFYGGDSVWTNEFKEKAIKVINGDIIEKSNISIELYHVKKGANNSGMVDKFSTFWAAIETMFRIKVSNKQLDHVAQDVQKLLSLKNDKSGWAVLLHGHKVVTIGAGSTISKALEHCDTWNQHETTITVETLGNCFNAEHEKEKKKEFEQTGHVCTCFDISSAAGSTLEDMICYECGSLMETFISYKCHHVKKNDESRQILPAWTKAWNLTISEASAKLKQLMFGDGPVPLHMILDALSQFGSDYGRAKINSSWV

**17644790_peptide|Ppersica|ppa023840m.g|ppa023840m**

MGSVSTSVCQAVSTTTTTTTTSIENMEASKKNLFAMSDAEILEVIYATHEVSHDHDSFDVHSLFSITQSIIKCSKQIVDSIDQKVCMVLLYITPFSTPLCVLKSIVREVPCKAPGEKKAHDATLKILSKVSKYSWEAKAVLSLAAFSLEYGEFWLSAQHQQSEQLAKSVAFLKGVPILLKPENLKKRGRAVTDLNNLIMSTLEVIDCIFQLEKLSITCNDVKELREILANARKDISVNVYWCIITTVACATNVTLLTSDEGNSHDLVQYSQKITIILNKLKQQLRICKEEKEKLRTYMKIKPQIEIPTEIVEVMKILIFFKHNAETTIFDGSIKQLVHIDILRRKNVLLFISSLEISEDYIARLRPIYDFTKDKNEYKIVWIPIVEKWTKDLQHKFETLRAKMPWYTVGQAGAHIAGIKYIKEDWNFNGKPMLVVLNTMSQLQHFNALRMIWIWGCQAFPFTQEKEEQLLLSLQDTWFSAIMDGIDTEISKWNKDDYIFFYGGDSERVNQFKEKATALINDEIKKESKISIKLYPVEKNANNDGRDDSFSTFWSAIENMFHIKVINKQVDDVVKQVQKLLFYKDDKSGWAVLIQGRRLVTIGGSTMYTVLEQYHTWNQKVTLTVENFGQVFNQEHGTAVAEGPGHVCSCFSIPSATGSTLEAMVCYECGNSMETFFSYKCCHVKKKAPLITY

**17650601_peptide|Ppersica|ppa015869m.g|ppa015869m**

MSDKKILDQIYATHYVNVDTSFDEDSLFEIVENILKHAIQTVDKIVQGTQVHEENIEEKPLKANFSTPLCILKSIASEMQCKPPGEKVAHETALAILNKLSNYSWEAKAVLTLAAFSMEYGEFWLLAQAQESDRLAKSISILKRVPFLLKPSNLQKRRQAVLELNNLIKVTMRVIGIFDQFEKLSSYDPKDVPELALAMEHIPVDAYWAILTLVACATKVTILTSDEDKEHDLVPYAQKIHFILNKLNMQLKICRKQVEDAEAYRRIRKIFRTPTEIKEVFKALIFSKDNVQPLIDGSTKQTVDIDILRKKNILLFLSSLDITDDDISILKPIYEFTKKEDQHKIVWIPIVEQWTDELRKKFETLRIMMPWYTVQISAPIAGFRFIKEEWNFKGKPTLVVMSPQGKVEHYNAFHMIRVWGPKAFPFTEATEKEISKSREWFGNLIREIYPTPPDSKEDEYIFFYGGKDKDWMKQFKEKATALANDLILKEAKINIKLFCVGKDSKGEDDFGILWRFWTGIESLFHTKINKQADSATLEIQKLLSYKNESGWAVLSKGSSLVVAGHGISILKVIEDFDKWKGQVREKGFEFCFTTYHAKIRLTPCCRLDIPGSTGKVPETMNCPDCNRSMETFISYKCCHIDGPNVHH

**17652314_peptide|Ppersica|ppa023699m.g|ppa023699m**

MRDKRTSPQSDDSALLRQIRETHSPGRSHSVDVNQILQVIEEIFHRATHSTAVVLAGTRELADTVEDRTTLPSVDVLFHGLSYLIQKIYCEISCQCSGGGDVHATTMELLRTLSNYSWEAKVVLTLAAFAVYHGEFWLVAQLCTTDPLAKSVAILKQLSDMVEHAASVKPQIEAIDNLITAVTNVTKSIVVCSEMVKLQSHYISEDTPPLSIALAHIPAAAYWVIRGILASASHIAILAGSRHEYIASTTEVWELSSLAHKLKNIHDHLTSELENCRQYIVAKRYDEDYETLRRLFQGLHLDNLKNLRALISHKDDAQPLQIGTTRQYSLEVLRRRHVLLLITDLSLSNEEIVILDHIYKQQQNRAEVEYGIVWLPVVDATTWDEAKRFRFEELKSKMPWYAVHDPQIIEPPVIKFIRNDWHFDKKMIIVSLDPQGRVSSLNAVHMLWVWGNVAFPFTDEKEQVLWNAESWRLQLVANGIDPIILDWIEKGKYICLYGGDDLEWIRKFTVRAKVVAGLAGISLELLYVGRSTATRERIRKVNKVIETENLSRFWPDYTSNWFFWSRMDSMRCSKAKHHRTVENDEILKEVMTLLSYDGSDQGWVMVWRGSNETARANGQLTLHTLDEFEAWKNKAAESGFVPTLSDELKRRHKPQHCTRLIIPGFGPDIPDRVECAECGSEMEKFFMFRCCTD

**17653328_peptide|Ppersica|ppb018052m.g|ppb018052m**

MRREIPTVGMFLIMNSIQLNEYSHYGFTLTEPEEILEIICENHAPENHPKFDVRSLFFITENIIKHSTHTSIVSKSDEIKPQNFDNFISPLCALKSIGCEMSCKAVRENSAQTTTLGIFGKLTKYSWEAKAVLALAAFAMEYGNSWLLSQLYPQSDQLTTALAILNRVPLLLNSTANFKKQQETVVELNKTINATLQVIKCILKLDELSAHIDPNHASLKSAKKDVPINTYWSILTIVSCATEDNAYAKPATLSVGYTNKEVFDIDVLDGKYITFYISTLDNVSHKDILSLKEVYEGTENNKCKIVWIPIVEDWTEYGREEQFMEWRSKMPWYAVQYLSPATIKYIKEEWYFENKPLSVLMNPHGDVENLNALNWIRIHGINFFAFRNINVKTWIAPVVQELMTPALDTWMKEGEYIFFYGGTDDYSMERFRMKANCAKVSILEELKIHVKLFCVGKLEKGRSFSDESNAGGFWSSIQSLLSTLSDYKLHEEHTALRKQVHKLLSYKNDESGWCVLSKGSSVVTSGHGWAISKVLDEFDQWKQQISHERSFGTCFQVYHEKVLAQTTTAGAHSAGCSFGNARNMECCPVCKTPIEATLVSYKCCRMCNLTPY

**17653917_peptide|Ppersica|ppa002007m.g|ppa002007m**

MALVPQSKFSAGSRGDNRARAPALRDGRRQFSSTSDDSALVNQILLTDKSHDRPYEVSSLAVKNILQTIEAILSRGTPVPAATHADALEHEKALRASLSTLSESFDVPNHVFNAISCEILCKSLAGEDANKTTMDILDIVQNYDWDEKVVLALGAFAVKDGEFWLVAQLYTSNPLAKAVGQLKQLQEILERAGTVLKPKFDGYNNLVKAVLKVTKSIIQLQELQNDPHLNPEIKSAASTAHIPTAVYWTIRSIVVAASQLLGFTSSEPEYVTEAWELSSLAHKLENIFNHLQENLNKLHQIIQKIKDEDAFNAIARILESPHIDNSKPLRVLFYKDDQPALYDGLNKKRVDVDVLKRKVVILFISDLDVVLGNEYMIVQNMYMEKRQNLGRPESQFEIVWVPITDEWTAAKYQQFENLRDNMEWYSVFHPSVVSPIVVRYIRDQRKWNFVKKPLLVVMDPQGKIVHQNAIHMMCIWGSLAYPFTSTKERLLWDEETWRIELLADHLHPNLFTWITDRKYVCLYGGEDIEWIRNFTKSAKSVALEAGIALELLYVGRSKPKEKVVKQILSIIQTENLSHTLEWNTTIWYFWVRLESMWQSKGQLLSEQSTTHFKTDNLKNDPIMQGIISMLSFGSSDRGWAVIGIPSADMAKANGDHMLKSMKEFTDWKIRAADAGFIPALNEHLEGVFKQAPHHCTNLILPATGIMPETVACAECGRLMERFSMFRCCTD

**17655449_peptide|Ppersica|ppa026484m.g|ppa026484m**

MTSLPKNKARNATVESSTVTHEEHIRVERVESYVSEDQIWTVVHENHFPDQNKLSFNVRDLLSVTGNIIDDAANNSIVDNILQQHTAPDMTIYDADDTFISPLYLLKSISCQDGEQVSLPLRTEAIFKKLKTFSWEAKAVLTLAAFALEYCDKLTKLVAILKRVPILIKHETLKKRRAAIAELNNLIMETYHVIGYIVNLDDLLHNNNPNDVPTLTTAGRKIPTVVYWTIFTIVACTDEINRITSVKYNDEPDNLPNLYLEKIKEIVKELKEQYDRCMKEKGYQHLDSLQRYCQSAALYKMINLIDGNAFINEVKGKYVLFYISSLENISKELLRLTNLYKIIDKEYKCKIVWIPIDGDWTTEAEKKELQFMEWRKMMPWYAVQYFPSASYMYLKKEWKVRENSTAVLINPQGKVENTNALTLIKEFGIDFFAFLDIQIHTMLKPVVEHIIRDDSVLKQSMKNQGYNFFIGGKNQKTTIDLFEKITEAKDAIETELKMKIGLARVLEKTETAKTFWARMKNLFFSLARYSKEYEYEQVTKEVHKLLSYKLHTDDMDGWIKLTKGWTVVTCGQANTIYTTLEKFSVWKQHINDFGDAFTKYHDSLIT

**17659164_peptide|Ppersica|ppa002624m.g|ppa002624m**

MSDQKILEQIYGTHVHADESFDDDSLFVIVENILKRATQIVDKIVQGTQVHVENIEEKTPKPGFSTPLCTLKSIASEMQCKPPGEEVAHSTTLAILNKLSSYSWEAKAVLTLAAFAMEYGEFWLLAQLRESDRLAKSIAILKRVPVLLKHSNQHKRRQAVLELNNLIKATLQVIECIDQFDKLSSYDPKDVPGLAIAMDHIPVDVYWAIMTVVACATKVTILTSDEVKDHDLAPFAQKIHYVLNKLKIQLIVCRKQIEEAEAYRRIRKTFQTPTEIMEVFKALIFTKDNVQPLIDGSTKQMVKIDILRRKNVLLFISSLDISDDDISILKPIYEFTKKENQHKIVWVPVVEHWTDDLRKKFETLRLKMPWYTVQNPAPIAGIRFIKEEWNFKGKPALVVMNPQGKVEHSNALHMIRIWGVKAFPFTKATEEELSHSHRDRWVGNVVHDIHPSLPNWIKEEKYIFFYGGKDNDWIQQFTKKASALANDPVFKEAKIHIELFCVGKGSKGEDDHGILGHFWSGIESLFFTKGHKQVDPVTQEIQKLLSYKNESGWAVLSKGSSVLLTGHGVSILRVIEDFDKWKDHVKEKGFEFCFKAYHEKVRLATRPCCRLDIPGSTGKVPDTMKCPDCHRSMETFISYKCCHIDGPNAHH

**17661991_peptide|Ppersica|ppa002363m.g|ppa002363m**

MLGIAQNAVSKVTSAVTNTAHHIEGELSLFTMSDNKILELIYATHVHEDDSFDVDSLFLVTENIIKRSTQIVDSIVQGTQVHVDTIDEKPPKASFSSPLCTLKSIGCEMSCKPPGEEISHKSTLAILNKVSNYSWESKAVLALAAFALEYGEFWLLAKIHQSDLLAKSVAILKRVPVLLKPADLQKRRQAVVELNVLIKTTLQVIECIFELEKLSTYDPKDVPALAIAMDHIPVDVYWSIITIVACATKITLLTSDEEKHYDLSQFAQKIHYILNKLKIQLLICKKQIEEAETYRKLRKLFQTPAEVMEVFKALIFTKDTVQPIIDGSTNKTVNIDVLRRKYVLLFISTLDISDDDISILKPVYEGTKKEDKYKIVWIPIVEHWTDELRKKFELLRAKMPWYTVQYFAPVAGIRFIKEEWHFKGKPAVVVMNPQGKVENTNALHLIRIHGMKAFPFHKGIEDKITNDREWITPIVNDIHPSIQTWIKEEKFIFFYGGKDNDWIQQFTKKATIIANDPSIKDLKINIELFCVGKSPKGGEDLGVLGRFWNGIESLFFTNVNKQTDTVTKEIQKLLSYKNESGWAVLSKGSTVVVSGHGFTILKVLDDFDTWKTFIKEKGFELAFKAHYEKVIQTMKHCSRLDIPTVAGKVPETMKCPECPRTMETYVSYKCCHTDGPINAHH

**17665564_peptide|Ppersica|ppa002391m.g|ppa002391m**

MLGLANNVVSKVASVVTGHHELSLFSMSDKKILDEIYATHHVNVDTSFDDDSLFGIVENILKHATQTVDKIVQGTQVHVENIEEHTPKASFSTPLCTLKSIASEMQCKPPSEEVAHKTTLAILNKLSSYSWEAKAVLTLAAFAMEYGEFWLLAQLQESHRLAKSISILKRVPFLLKPSNLQKRRQAVLELNNLIKTTLQVIEIFDQFDKLSSYDPKDVPELASAMDHIPVDVYWATVTVVACATKVTILTSDVEKEHDLAPYAQKIHFVLNKLKIQLKICRTQIEEAETYRKLKKTFRTPTEVKEVFKALIFTKDNVQPLIDGSTKQTVEIDILRKKNILLFISSLEISDDDISILKPIYESTKKDNQHKIVWVPIVEQWTDDLRKKFETLRLKMPWYTVQNPATIAGIRFIKEEWNFKGKPTLVVMNPQGKVEHSNAFHMIRVWGTQAFPFTETTEKELSNSHGHKWVGNVVKEIHPTLPNMMKDDKYVFFYGGKDNEWINQFTKKATAFVNDPIFKEAKIHIELFCVGKGSKGEDDHGILGRFWTGIESLFHTKIHKEPDSVSQEIQKLLSYKNESGWAVLSKGHSLVVTGHGVSILKVIEDFDKWKDHVKEKGFEFCFTTYHEKIRVANRPCCRLDIPGSTGKVPETMKCPDCHRSMETFISYKCCHIDGPNAHH

**17669983_peptide|Mguttatus_v1.1|mgv1a002070m.g|mgv1a002070m**

MASGVSMPAARKQQSMIGGNDRRPMLSDDNALRKQIQATHDYDGRLFDLESILIIVNDILNLVSPGIDGILNGSGKHAVIREETAALTGFDGIHDTLAFLLNKVSCECSGGGGGDGHASSIEILNSLSSYTWEAKAAIALASFSVNYAQFWLVANRFTTDPLAKSVALLKQLPDIINLSDHVIKSRFDTINNLVKDLLELTTCIAKFSRLMPSKYISEDVEPMAVAIAHFPVAVYWIVRILVACASQVTEIIGLNRQVFSSTAETWELSSLGHKVSKIHDHLKTQLGLCYQYIDEKKNTDYFQTLVRLFETTPHFDNQRILGQLIYLKDDLQPLVIGANKNIKVGVEALKGKTVLLLVSDLDISQDELRILSHIYQESKTRPDFHYEIVWVPVVERTIKRNEEHELKFEQLQGKMPWYTLHHHSLLEPAVVRYIKEFWQFTKKPILVSLDPQGKVASPNALHMVWIWGNLAYPFATRNESILWEGEKWRLKLVVDGIDKSILTWINEGKVICLYGGDKIEWIRDFIRTARNAASSAGIGLEMVYIGKNATKERMKRLNETVTSNGLSHCWTDPTSIWYFWTRIESMMHSKYSHNKGAKIDDDGDVDHIRREVLTMLTFGGDGDRGWAMFSQGSGAGPGEVARAKGDAMLSGLKDFETWAPEATRIGFVAALNDYLAGNMSGEHCNRLVLSGVDDVPETVVCAECRKPMEKYFMYRCCDD

**17684402_peptide|Mguttatus_v1.1|mgv1a001374m.g|mgv1a001374m**

MANRNLLPPSNPLPLTTAERVVAPTRAPLQSGVHDFVTDHSLIRPASHHDQVVDHHMGATTANHHLGQQVAVPLSAPVLGRRQPGRQGDHDRSNFFSSDDNALTKQIQGTHAPDVQELDVKPLLAIVEDIMRLATPASHDTNLIPGGQIVASSQHVEKLDDKAFHNTTASGHDNNKVYDNSKIYDNSKVVYESKVYGSTYDHDADIVRVLAYPINKISCEIVCKSSAGGESHTVTMHLLNTLSNYSWAAKVVITFAAFAINYGEFWLVEHLQAKDPLAKNIATLKDLPDVMAHAGALQQKFDQVLNLLNQVLKVTHRIIEFKELPDVYISHDSPEYMAATAHIPLAVYWIIRSLLVCASTLLNLIGSGHEYITSTAESWEISSLAHKLSIILEHLQTQFKNCRDFVEKKKEEDEYKRFVIIMTSAHVDNMKVLRAMFRSREDQRPLYNGRTKTNERLDVLRSKYVLLLISDLELPLEELHILNSIHRQQSDRHEYEVLWLPVIHDPSTTTAAAAAMTPMQEKAFQDLRNVMPWYSVEHPSLVERVAIRYIREYWKFVHMPMLVVLDPHGKPSNHDALPMIWSWGTEAFPFTGEHEKYLWASKIWNITLLADNIDARIPLWVEENKVICLYGGEDIQWIRNFTRKAREVAEALHVPLEMLYVGKRNPKEKVRSCNETIAKERLSNVFAAGDDYYDYVWFFWVRLWGLWNSRKKIGMTVENDVIMREILDVLAYDSSNHQGWAAFSWSNRDITKGNAEKLMPVLDNYAQWAYKVDRPDRFVRALDEELQGFHPEHHCTRLILPASDGQISERVVCSECGKTMDKFVLYSCCTD

**17817036_peptide|Vvinifera|GSVIVG01000577001|GSVIVT01000577001**

MATTFIPDKVQRVRQRSGDRMFASDDNGIMKQIHAIHVPDGRDFDVKPLLRIVENILLRTTSSTTLTPALPGIPLGSNQAQLDALEDKTLQDGSSNMIDLLAHTINKISCEISCKCTSGGDAHATAVAVFNILSSYSWDAKVVLALAAFATTYGEFWLVAHLYPTNPLAKSVAILKQLPDILEHTDALKPKFEALSSLIKVMVDVAKCIVQFKELPPQYITPDTPAMVTAIAHIPTAVYWTIRSIVACASQIASLIGMSHEYIASTMDAWELSGLAHKVSNMYGHLQSQLYLCQQHINDKKHIEAYMMLVRLFETPHIDNMKIIRVLIYAKDDQPPLFDGLSKRKVSLDILRRKNVLLFISELEVPHEELFILDQMYQESRQDPTRPESQYEVVWMPMVDRSTPWTEEKNRQFETLKSMMPWYSVDHPSSIDLAVIKYIKEMWGFNKKPLLVVLDPQGRVVNNNAIHMMWIWGSLAFPFTSLREEGLWKGETWRMELLADTIDPIIHNWISEGSYICLFGGEDMEWIRKFCILAKAIARAAGIRLEMLYVGKSNPREKIQKINAIISTDNLSHTLPDLHLVWFFWVRLESMWYSKMQHGKTVESDPIMQEIVSMLSFDGSDQGWVVFSKGSGEMTKAKGENIVRCLSDYDVWKNNVSSKGFLGALNDYLREIHTPHHCNRLILPGTTGSIPERVVCAECGRPMEKFVMYRCCTD

**17817037_peptide|Vvinifera|GSVIVG01000578001|GSVIVT01000578001**

MANTTISAQMQQSVSNRLMSASLIDNPIMNQIHSIHASDGCSRHIDMKPLLRFTENILQNALPTTIDTPATPQEAQTQLNELLEDYDGHYDFLKLYLAQIIKRTSCKISCTCGQSAYAATLEVFKTLSSFTWETKVVLALAAFSVTYGKFWLVAQPSSTNLLAKSIAILEQLPDMLADKEPWKPKFEAPSNLIKTILKVTKCVVEFWELLSEYMTDGRGMPTAAAHIPTAVYWTIHGMVVCTKRTMCLTDMGQEDIDQTMEDWYLSSLDHKLSQIHDYLKEQLAVCRQHIRERREIEAYKMIEHLLKTPQIDNMKILGALICAKAEQLPLFDGLNKKRARLDVLWKKNVLLFISELEVPYQELSILEKMYLESRQDPTKEESQYEVVWLPVVDGSTPRNEEKDRHFETQKALMTWYAVFHPSLLETAAIKYIKEVWGFNKRPMLVALDPMGRVVNPNAIHMIYIWGPTVAFPFSKSREEGLWKEVTWGIELLAAAIHPMIVDWISEGKYICLYGGDDIEWIQRFTDVAKAVESAADIKLEMLYVGKSNLREKVRKNNDSIAQENLSHVLPDLSSVWFFWARLESMWHSKVQHGGENAERDPIMQEIVSMLSFDGGDHGWAVFGRGTPDRVACAECGRPMEKFIMYRCCTD

**17820370_peptide|Vvinifera|GSVIVG01007594001|GSVIVT01007594001**

MAATAQPNRVQQQQQVVKGRDHPKFLRMSDDTTMMKQIQATHTPDGREVEVKPIVQVIEDILNHATPAIDGTLYGNPPHLEALEDRSSQDGLHGILEELAYTIQKLSCELSCKCSGGGDAHATTMAVFNMLSHYSWDAKVVLSLAAFAANYGEFWLVIQLYATNPLAKSVALLKQLPDIIEHGNSLKSRFDAVTKLIKVMLDVTKSIIEFKELPSLYISPDMPPMSSTMAHIPTAAYWTIRGIVACASQIISLIGTSNEYTSWTTESWELSTLAHKVSSIHEHLIQQLIICHQHIEEKKQFESYNNLVRIFEMPHLDNQKVLKTLIYAKEDIQPLLQGNTKARVNIEILRRKTVLLLISDLDLLHEEIVILHKFYREQIKSDVEYEVVWLSVVDRSKPLTEENQNKFHELQKEMPWYTLLHPSLLEPAVVRYIKEVWHFTKKAILVVLDLQGKVVCRNALHMMWIWGNTAYPFTNSKEESLWKEETWRLKLLVDDIDATIFAWVNQGKYICMYGGVNSDWILNFNTAAREVAKAAGIQLEMVYVGKSNAKEQVRKTITFIDSRSLGYCWTDPTNIWFFWTRMESMLYSKTQHGKTIENDSIFAGVLTMLSFDGSDQGWSVICHGPTEMAKAKGDMILKCFTEYSDWSDNVQQNGFMPALNEHLQKLHTPEHCNRLILPGINGDIPEKVVCAECGRPMEKYFMYRCCTD

**17838296_peptide|Vvinifera|GSVIVG01032353001|GSVIVT01032353001**

MSDDNMMVKQIHATHAPDGREFDVKPLFQLVEDILNRATPGVDPLISAAQTRIETSDDRTNQASFFALLEALSFTIDRISCEIAYKSLGGGDAHATTLSIFNLLTSYSWEAKLVLTLSAFAVNYGEFWLLAQISSSNQLAKSMAILKQVPTILEHSGQLKPRFDALNNLIRAMVAITRCIIEFKELPSMYISQDVPALATAMTHIPTAVYWTIRSVVACATQITTLTSMGHEYWISATNEAWELSTMAHKINSILDLLKKQLTLCYQYIDDKRNAETFQMLLNLFESIHIDNMKILRALISPKDDVQPLLEGSTKRRVNIDVLRRKNVLLLISGLSISHDELSILDQIYNESRDHGTRMESQYEVVWIPVVDRSVVWTDAMQDRFVTLQATMPWYSVYTPTLIDKAVIRFIKEVWHFRNKPILVVLDPQGKVVSPNAIHMMWIWGSTAFPFTSLREEALWREESWKLELLVDGIDPTILNWIKEGKFIYLYGGTDLEWIRKFTTTARAVASAARIPLEMVYVGQSKKREQVRKCTTAITVEKLSYCWQDLTMVWFFWTRLESMMFSKIQLGSTVDVDPMLREIKKLISYDKEGGWALHSDSRPCCRFEFPSEVGRIPENIKCPECLRIMEKYITFGCCHDENAISFLFSYLCSNSYKHKLLISY

**17838297_peptide|Vvinifera|GSVIVG01032357001|GSVIVT01032357001**

MSDDNMMVKQIHATHAPDGREFDVKPLFQLVEDILNRATPGVDPLISAAQTRIETSDDRTNQASFIALLEALSFTIDRISCEIAYKSLGGGDAHAMTLSIFNLLTSYSWEAKLVLTLSAFAVNYGEFWLLAQISSSNQLAKSMAILKQVPTILEHSGQLKPRFDALNNLIRAMVAITRCIIEFKELPSMYISQDVPALATAMKHIPTAVYWTIRSVVACATQITTFTSMGHEYWISATNEAWELSTMAHKINSILDLLKKQLTLCYQYIDDKRNAETFQMLLNLFESIHIDNMKILRALISPKDDVQPLLEGSTKRRVNIDVLRRKNVLLLISGLSISHDELSILDQIYNESRVHGTRMESQYEVVWIPVVDRSVVWTDAMQDRFVTLQATMPWYSVYTPTLIDKAVIRFIKEVWHFRNKPILVVLDPQGRVVSPNAIHMMWIWGSTAFPFTSLREEALWKEETWRLELLVDGIDPTVLNWVKEGKFIYLYGGTDMEWIRKFTTTARAVASAARIPLEMVYVGKSNKREQVRKCITSITTDNLSYCWQDLTMVWFFWTRLESMLFSKIQLGRGDDDDSMLREIKKLLSYDKEGGWAVLSKGSFVFVNGHSSTVLPTFTEYNLWKDDVPPKGFDIACMDFHSKLHSDSQPCCRFEFPSEVGRIPEKIRCPECLRIMEKYITFGCCHDENAISALY

**17838298_peptide|Vvinifera|GSVIVG01032359001|GSVIVT01032359001**

MSDDNMMVKQIHATHAPDGREFDVKPLFQLVEDILNRATPGVDPLISAAQTRIETSDDRTNQASFIALLEALSFTIDRISCEIAYKSLGGGDAHATTLSIFDLLTSYSWEAKLVLTLSAFAVNYGEFWLLAQIYSSNQLAKSMAILKQVPIILEHSGQLKPRFDALNNLIRAMVAITRCIIEFKELPSMYISQDVPALATAMTHIPTAVYWTIRSVVACATQITTLTSMGHEYWTSATNEAWELSTMAHKINSILDLLKKQLTLCYQYIDDKWNAETFQMLLNLFESIHIDNMKILRALISPKDDVQPLLEGSTKRRVNIDVLRRKNVLLLISGLSISHDELSILEQIYNESRVHGTRMESQYEVVWIPVVDRSVMWTDAMQDRFETLQATMPWYSVYTPTLIDKAVIRFIKEVWHFRNKPILVVLDPQGRVVSPNAIHMMWIWGSTAFPFTSLREEALWKEETWRLELLVDGIDPTVLNWVKEGKFIYLYGGTDMEWIRKFTTTAKAVASAARIPLEMVYVGKSNKREQVRKCITSITTENLSYCWQDLTMVWFFWTRLESMLFSKIQLGRGDDDDSMLREIKKLLSYDKEGGWAVLSKGSFVFVNGHSSTVLPTFTEYNLWKDDILLLRNPYEWKAIMLYAPNFFPVISIVLFDNID

**17838299_peptide|Vvinifera|GSVIVG01032360001|GSVIVT01032360001**

MLKQIEATHEPDGREFDVKPLLHLVEQIFTCATPKSDVTFDSLDLKTNDVEALEDKTHQAGFISTLEALAYTIDRISCEIRCKCSGGEEAHQRAISVLNMVSSHPWDAKLVLTLSAFAVNYGEFWLVFQSYNSNDLAKSMAILKQVPEILGRSSMLKPQFNSIKDLIKAMLDVANCIVKFRELPSQYITTDESAFSTALANIPIAVYWTIRCVVACASQIARLKGLQGDEHPLSTSEAWEISALVHKVRNIHNHLRDKLDACHKHIDDKRRMEAYQMLLELFKTSHSDNMKVLKALIYARDNQNPLFHGATHRRVDIDVFKDTHVLLLISNLDISHDELEVLEDIYRESLKKRPGIQYEIVWLPIIDQSDPWMESSQKLFENHRARMPWYARHDPLRSPSPEDGAVITFIKKEWHYGRKPILVVLGPQGQVVCQNALHMMWIWKDEAFPFTASREEDLWKEATWKLDFLVDGIDPRISEWIAAGKIICLYGGDDIEWIQRFTTIAKKVAESAGISLEMVYVGKSNPKELVYTNIKTIIEDKLSHHLKGLTSIWYFWVRIESMLYSKMRLGQTVEKDPTMQEILKMLSFDNSHEGWALLSKGSEEITKAKGDSFLTCLRQYNQWEVHVQKKGFLQALKDHLLQIHPPHHCNQFELLVAAGMIPETLVCSECGRKMEKFFVYRCCDV

**17963114_peptide|Mesculenta|cassava4.1_002627m.g|cassava4.1_002627m**

MAMIKQTPTSLQKLIKGDRLMFSSSDDTAMTNQIQATHSPDGREFDVKPLLQLVEDIFTRAAPTIDALAVPATHQARTADALDEKNYHGNFTVIIESLAPVIDRVASEIAYKCAGVVDAHATTMSILNMLSSYSWEAKLVITLAAFATNYGEFWLVAQNYTSNQLAKSVAILKQLPDILEHSSTLKPRFDAVKNLIKVMLDIARCIVEFKELPSQYISMDVTALSTAIAHIPITVYWTIRSVVACATQIIGLIGQGQEYIASTTEAWELSSLAHKLSNMHTHLATQLGICYKHIDEKKQLETYQNLLHLFEMTHIDNMRILKALIYAKDDLLPLVEGTTKRRVNIEVLRRKYVLLLISDLDIPQQEISILEQIYNESRLHPTKQESQYEIVWLPIVDPTSLRNDENMQRKFESLQAGMTWYSVHHPSLIDRAVAKFVKQEWHFGKKPILVVLDPQGRVACPNALHMMWIWGSLAFPFTTMREETLWKEESWRLELLVDGIDPIIHDWMTEGRYICLYGGEDMDWIRKFTSTARAVAQATGIPLGMVYVGNSNPKDRVRKNIATIIVEKLSHYWQDPTSIWYFWVRIESMWRSKNQLGKTPENDPIMKEIMTMLSFDSSHSGWALFTRGSDEMVKAKGAPFLTCLSNFSSWKDEIEKKGFMPTLRDQLKDLHTEHHCNRLVLPGAAGMIPERIICSDCGRTMERFIMYQCCDE

**17963117_peptide|Mesculenta|cassava4.1_002917m.g|cassava4.1_002917m**

MLALSDETSVMKQIQATHCPDGREFDVRPLLDIVEDILKRATLQVETTLTPTASHAESDDKNRCNFISKLETLSFVIDRICCEICYNSLGGSDAHAITLSIFSMLSNYSWDAKLVLTMAAFASNYGEFWLLAQIYSSNQLAKSVAILKQLPIILEHSGLLRPRFEALNHLIRIMLEVTGCIVEFRELPSTYISPDMPALSTATSLTPTAVYWTIRSIVACATQISSLTTMGHEFATTEAWELSTLAHKLRNIDEHLKKQLSICYQHIEEKRTVEAYQVLVTLFETIHIDNIKILKTLIYSKDDVPPLVEGSTKKRVKVEVLMKKNVLLLVSGLDISQDELSILEQIYKESRLHPTRAEVQYEVVWVPIVDHTVPWTECMQKQFESLQDTMPWYVVHHPSMIDKVVIKFVKEVWHYRSRPILVVLDSQGRVVCPNALHMMWIWGSHAFPFTSLREESLWKEETWKLELLVDGIDKNIMNWINEERYILLYGGDDIEWVRKFTSTARSVALEASIPLELVYVGKSSKREKVQRVIATIIEEKLSYVWEDLTMIWFFWTRLESMLYSKLQLGKIDEEDSTMLEIKKLLSYDKEGGWAVFAKGSDIVVNGHSSAVLPTLTEYEVWKEQIEIKGFELSFKEHHEKIHCLSHTCCRFEFSKSASRIPEQLRCPECHRVMEKYISFLCCHED

**17963355_peptide|Mesculenta|cassava4.1_002657m.g|cassava4.1_002657m**

MAVVPHRLSNPRSERHMFSSSDDNAMMKQIQATHAPDGRDFSVRPLLQLVEDIFQRAAPTGLATLVHHQQAQLDALDDKALQNGVDEILDVLSYTINRIACEMSCKCSGGGDAHATTLAIFNLLSSYSWDAKVVLALSAFAVNYGEFWLVAQLYLTNPLAKGVALLKQLPDILERADALNPKFVALNNLIMATLDVAKCIVEFKELPSQYITPDTPEMLTATAHIPTAVYWTIRSIVACASQITGLIGTGHEYIASTTEAWELSSLAHKVKSIHEHLMKQLTLCYHHIDEKRHNEAYQTLIRLFETSHIDNMKILRALIYAKDDQPPLHHGATQKRVTLEVLRRKNVLLYISDLELSLEELSMLNQMYTEARQQPTRTESQYEVVWLPVVDRSTPWNEVKQKQFESLQSMMPWYTVYHPSLLDPAVIRYIKEVWRFNKKPLLVVLDPQGRVVNPNAIHMMWIWGSIAFPFTSHREEALWKEETWRIELLADAIDANIPDWIHAGKYICLYGGEDIDWIRKFTVTADAVARAANIQLEMLYVGKSNPREKVRKNNDIIKAENLSHTLQDLTMIWFFWVRLESMWHSKVQYNRTVENDSIMQEIVTMLSFDGSDQGWAVISRGSGGGDEIAKAKGGDILKSFNEFQSWKHIIDEKGFVPALNDYLHGHHDPHHCNRLILPGTTGSIPERVVCAECSRPMEKFIMYRCCTD

**17982535_peptide|Mesculenta|cassava4.1_002528m.g|cassava4.1_002528m**

MEMAKQLPASMQMEMAKQLPASMQKLIKGDKLIFSSADDNAMTKQIQGTHSPDGREFDVKPLLHLVEDIFNRAAPTIDAIALPATQQARTADSLDEKTYQGSFIAILESLAFVIDRVATEIAYKCTGGGDAHATTMSILNMLSNYSWDAKLVIALAAFSMNYGEFWLLAQSYTSNQLAKSVAILKQLPDILEHSSMLKPRFDAVKNLIKAMIDIAKCIVELKELPPQYISLDITAMSTAMAHIPITVYWTIRSIVACASQITGLIGLGHEYISTTEAWELSSLAHKLSNMHSHLATQLGICYKHIDEKKHLESYQNLLHLFEMAHIDNMRVLKALISAKDDQLPLIEGTTKRRVNIDVLRRKNVLLLISDLDILQEEIAILEQIYNESRLHPTKHESQYEIVWLPILEPTVWNENMQKKFESLQTGMTWYSVYHPSLIDRAVIKFVKQEWHFGKKPILVVLDPQGRVACPNALHMMWIWGSLAFPFTTTREEALWREESWRLELLVDGIDPIVMNWMSEGRYICLYGGEDMDWIRKFTNTARAVAQASGIPLGMVYVGKSNPKERIRKNIATIIVEKLSHYWQDLTSIWYFWVRIESMWRSKNQLGKTAENDSIMKEIMQMLSFDSSQGGWAIFTRGSDEMVKAKGVPFLTCLSNYSNWKDQIQVKGFMPTLSDELKNLHTEHHCNRLVLPGAAGLIPERIICSECGRTMERFIMYQCCDE

**17982547_peptide|Mesculenta|cassava4.1_002579m.g|cassava4.1_002579m**

MAFRSPLAAAPGTGATQKLIKSDRSMLALSDENSMMKQILTTHAPDGREFDVRPLLHVVEDILSRATLQVESTLTATQAQADIEDKNRQGNFVAMLESLSFVIDRIACEISYKALGGSDAHATTMSLFNMLSSYSWDAKLVLTMAAFALNYGEFWLLAQIYSSNPLAKSMAILRQLPVILEHTGPLKPRFDALNKLIRVMTDVTRCIVEFKELPSIYISSEVPALATAIAHIPTAVYWTMRSVVACATQITSLTTLGHEFATTEAWELSTLAHKLQNIHEHLKKQLFFCHQHIEEKRNVEAYQMLLNLFDAVHIDNMKILKALIYAKDDIPPLLDGSTKRRVNIDVLRRKNVLLLVSGLDISHDELSILEQIYNESRLHATRLDSQYEVVWIPIVDPSVTWNDSKQKQFEALQATMPWYSVHHPSIIETVVIKFMKEVWHFRNKPILVVLDPQGKVVCPNALHMMWIWGSNAFPFTSLREESLWKEETWRLELLVDGIDPTILNWIKEGKYIFLYGGDDVEWVRKFTNQARAVAQAARIPLEMVYVGKSSKRDKLQRVIATITVEKLSYVWQDLTMIWFFWTRLESMLFSKIQLGKIDEYDPMMQEIKKLLSYDKEGGWAVLSKGSNIVVNGHSTTVLPTLTEFDIWKENVPVKGFDLSFKEHHDKLHGVTHPCCRFEFLSTGRIPEGMKCPECLRNMEKYITFLCCHEETIEEML

**17986491_peptide|Mesculenta|cassava4.1_002622m.g|cassava4.1_002622m**

MAIVPHKLSNPRSERHMFSSSDDNAMMKQIQATHAPDGREFSVRPLLNLVEDIFQQAAPTGLATIVHQQVGAQHAQLEAMDDKALQNGSHEMLEVLSYTINKISCEISCKCSGGGDAHATTLAIFHLLSSYSWDAKVVLALSAFAVNYGEFWLVAQLYLTNPLAKGIALLKQLPDIIERSAVLKPKFEALNNLIRATLDVAKCIVEFKELPSQYISPDAPEMLSATAHIPTAVYWTVRSIVACATQIIGLTGTGHEHIASTTEAWELSSLAHKVRSIHEHLMAQLTRCYHHIDEKRHLEAYQTLVRLFETIHIDNIKILRALIYAKDDQLPLFVGAKKERASLDVLRRKNVLLYISELELQYEELEMLRQIYEETRQHPTRAESQFEVVWLPVVNRSTPWNDEKQKQFESLRSMMPWYSVFHPSLLDPAVVRYIKEVWHFNKKPLLVVLDPQGKVVNPNAIHMIWIWGSTAFPFTSNREESLWKEETWRIELLADTVDPNIPAWIEGQKHICLYGGEDMDWIRKFTKTADAVARAANIQLEMLYVGKSNPRDKVRKNNSTIQAEKLSHILPDLTMIWFFWVRLESMWHSKVQHNRTVENDLIMQEIVTMLSFDGSEHGWAVISRGSGLQGHMAKAKGTDILECFEDFQSWNHIATEKGFVPALNDYLHGHHNPLHCNRLILPGTTGTIPERVVCAECNRPMEKFIMYRCCTD

**18097818_peptide|Csinensis|orange1.1g008311m.g|orange1.1g008311m**

MAQWPGHDDSQQLIRRDRSMVSISDDNMMMRQIQATHAPDGREVDVKPLFNLVEDILNRATFQTDIDETDAQAHLEIEDNAQQAGFLAMIDAISFTIDRISCEIACKALGGSDAHATTLSLFSMLSNYSWDAKLVLALAAFALYYGEFWLLAQIYSSNQLAKSMAILKQLPSIMEHTSGPLKSRFDTLNNLIKAMMGVTRCIVEFKDMPSNYITQDAPEFSSAMAHIPIAVYWTIRSVVACATQITTLTGMGHEFVISTSEAWELSTLAHKLKNIHESLKKLMATCYRHIEEKKSHEAYQMLVKLFDSIHIDNMKVLKALIYAKDDLQPLVDGSTKRRVNIEVLRRKNVLLLISDLDISQEELSILEQIYNESRLHLTRQESHYEVVWIPIVDHFIHWDDPTQKKFETLQSSMPWYTVYHPTLIDRAVIRYVKDVWHFKNKPILVVLDPQGRVVSPNALHMMWIWGSNAFPFTSLREEALWKEETWRLELLVDGIDPLVLDWIKEGKYIFLYGGDDVEWVRKFTTAARSVANTARIPLEMVYVGKSTKRDKCGESWQPLQLKSLVTSGKT

**18102906_peptide|Csinensis|orange1.1g005245m.g|orange1.1g005245m**

MATSIVPYRMQPVGRERHMFATSDDNAMLRQVQATHAPDGREFNVKPLLYIIEDIFQRAAPSFPGFIQETQAQLDVLDDKAFQSGFFDMLDLLSSTINRISCEISCKCSGGGDAHATTLGIFNIVTSYSWDAKVVLALAAFALNYGEFWVVAQLFPVNPLAKSVALLKQLPEILERADTMKPRFETLSNLITAMLDLTKCIVEVKELPSDYITPDTPEMAAVTAHIPTAVYWIIRSIVACAGQILGLIGMGHEYIISTTETWELSSLAHKINSIYNHLLQQLKLCHQLIEEKRQIESYQALVRLMETIHIDNMKVLNRLLIHTKDDQLPLVECPTKRKVSIDVLRRKSVLLLVSDLDVSNEELFLLEQMYRESRQLSSRTESQYEVVWLPIVDRSTPWTEAKEHKFEALQYMMPWFSVHHPSAIDPAVIRYAKEKWDFRKKPILVVLDPQGRVVNQNALHMMWIWGSVAFPFSVAREEALWKEETWRIDLLADSVDPVIPTWIMEQKHICLYGGEDLEWVRKFTALMGAVARAAGIALEMLYVGKSNPKEKARRIISTISVEKLSHTLPDPTLIWFFWVRLESMWHSKMKFGTKVQQDPIMQEIVTMLSFDGSDQGWAVISRGPHMAKAKDETILKCLTEYTTWEPNVPEKSFVVAMNDYLNENRTPYHCNRLILPGEAGRIPEKVVCAECGRRMEEFIMYRCCTD

**18132214_peptide|Csinensis|orange1.1g044391m.g|orange1.1g044391m**

MQEVLARSKGRMLSATDDSAMMKQVQASHAPDGREVDVRPILSIIEDIFRRATPSTIDGVGTREHVDALDNNASPAALSGMLDALSCDIRKISCEISCKCSGGEEAHATTMDLFNILSVYSWDAKMVLSLAAFALNYGQFWLSAQLCNKNSLAKSMAVLKQLPNVLEHYNALKPQLDALIKVMLDLTKCIVEFKQLPSQYISTDAQAMSTAMADTPAAAYWTFRSIVACHSQILSLAGLRDEYTASNTDAWELASLAHRVSRILEHFKKLIAICYQQIDENRQIEAYHNLVRLLETIHMDNMKVLRALIYAKDDIQPVVDGSSRTRVNIEVLRRKHVLLLISSLDLSDEEILVLHLYREHKAREEFDYAIVWLPIVDRSIAWDEGYRQKFEQLQAMMPWYTVQHPTIIEPAVVKYAKEVWKFSKKAILVPVDPQGRILNQNAFHMLWIWGNLAFPFSAEKEAALWKAESWRLELLIDDIDATILEWMKEERYICLYGGGDIEWIRRFTTSAKAVARAAQINLGMAYVGKNNAKERFRKISRIVIQENLSHTLTDPTEVWFFWARLESMLYSKLQHGATVEDDHIMQEVMTILSFDGSEQGWAIFWRGTHEMARAKGEMAVDCMMEFEKWKDDADQMGFVAGLNNYLQRVHTPRHCNRLILPDIHGPIPERLACAECGRTMEMFFMYRCCPE

**18132848_peptide|Csinensis|orange1.1g046837m.g|orange1.1g046837m**

MALRNDLRMISASDDSAKMMKQVLANHAPAGHEVDVRPILSIIEDIFRRATATPSNIDGVPNGKREHMDALHDNKSLAAFSTPEPLSDIIHKICCEISCKGGGDALATTMALFKTLSSYSWDAKMVLSLAAFAVNYGEFWLVAQLCTQNSLANSVAVLKQLPEIPTGYDALKPQFDELNNLIGAMLDLTNCIVEFKQLPSRYISNDGKAMSMDQDHYHAAYWTFRSIVACNSRILSFRGLYTPSTTHELELSTLTYKVSNIHVSENLTKQIEFWRQQIDKIRQIKEYNNLVRILGKHNRDNIKVLRALIYAEDLVDGETRTTVQIHVLKKKHVLLLISRPDDISQEEILFLSNMYKDLKESKECRIVWLPIVDGSIDRQQALDKFKNLQKRMPWYSIQDPAMIQPAVIKYVKEEWKYSKKAIIVSVDPQGRILNQNAFHTLWIWGISAFPFTAETEEALWKEKPWTLELLVGDIDATILEWMKEERFICLYGGNDEAWIRKFRNSAKDVASKAQINWGMAYVGKKNAKKRLEEISSSITKTESSHIVIDATKMWFFWARLERMLYWKLQHGMGKTQETARAKGTAAIDCMEKFHEWQDDALRNGFIQALNNYLQKIHPGKHCNRLILAGVNGAIPGTVQCADCGRDMEMFFMYHCCPE

**19658887_peptide|Athaliana|AT3G01680|AT3G01680.1**

MESLIKSQHAQQLAGHKNTTGKTPSMEMIPATGLAMSSDESMMLKLIQQTHSPDAREVQVRGLLSLVEDILDRATLDSEDTNASMLPLPTEDKLMQSSMMSVLDSVSYAIDRVACEIAYKSLTGSDSHEITMSVFEHLSSFQWDGKLVLTLAAFALNYGEFWLLVQFYSKNQLAKSLAMLKLVPVQNRVTLESVSQGLNDLIREMKSVTACVVELSELPDRYITPDVPQLSRILSTIPIAVYWTIRSVIACISQINMITAMGHEMMNTQMDLWETSMLANKLKNIHDHLAETLRLCYRHIEKQRSSESLKVLHSLFDTTHIDNMKILTALVHPKPHITPLQDGLTKRKVHLDVLRRKTVLLLISDLNILQDELSIFEQIYTESRRNLVGVDGKSHMPYEVVWVPVVDPIEDFERSPILQKKFEDLRDPMPWYSVDSPKLIERHVVEFMRGRWHFMNKPILVVIDPQGNEASLNALHMIWIWGTEAFPFTRSREEELWRRETFSLNLIVDGIDSVIFNWIKPDNYIFLYGGDDLDWIRRFTMAAKATAKDSNVNLEMAYVGKRNHSHREQIRRISEVIRSENLSHSWAEPALMWFFWTRLESMLYSKIQLGKADDHDDVMQGIKKILSYDKLGGWALLSKGPEIVMIAHGAIERTMSVYDRTWKTHVPTKGYTKAMSDHHHDEVLRETGKPCGHFDFHITARSGRIPEKMNCFECQRPMEKYMSFSCCHDEKLHEDENYNF

**19663248_peptide|Athaliana|AT3G01670|AT3G01670.1**

MAQRFQLNSQTLPTADPLKRVSLIPRSAEQRLADNAGERRPLAPRSHEDNPFGGHTDDHHVAAPADHNKVMDHNSENLGSIVPKTAHYPHPSEEILDANIRHSMVPKSLGPNSLGGRFGPGKKQAFHRNGRPMFSLSDDRVMADRVLKTHSPDMIFFDVTSLLSVVNDIFKSHVPSIDSSAPKPSLVFKDYADHTSFETFADLIDQISCEIDCKCLHGGESHGMMTSGLHLDSRNTTTFSVLSLVSKYRWDAKLVLVLSALAVKYGVFLLLAETHATNQLTKSLALIKQLPSIFSRQNALHQRLDKTRILMQDMVDLTTTIIDIYQLPPNHITAAFTDHIPTAVYWIVRCVLICVSHISGASGFKQDQIMSFMEVSEIHENSERLRKINAYLLEQFKKSKMTIEEGIIEEEYQELIQTFTTIIHVDVVPPLLRLLRPIDFLYHGAGVSKRRVGINVLTQKHVLLLISDLENIEKELYILESLYTEAWQQSFEILWVPVQDFWTEADDAKFEALHMNMRWYVLGEPRKLRRAAIRFVREWWGFKNRPILVALDPKGQVMSTNAFPMVWIWQPFAHPFTTARERDLWSEQEWNLEFLIDGTDPHSLNQLVDGKYICLYGGEDMQWIKNFTSLWRNVAKAANIQLEMVYVGKRNPKNGIQPIINTIREENLSHTLPDLFQIWFFWTRVESMWESKQRMLKAHGIKGREGFKEEEKDLVLQEVVAMLGYGGEGDGWGLVSKASDMMVRAKGNLFSRGLAEFNEWEVNIPTKGFLTALNDHLLMRLPPHHCTRFMLPETAGIIPNEVECTECRRTMEKYYLYQCCLE

**20181725_peptide|Thalophila|Thhalv10020146m.g|Thhalv10020146m**

MESLIKSQHAQKLAGHSGKTPATEMIPATTGLTMSSDESMMLKLIQHTHSPDAREVQVRGLLSLVEDILDRATLNSDDSNSSMLPLPTEDKLMQSSVMSVLDNVSYAIDRVGCEIAYKSLTGSDAHEITMSVFEHLSSFHWDGKLVLTLAAFALNYGEFWLLVQFYSKNQLAKSLAMLKLVPVQNRVTLESVSQGLNDLIQEMKSVTACVVELSELPDRYISLEDPQLSRIVSTIPIAVYWTIRSIVACISQINMITAMGHEMMNTQMDSWETSMLANKLKNIHDHLSETLRLCYRHIEKQRSSESLKMLHSLFDTTHIDNMKILTALIHPKNHTTPLQDGLTKRKVHLDVLRRKTVLLLISDLNILQDELSIFEQIYTESRRNLLGMDGKSHMPYEVVWVPIVDPIEDFERSPILQKRFEALRAPMPWYTVDSPKLIERHVVEFMRERWHFMNKPILVVIDPQGNEASLNALHMIWIWGTEAFPFTRSREEELWRRETFSLNLIVDGIDSVIFNWINPDNYIFLYGGDDLDWIRRFTMAAKATAKDSNVKLEMAYVGKRNHSHREQIRRISEAVSAENLSHSWTEPALMWFFWARLESMLYSKIQLGKADDHDEVMQGIKKILSYDKLGGWALLSKGPEIVMIVHGAIERTVSVYDRTWKTHVPTKGYTKAMYDHHHDEVLRETGKPCGHFDFHITARSGRIPDKMNCFECHRPMEKYMTFSCCHDDKLLDQDENYNF

**20182542_peptide|Thalophila|Thhalv10020071m.g|Thhalv10020071m**

MAQRFQLNPKPLTDPPADPLKRVSLIPRPSAEQKLTENLGERRPLAPRTHEDKPFGEQSDDHHVAAPPKTMDHKKATDHETEQIGSIVPKTTAHHPHSSEELLDANSRHSLAPRSLGHNSLGGRFGPGKKQAFHRNGRPMFSLSDDRVMADRVLKTHSPDMVFFDVKSLLSVVDDIFKSHVPSIDSPAPKPSLVFKDYADHTSFETFADLIDQISCEIDCKCLHGGESHGMKTSGLHLDSRNTTTFSVLSLVSKYRWDAKLVLVLAALAVKYGVFLLLAETYATNQLTKSLALIKQLPSIFSKQNALHQRLDKTRSLMQDMVDLTTTIIQIYQLPPNHITAAFTDHVPTAVYWIVRSVLICAAHISGVSGFKQDQIMSFMEVSEIHENSERLRKINAYLIEQLKKSRMTIEDGIIDEEYQELIQTFTTIIHVDVVPPLIRFLRPIDFLYHGAGDSKRRVGINVLTQKHVLLLVSDLENIEKELYILESLYTEAWQQSFEILWVPVQDYWTEADDAKFESLHSNMRWYVLGEPRRLRRSAVRFVREWWGFKNRPILVALDPKGQVMSTNAFPMVWIWQSFAYPFTTARERDLWGEQEWNLEFLIDGTDPHSLNQLVDGKYICLYGGEDMQWIKNFTSLWRNVAKAANIQLEMVYVGKRNPKNGIQPIINTIKEDNLSHTLPDLFQIWFFWTRIESMWESKQRMLKAHGTKGRERSKEGFKEEEKDLVLQEIVAMLGFGGEGDGWGLVSKASDLMVRAKGNLFSKGLAEFNEWEANIPTHGFLKALNDHLLMHLPPHHCTRFMLPETAGIIPNEVECTECRRTMEKYYLYQCCLE

**20786278_peptide|Cclementina|Ciclev10000431m.g|Ciclev10000431m**

MAQWPGHDDSQQLIRRDRSMVSISDDNMMMRQIQATHAPDGREVDVKPLFNLVEDILNRATFQTDIDETDAQAHLEIEDNAQQAGFLAMIDAISFTIDRISCEIACKALGGSDAHATTLSLFSMLSNYSWDAKLVLALAAFALYYGEFWLLAQIYSSNQLAKSMAILKQLPSIMEHTSGPLKSRFDTLNNLIKAMMGVTRCIVEFKDMPSNYITQDAPEFSSAMAHIPIAVYWTIRSVVACATQITTLTGMGHEFVISTSEAWELSTLAHKLKNIHESLKKLMATCYRHIEEKKSNEAYQMLVKLFDSIHIDNMKVLKALIYAKDDLQPLVDGSTKRRVNIEVLRRKNVLLLISDLDISQEELSILEQIYNESRLHLTRQESHYEVVWIPIVDHFIHWDDPTQKKFETLQSSMPWYTVYHPTLIDRAVIRYVKDVWHFKNKPILVVLDPQGRVVSPNALHMMWIWGSNAFPFTSLREEALWKEETWRLELLVDGIDPLVLDWIKEGKYIFLYGGDDVEWVRKFTTAARSVANTARIPLEMVYVGKSTKREQVRRIMATLAVEKLGHFWQDLTMVWFFWTRLESMLLSKIQLGRADDHHDPMVPQIKKLLSYDRDGGWALLSKGSQVLVNGHGTTVLPTLLEYDIWKEQVPIKGFDDSFIDHHQKIHGVAHPCCRFEFSAHSGRIPASMTCPECHRYMEKFTTFCCCHDDHVPGSLF

**20803198_peptide|Cclementina|Ciclev10030843m.g|Ciclev10030843m**

MATSIVPYRMQPAGRERHMFATSDDNAMLRQVQATHAPDGREFNVKPLLYIIEDIFQRAAPSFPGFIQETQAQLDVLDDKAFQSGFFDMLDLLSSTINRISCEISCKCSGGGDAHATTLGIFNIVTSYSWDSKVVLALAAFALNYGEFWVVAQLFPVNPLAKSVALLKQLPEILERADTMKPRFETLSNLITAMLDLTKCIVEVKELPSDYITPDTPEMAAVTAHIPTAVYWIIRSIVACAGQILGLIGMGHEYIISTTETWELSSLAHKINSIYNHLLQQLKLCHQLIEEKRQIESYQALVRLMETIHIDNMKVLNRLLIHTKDDQLPLVECPTKRKVSIDVLRRKSVLLLVSDLDVSNEELFLLEQMYRESRQLSSRTESQYEVVWLPIVDRSTPWTEAKEHKFEALQYMMPWFSVHHPSAIDPAVIRYAKEKWDFRKKPILVVLDPQGRVVNQNALHMMWIWGSVAFPFSVAREEALWKEETWRIDLLADSVDPVIPTWIMEQKHICLYGGEDLEWIRKFTALMGAVARAAGIALEMLYVGKSNPKEKVRRSISTITVEKLSHTLPDPTLIWFFWVRLESMWHSKMKFGRKVQQDPIMQEIVTMLSFDGSDQGWAVISRGPHMAKAKDETILKCLTEYNKWEPNVPEKSFVVAMNDYLNENRPPHHCNRLILPGEAGRIPEKVVCAECGRRMEEFIMYRCCTD

**20807184_peptide|Cclementina|Ciclev10023346m.g|Ciclev10023346m**

MALRNDLRMISASDDSAKMMKQVLANHAPAGHEVDVRPILSIIEDIFRRATATPSNIDGVPNGKREHMDALHDNKSLAAFSTPEPLSDIIHKICCEISCKGGGDALATTMALFKTLSSYSWDAKMVLSLAAFAVNYGEFWLVAQLCTQNSLANSVAVLKQLPEIPTGYDALKPQFDELNNLIGAMLDLTNCIVEFKQLPSRYISNDGKAMSMDQDHYHAAYWTFRSIVACNSRILSFRGLYTPSTTHELELSTLTYKVSNIHVSENLTKQIEFWRQQIDKIRQIKEYNNLVRILGKHNRDNIKVLRALIYAEDLVDDFYLCKQVQIHVLKKKHVLLLISRPDDISQEEILFLSNMYKDLKESKECRIVWLPIVDGSIDRQQALDKFKNLQKRMPWYSIQDPAMIQPAVIKYVKEEWKYSKKAIIVSVDPQGRILNQNAFHTLWIWGISAFPFTAETEEALWKEKPWTLELLVGDIDATILEWMKEERFICLYGGNDEAWIRKFRNSAKDVASKAQINWGMAYVGKKNAKKRLEEISSSITKTESSHIVIDATKMWFFWARLERMLYWKLQHGMSVEKDNIMREMMTLLSFDGSPWLSCLDSRAGEPVLPAAVGWVSSPLDLEPEWPYASYRKLTSRMTCSRMSRIFLLLFPRAPGFYGTYRSDQGWAIFWRGKTQETARAKGTAAIDCMEKFHEWQDDALRNGFIQALNNYLQKIHPGKHCNRLILAGVNGAIPGTVQCADCGRDMEMFFMYHCCPE

**20809148_peptide|Cclementina|Ciclev10019128m.g|Ciclev10019128m**

MQEVLARSEGRMLSATDDSAMMKQVQASHAPDGREVDVRPILSIIEDIFRRATPSTIDGVGTREHVDALDNNASPAALSGMLDALSCDIRKISCEISCKCSGGEEAHATTMDLFNILSVYSWDAKMVLSLAAFALNYGQFWLSAQLCNKNSLAKSMAVLKQLPNVLEHYNALKPQLDALIKVMLDLTKCIVEFKQLPSQYISTDAQAMSTAMADTPAAAYWTFRSIVACHSQILSLAGLRDEYTASNTDAWELASLAHRVSRILEHFKKLIAICYQQIDENRQIEAYHNLVRLLETIHMDNMKVLRALIYAKDDIQPVVDGSSRTRVNIEVLRRKHVLLLISSLDLSDEEILVLHLYREHKAREEFDYAIVWLPIVDRSIAWDEGYRQKFEQLQAMMPWYTVQHPTIIEPAVVKYAKEVWKFSKKAILVPVDPQGRILNQNAFHMLWIWGNLAFPFSAEKEAALWKAESWRLELLIDDIDATILEWMKEERYICLYGGGDIEWIRRFTTSAKAVARAAQINLGMAYVGKNNAKERFRKISRIVIQENLSHTLTDPTEVWFFWARLESMLYSKLQHGATVEDDHIMQEVMTILSFDGSEQGWAIFWRGTHEMARAKGEMAVDCMMEFEKWKDDADQMGFVAGLNNYLQRVHTPRHCNRLILPDIHGPIPERLACAECGRTMEMFFMYRCCPE

**20809861_peptide|Cclementina|Ciclev10019109m.g|Ciclev10019109m**

MQELFTRSQRRMFSASDDSAMMKQVQATHAPDGREVDVTPILSIIEDIFRRATPSDIDGVLNGAREHMDALDDNISLDAFSGMLEALSYTIHKISCEISCKCSEGGDAHATTMALFNTLSGYKWDAKMVLSLAAFAVNYGEFWLGAQLCTNNSLAKSVAVLKQVSNVLEHYNALKPQFDALNNLIKVMLDLTKCIVEFKQLPSQYISTDAQAVSIAMAHIPAAAYWTFRSIVACYSQTLSLTGLRDAYTASTTEAWELSSLAHKVSSILEHFKKLIAICYQQIDENRQIEAYHNLVRLLETIHMDNMKVLRALIYAKDDIQPLVDGFTKNRVNIDVLRKKHVLLLISSLDLSAEEILVLDQMYRDPKASEEYHYAIVWLPIVDRSIAWDDGYQQKFEQLQAMMPWYTVQHPTIIEPAVVKYIKEVWKFSKKAILVPVDPQGRILNQNAFHMLWIWKNLAFPFTAEREAALWKAESWRPELLVDGIDATILEWMKEERFVCLYGGEDIEWIRQFTNSAKAVARAVQINLGMAYVGKNNAKEKLGKISSIVIQENLSHTLADSTAMWFFWARLESMLYSKLQHGATVENDRILKEVTTVLSFDGSEQGWAIFWKGTTHEMARAKGKVATDCMVEFEKWKDDAYQNGFVPGLNNYLERVRTPDHCNRLILPGIHGPIPETVACADCGRVMEMFFLYRCCPE

**20811575_peptide|Cclementina|Ciclev10019121m.g|Ciclev10019121m**

MAPRNDRRMIPASDDSAKMMKQVLANHAPDGHEVDVRPILSIIEDIFRRATATPSKIDGVPNATHEHMDALHDNKSLVAFSGTPEPLSDIIHKICCEISCKCSGGGDALATTMALFNTLSSYSWDTKMVLSLAAFAVNYGEFCLVAQLCTENSLANSVAVLKQLPEIPAGYDGLKPQLDELNNLIGAMLDLTNCIVEFKQLPSQYISNDGQAMSMDQAHYQAAYWTFRSIVACNSRILSLRGLRHEYTASTTDELELSTLAHKVSNIHVYENLKKQIDFWRQQIDKIRQIEEYNNLVRLLQKNNQDNIKVLRALIYAEDLVDGETRTTVQIDVLKKKHVLLLISSLDNLSQEEILVLRKMYKDLKASKECRIVWLPIVDRSIDWQQTLYKFESLQKRMPWYTIQDPATIQPAVIKYIKEEWKYSKKAILVSVDPQGRILNQNAFHTLWIWGISAFPFTAETEEALWKEKSWTLELLVGGIDATILQWMKEERFICLYGGNDAAWIRKFTNSAKAVASKAQINWGMAYVGKKNAKKRLEEISSIITKEDSSHIVIDGTAMWFFWARLERMLYSKLQHGMSVEKDNIMREVMTLLSFDGSDQGWAIFWRGKTQETASAKGTAAIDCMEKFHEWQNDASQTGFIEGLNNYLHKIRTGKHCNRLILAGVNGAIPGRVPCADCGHDMEMFFMYRCCPE

**22037693_peptide|Acoerulea|Aquca_011_00406|Aquca_011_00406.1**

IIPSRCHPSVLFYMAASSSATTTFPLQSEMIIPDTFTSDDYNFNVKPLLEIVENILQHSILNSAQGEYSHGGVKESMADDDTVRFVLYDIQKIGHEVLFNGEDESMLNEIHKILSTYAWGAKPVLALAAFAMTYGNFWLTVQMHSTNPLAYSVAVLKGILNISNLSTTVLKSWFDNLNDAIKAMIEFYDFPSELITSEDSSLHSEAESISHTGVNWIIKAVVACATHFNSLFGRSYQLLINTKSYGDTEEKKCNQLHQLLLQLLGKETTQANNTKVLSILIPLKDDNPPLIHASTNTRVGVEVLRRKTVMLLLSDLDITEEELIILRQIYNDAYLEKSERPYEMVWLPIVDNSTEWTDAKQERFNRLQSLMPWFSVHHPSLIDPNVIEFIKEQWHFDRKPLIVVLNPQGSLICPNAMHMIWIWGIASFPFSRKREEDLWNEEKWTIEFLVDGIDQSILTWLAGGNYICLYGGDDINWIRKFTSTMRDVAHEAQVHLEMVYVGTSKNKEQVKKNMTTISIEKLSHCWEDPVSIWFFWLRLKSMWYSKIRHKQTVEDDAMMRGIVTMLTFDSSNGWAVIGKRTGGMALVPGWKILETLIKFDEWKDNIAGERFVEALIKATGSNKIQEHCLKLVLPKIPGKMHDVSCSECGNPMEKYILYECCDK

**22037792_peptide|Acoerulea|Aquca_011_00408|Aquca_011_00408.1**

MAMQSNMQPHLQPLTQPLTQSQVQSLAQPLTQSHMQPLVQSHLQPLTQSHMQPLQQSLAQSQMQPLPQSLAQSHMQPLTQSLMQPQPHMQPLGVVPQPKGQPVQNFFRGDRHLFSTSDDTVMMKQVQATHAPDGREVDVKPLLNVVEDILNRSTPGIDGIMHATSQAQIDAFEDKTQHIDFVGMLEALAYTIHKISCEISCKCSGGGDAHSTTLAVFNTLSSYSWDAKLVLALAAFGVNYGEFWLVAHLYPTNPLAKSIALLKQLPDLLEHNELLRPRFEALKNLIKEMLDVTKCIVEFKELPPQYINYETPVMAAALTHIPTAVYWVIRSVVACSAQIISLIGLGHEYMTTTTETSWDLSSLGHKVSSIHGHLRKQLALCYQHIDGKRHLEAYQTLVRLFETIHIDNMKILRALIYAQDDILPLYDGSTKKRVNLEVLRRKNVLLLISDLDIPHEELSILDQMYRESRHQPNRPESSYEVVWLPVVDRSTPWTDDKQKQFETLQFMMPWYSVHHPSLLDQAVIKYIKEVWKFAKKPILVVLDPQGRVANQNAVHMMWIWGSLAFPFTSMREDALWKEESWKLELLVDGIDQNILNWIGENKIICLYGGEDMEWIRKFTLAARAVSAAIRTPIEMVYVGKSNPKERVRKNNGMISAEKLSHFWPDLTSIWFFWVRLESMWYSKMQHGKTVENDPIMQEIMTMLSFDGSEQGWAVISKGSAEMTKAKGETILTSFTDIEQWKEHVEPKGFVNALTEYLHQLHTPHHCNRLILPGTTGSIPEMVVCAECGRAMEKFVMYRCCTD

**22060722_peptide|Acoerulea|Aquca_056_00038|Aquca_056_00038.1**

MNNTKPTSRLSMQTLEQPRTMNRLPMQTLEPKTTTGSPMQSFEPKAAQTTLEPKATTELPKQLLEPKTMTGFPMQRVDPMLQLMRSDRHLFSSSDTSMMMRQIQATHVPDGREVDVRPLLHVIEDIVQRSTPNVLGVGHVNATTQSHFDVFEDKTHQDGFMGILEAVAYTIHRISCEVSSKCLDGADAHTTTLSLLNMLSSFSWDAKVVLALAAFAVNYGEFWLLVQLYPTDHLAKSIMLLKQLPDVLEHNESLRSLVKAMLDLTKCIVELSQLPAHYITPDVSAMSVATTQVPTAAYWTIKSVVACMSHMISLIGFSHHEYASSTNDAWELSSLTHKVNSIHELLTKQLSLCYQYIEEKRHVEAYQTLVRLFETLHIDNIKILKALFYTKDDLPLVDGFTKRRVSIDVLRRKNVLLLISDLSIPQEELSILEQLYRESRNVPSRPESQYEVVWLPVVDRVTPWNEAMQQQFESMQLQMPWHTVYQPTAIDPAVIRYIKEVWHFVKKPLLVVLDPQGRVVCPNALPMMWIWGIAAFPFTSAREEALWKEESWRLELLVNGIDATLLTWIPEGRYICLYGGEDIGWIRKFTTMARAAARIAGVPLEMVYVGKSNPREKVRRVNEIIAAEKLSHYWPDLTSIWFFWVRLESMMYSKMQLGKTVEKDPILQEIMRMLGYDSTNQGWAIIARGATGIPELARGNGEMMLTSFAQFDEWKEHVEPKGFIPALNDHLQHIQTPHHCTHLKLPGNVGRIMDTVVCAECGRLMEKFFMYECCTE

**22061882_peptide|Acoerulea|Aquca_008_00082|Aquca_008_00082.1**

MDLVNPSRLEDISSDLVNKFLSELGPRQYSNDDLMRFTSNFLESNKIGFGGYGDVYKGQFPNGVLIAVKVLQKKDVVEDTFMAEVGTIGNALHRNLVKLYGYCFEHNIKALVYEYMENGSLDKILYENHHSSIEWVKLYGIVIGIANGLNYLHDGLEEQIIHYDIKASNILLDKNFTAKITDFGLAKLINRDVSVPLTRIRGTVGYNAPETWMPGSRVTFKCDVYSFGLMLFEVLGKRSNGMEENWLPELIWKQFKSGKLEHFIKDCGIAEKDKENANILCKVALWCVQFRPELRPSMKDVVLMLEKKLLVEQPPNPFMTFQISTPQVDLEQSHKENIERESSDSLELDPIPTYPQYTIASPSYASVADGKRHLKAYSLAHLLETIHIDNMKILRGLIYAHDDILPLYNGSTKKRVNLEVLRKKNVLLLISDLDIPYEEFTILYQMYSELRPKSSYEVVWLPVVDRSIPWTDEKQKQFERLQFMMPWYSVHHPSLLDPEVIDIKLVSKFATKTMLVMLDPQGRVVNQNAVHMMCIWGSFASPFTIMREEALWEEETWTLQLLVDGIDPNILNWIGENKLICLYGGENIDWIRKFTLAARAVSAAIRTPIEMVYVGKSNPGERVRKNNTIISAEKLSHFWPDLTSIWFFWVRLESMWNSKMQLGKTVENDPIVQEIMTMLSFDGIEEGWAVISKGSAEMTKAKGKTILTSFTDIEQWKELVEPKGFVNALTEYLHQLHT

**22631788_peptide|Mdomestica|MDP0000762971|MDP0000762971**

MALIPLNKGVNPTSRDGGRNRAPXLQDGRDGHRQYSTTADDNALINQIMLTDKSHDHPYEVPSLAVTQILQTIEAILSRFTKPDIHDGTLVPGALVPAAIHADSLEHEKALHANLSTLHDSFDVPNTVFNAIQCELMSKSLQREDANKTAMDILDIVQHYDWDEKVVLALGAFAIKDGEFWLLAQQYTTNPLAKAVGQLKQLHEILERAGTSLKPKFDAYNNLVRVIFKVTRTIVHLQEIHNDPHLSPEVKATAATAHIPTAVYWTIRAIIVAASQLLGITGSEPEYVTEAWELSSLAHKLENIFNHLEEILDRLHQLIQKNKDEDAFNAIARILETPHIDNSKPLKVLFYKDDQPALYDCYNKKRVDIDVLKRKVVILFITDLDVIHENEYMIVQQMYLEKQHNPTRPESQYEVVWLPITDVWTDAKYQQFEMLRNNIEWYTVFHPSVVSPIMVRYIRDPRKWNFAKKPLLVVMDPQGKIVHKNAVHMMCIWGSIAFPFTSQKERMLWEEETWRIELLADALDQNLYTWITDRKYICLYGGEDIEWIRNFTKAARNVAREAGIQLELLYVGKSKAKEKVVKNIISIIQAEKLSHTLEVNLIWFFWIRLESMWQSKGQILSEQSRTQTDHLKSDRIMQGIISMLSFGSSDRGWAVIALGSADMSKANGEHMLRSLREYNNWKIRPTEIGFTPALNEYLEGVHKQAPHHCTNLILPATGIMPETVACAECGRLMERFSMFRCCTD

**22635896_peptide|Mdomestica|MDP0000194799|MDP0000194799**

MASRQHQLTAPTQIQYSNRRDKRTSSSSLSDDSSLVRQIRDTHIPGRSDHSVNVNQILQVVEEIFHRASHGHAGVLLGTREHLETAEDKTTLSSADVVFHGLSYLIQKIYCEISCQCSGGPDAHASAIELLRTLSSYPWEAKVVLTLAAFSVCYGEFWLVAQLCTTDPLAKSVAILKQLSDIVEHAASVKPQLEAIDTLIKAILNVTKRIVEYGEMVKMQSRYVSEDTPPLSIALAHIPAATYWVIRGILASASHIAILTGSRREYVASTTEVWELSSLAHKLNNIHDHLSNELENCRRHIVTKRYDEEYENLRRLFQSLHLDNVKNLRALISHKDEFQPIQIGTLKSRFSLEVLRRKHVLLVITDLSLSNEEILVLDHIYKDQQKRPEIECEFVWLPIVDTNVWDEAKRFRFEDLKSRMQWYAVYDPLIIEPPVVKFVRNDWQFDKKMIVVALDPQGRVSSLNATHMLWIWGNVAYPFTDEREQVLWNAESWRLELVANGIDQSILEGIEGGRYICLYGSDDLDWIRKFTNRAKNVAKLAGISLDLVYVGRSTSTKERIRKVNKVIETENLSRYWPEYVSTWFFWSRMDSMRXSRAKQRKTLENDGILKEVMTLLSYDGSDQGWVMVWRGSNETARANGHLTLLTLDDFDAWKTAAADSGFVPTLKGELLRRHTPQHCTRLVIPGFGTDIPDKIACAECGREMEKFFMFRCCLDXXMFT

**22640323_peptide|Mdomestica|MDP0000272728|MDP0000272728**

MLGIAQTVVSKVASAVXTTAQHIEENVLKKLFLGTQVHVETIDEKPPKATFSSPLCTLKSIXCEMSCKPPGEEIAHKSTLAILNKLSTYSWEAKAVLALAAFALEYGEFWLLAQTQQSDLLAKSVAILKRVPVLLKPVDLQKRRQAVVELNVLIKTTLQVIECIFELEKISAYDPKDVPALAIAMDHIPVDVYWSIITIFSCATKITLLTSDEEKPYDLSQFAQKIHYILNKLKIQLLICKKQIVEAETYRKLRKLFRTXAEVMEVFKALIFTKDXVQPIIDGSTNKTVSIDVLRRKYVLLFISTLDISDDDISXIKPVYEGTKKDDKYKIVWIPIVEQWTDDLRKKFEVLMXKMPWYTVRYFAPVAGIRFIKEEWHFKGKPAVVVMNPQGKVENTNALHLIRIHGMKAFPFHKGIEDTXTNDKEWITPIVNDIHPTIQTWIXEEKYIFFYGGKDNDWIQQFTKKATTIANDPFIKELKINIELFCVGKSPKGGEDLGILGRFWNGIESLFFTKVNKQTDTVTKEVQKLLSYKNEGGWAVLTKGSTVVVSGHGFTILKVLDDFDTWKNFIKEKGFEFSFKAYYEKVIQTMRHCCRLDIPSVAGKVPETMKCPECPRTMETFVSYKCCHTDSPINAHH

**22649272_peptide|Mdomestica|MDP0000224533|MDP0000224533**

MASQQHLPAAAKQIRRDKRTSSAPQSDDSALVRQIRDTHTPGRFDSHSVDVNQILQVIEEIFHRTPHGHAGGLLGTPEHLDTVEDRTPLSSVDVLFHGLSYLIQKIYCEITCQCSGGADAHTSAIELLRTLSSYPWEAKVAITLAAYSVYYGEFWLVVQLCTTDSLAKSLATLKQLSDIVEHADSVKPQLEAINNLNKAILNVTKRIVEYSEMVNMQSQYVSEDKPPLSIALAHIPAAAYWVIRGILASASHIAILTGSRLEYVASTTEVWELSSLAHKLNNIHDHLTNELENCRQHIVAKRYDEEYETLRRLFQSLHLDNLRNLRALISHRDDIQPLQIGTTKSRSGLEVLRRRHVLLLITDLSLSNEXILILDHIYKDKQKRPEVEYEFVWIPMVDTNVWDEAKXLRFEDLKSRMQWYTVYDPXIIEPPVVKFIRNDWQFDKKMIVVALDPQGRVSSLNAIHMLWXWGNVAFPFTDEKEQQLWNAESWRLELVADGIDQSILEGIEKGTFVCLYGSDDLDWIRKFTNKAKNVAKLAGISLDLVYVGRSTATKERIRKVNKTIETENLSRCWPEYTSTWFFWSRMDSMRCSKAKHHKTLESDNILKEVMTLLSYDGSDQGWVMVWRGSNERARANGHLTLLTLDXFDAWKAAAAENGFVPTLQDXLGRRHTPQHCTRLVIPGFGPDIPDRIACAECGLEMEKFFMFRCCLD

**22654439_peptide|Mdomestica|MDP0000167324|MDP0000167324**

MLGIAQNVVSKVTSAVATTAQHIEGELSLFTMSDTKILELIYATHVHEDDSFDVDSLFLVTETIIKHSTQIVDSIVQGTQVHVETIDEKPPKATFSSPLCTLKSIGCEMSCKPPGEEIAHKSTLAILNKLSTYSWEAKAVLAFAAFALEYGEFWLLAQTQQSDLLAKSVAILKRVPVLLKPTDLQKRXQAXVELNVLIKTTLQVIECIFELEKLSAYDPKDVPALAIAMDHIPVDVYWSIITIFSCATKITLLTSDEEKPYDLSQFAQKIHYILNKLKIQLLICKKQIEEAETYRKLRKLFXTPAEVMEVFKALIFTKDTVQPIIDGSTNKTVSIDVLRRKYVLLFISTLDISDDDISIXKPVYEGTKKDDKYKIVWIPIVEQWTDDLRKKFEVLXAKMPWYTVQYFAPVAGXRFIKEEWHFKGKPAVVVMNPQGKVENTNAXHLIRIHGMKAFPFHKGIEDTXTNDKEWITPIVNDIHPTIQTWIXEEKYIFFYGGKDNDWIQQFTKKATTIANDPFIKELKINIELFCVGKSPKGGEDLGILGRFWNGIESLFFTKVNKQTDTVTKEVQKLLSYKNEGGWAVLTKGSTVVVSGHGFTILKVLDDFDTWKNFIKEKGFEFSFKAYYEKVIQTMRHCCRLDIPSVAGKVPETMKCPECPRTMETFVSYKCCHTDSPINAHH

**22655897_peptide|Mdomestica|MDP0000279658|MDP0000279658**

MALVPYNKAVNPTSRGAGHNRPPLLKQGRDGHRQFSTTADDSALVNQIMLTDKSHDHPYEVPPLAVTQILQTIEAILSRVTKPDIHDGILVPGALAPAATHAEHEKALHANLSTLHDSVDVPHSVFNAIHSASNNTNMPILLIDGKQLMCTFAQGEDANKTTMDILDIVQHYDWDEKAVLALGAFAIKDGEFWLVSQQYTTNPLARAVGQLKQLQEILERAGTILKPKFDAYNNLVGAIFKVTKTIVRLQEIHNDPHLSPEIKATAATAQIPTAVYWTIRAIVVAASQLVGITSSEPEYMTEAWELSSLAHKLENIFKHLEEILDGLYQLIQKNKEEEAFNAIARILETPHMDNSKPLRVLFYKDDQPALYDGYNKKRVDIDVLKRKVVILFITDLNVVHENENTIVQQMYLEKGHNPTRPESQYEVVWVPITDEWTEVKYQQFESLRNSMQWYTVFHPSVVSPIVVRYIRDPRKWNFAKKPLLVVMDPQGKIVHQNAVHMMCIWGSIAFPFTSQRERLLWEEETWRIELLADAIHPNLTTWITEGKYICLYGGEDIEWIRKFTKAARNVASDAGIQLEMLYVGRSKAKENVMNNIIKIIQAEKLSHTLDWNLIWFFWIRLESMWQSKGQLLSEQGKTQSRTDPLKSDPMMQGIISLLSFGSSDRGWAVISLGSAAMSKANGEHMLKSLSEFNDWKNRSSEVGFTPALNEHLEGVHRQAPHHCTSLILPATGIMPETVACAECGRVMERFSMFRCCLD

**22655900_peptide|Mdomestica|MDP0000500080|MDP0000500080**

MSSSSPSPLLPSPSTIITATATPPIITIAIIVDTTANHHHHHCRHYNPPLPPTTIISVIVTTTTYYHHRHYHHDNHHHPPSSPPPPTTSTTLANWLELKPIHIAIISLLPIPSQASLQPQPFGMALVPQNIAKPPTPAQSYNNPPIPAAQSYNNPPAPAQSYNNPPVPATQSYNNPPAPAAQNYNPAPIAAVQNYNPAPMLAAQNYNPAPLPAAQNYNPAPMLTAQNYNPALVPAAQSYNAPQAPGAQSYNAPQATGAQYYNKPTAGGRRGGDYYNRPLPKDGRRFSSTSDDSMLTNQILASDKSRARPYDVALLSLKHILQTVDVILSRVTQPDIHSTVTGALVTGAHADALEHEKASHAIMSGLPDNYDVPTSLFNAISCEIFCTWLSGEDANKTTMDILDIVQHYDWDEKVVLALGAFAVKDGEYWLVAHLYTTNPLAKAVGQLKQVQEILEHAGTSLKPKFDAYNNLVRAVLNVTKCIVQLHDLHHDPIMTTEAESAATTALIPTAVYWTIRSIVVAATQLLGITGMGPEHVTEAWELSSLAHKLETIHGHLKENLNRLLDIIKTKKEEQELSEIAYILESPHIDNTKPLRVLFYKDDQPALYDCYSKKRVDIDVLKRKVVILFLSDIDFINENEYMIVQQMYLEKRQNATRPESQYEVVWVPIVDTWTDAKYQQFEELRRSMEWFTVFHPSVVSKTVIRYMRKQDKWNYGKKPLLVVMDPQGKIVHTNAVHMMCVWGSIAFPFTSQRERLLWEEETWRIELLADSIDQNLITWITEGKYICLYGGEDIDWIRNFTKSAKNVAREAGIQLELLYVGKRNPKEKVVRNIMNVIQAEKLSHTLEWNLIWFFWMRLESMWQSKGHQMQSEAIRSGRLRTDNMKNDPVMQGIISMLSFGSSDRGWAVIGAGSSEMSKANGEHMHRSLSEYSLWNKRVDEIGFVRALNEYLTGVYKEAPHHCTSLILPATGLMPETVACAECGRLMERFTMFRCCTD

**22659747_peptide|Mdomestica|MDP0000243954|MDP0000243954**

MYKPKRVSEEGLNAWDNVYGVIKTLNXAARESIYLXCFSYMPLTMQCKPPSEEVAHNTTLAILNKLSSYSWEAKAVLTLAAFAMEYGEFWLLAQLQESDRLAKSIAILKRVPVLLKPSDLHKKRQAVLELNNLIKATLQVIECIDQFDKLSSYDPKDVPALALAMDHIPVDVYWAVATVVACATKITILTCNEDKEHDLAPFAQKIHYVLNKLKIQLIVCRKQIEEAETYRRLRKIFXTPTEIMEVFKALIFTKENVQPLVDGSTKQMVKIDILRKKNVLLFISSLDISDDDISILKPIYDMIKKDNQHKIVWIPIVEHWTDDRRKKFESLRNKMPWYTVQISAPVAGIRFIKEEWXFKGKPTLVVMNPQGKVEHPNALHMIRVWGVNAFPFTKATEEELSHGHGDKWIGTVVQGVSQSVTIFFYGGKDNGWIQEFTKKATALANDPIFKEAKIXIELFCVGKGSKGEDDHGILGKFWTGIESLFFTKVHXPADQVGQEXQKLLSYKNESGWAVLSKGHSVVLTGHGVSILRVVEDFDKWKDHVKEXGFEFCFKSYHERVRTVSRPCCRLDIPGSTGKVPDTMKCPDCHRSMETFISYKCCHIDGPTAHH

**22664555_peptide|Mdomestica|MDP0000901389|MDP0000901389**

MALVPQNFPKAPAPAQNYNNPPLPAAQNYNNPPAAPAARNYDQAPVPAAQNYTQTPIPAAQNYNPALVPAAQNYTAPDAPGAQNYNAPRAPAGQYYNNPTAGGRRGGDHYNRPLLKTGRRQSSITSDDSMLTNQILASDKSHARPYDVTLLSLKHILQTVDVILSHVTQPDIHGIFTGAPVPGAHADALEHDKALYATMSGVHDNYDVPTSLFNEISCEMFCKWQSGEDANKITMDILDIVQHYDWDEKVVLALGAFAVKDGEYWLVAQLYTANPLAKAVGQLKQVQEILERAGTSLKTKFDAYNNLVRAVLNVTKCIVQLHDLHRDPHLTTEAKSAANTALIPTAVYWTVRSIVVAGSQLLGITGMGPEYVTETWELSSLAHKLENIHSHLKDNLDRLQDIIQRKKDDEALAAIAYILETPHIDNTKPLRVLFYKDDLPALYDCYTKKRVDIDVLKRKVVILFLSDLDVVHENEYIIVQQMYMEKRHNPTRPESQYEVVWVPIVDMWTDAKYQQFEEVRRNMEWFTVFHPSVVSPTVIRYIRKQDKWNYVKKPLLVVMDPQGKIVHTNAVHMMCVFGSAAFPFTSNREKLLWDEETWRMELLADSLDQNLITWITEGKYICLYGGEDIDWIRNFTKSAKKMAQEAGIQLELLYVGRSKPKEKVVRNIMNIIQAEKLSHTLEWNLIWFFWLRLESMWQSKGQQIQSEAFRSGQFRTDNIKNDAVMQGIISMLSFGSSDRGWAVIGAGSSDMSKANGEHMHRSLREFNLWNRRVSEIGFVPALNEYLAGVYKEAPHHCTSLILPATGLMPETVACAECGRLMERFTMFRCCTD

**22674501_peptide|Mdomestica|MDP0000220190|MDP0000220190**

MLGLANNVAAKVASVVTTTHQHITGDHELSLFTMSDQKILEQIYGTHVHADESFDDDSLFGITENILKRATQIVDKIVQGTQVHVENIEENTPKAGFSAPLCTLKSIASEMQCKPPSEEVAHNTTLAILNKLSSYSWEAKAVLTLAAFAMEYGEFWLLAQLQESDRLAKSIAILKRVPXLLKPSDLHKKRQAVLELNNLIKATLQVIECIDQFDKLSSYDPKDVPALALAMDHIPVDVYWAVATVVACATKITILTCNEDKEHDLAPFAQKIHYVLNKLKIQLIVCRKQIEEAETYRRLRKIFXTPTEIMEVFKALIFTKENVQPLVDGSTKQMVKIDILRKKNVLLFISSLDISDDDISILKPIYDMIKKDNQHKIVWIPIVEHWTDDRRKKFESLRNKMPWYTVQISAPVAGIRFIKEEWNFKGKPTLVVMNPQGKVEHPNALHMIRVWGVNAFPFTKATEEELSHGHGDKWIGTVVQGVSQSVTIFFYGGKDNGWIQEFTKKATALANDPIFKEAKIXIELFCVGKGSKGEDDHGILGKFWTGIESLFFTKVHXPADQVGQEXQKLLSYKNESGWAVLSKGHSVVLTGHGVSILRVVEDFDKWKDHVKEXGFEFCFKSYHERVRTVSRPCCRLDIPGSTGKVPDTMKCPDCHRSMETFISYKCCHIDGPTAHH

**22678679_peptide|Mdomestica|MDP0000212614|MDP0000212614**

MGTQVHVETIDEKPPKATFSSPLCTLKSIGCEMSCKPPGEEIAHKSTLAILNKLSTYSWEAKAVLAFAAFALEYGEFWLLAQTQQSDLLAKSVAILKRVPVLLKPTDLQKRXQAXVELNVLIKTTLQVIECIFELEKLSAYDPKDVPALAIAMDHIPVDVYWSIITIFSCATKITLLTSDEEKPYDLSQFAQKIHYILNKLKIQLLICKKQIEEAETYRKLRKLFQTPAEVMEVFKALIFTKDTVQPIIDGSTNKTVSIDVLRRKYVLLFISTLDISDDDISIIKPVYEGTKKDDKYKIVWIPIVEQWTDDLRKKFEVLRAKMPWYTVQYFAPVAGVRFIKEEWHFKGKPAVVVMNPQGKVENTNAIHLIRIHGMKAFPFHKGIEDTLTNDKEWITPIVNDIHPTIQTWIKEEKYIFFYGGKDNDWIQQFTKKATTIANDPFIKELKINIELFCVGKSPKGGEDLGILGRFWNGIESLFFTKVNKQTDTVTKEVQKLLSYKNEGGWAVLTKGSTVVVSGHGFTILKVLDDFDTWKNFIKEKGFEFSFKAYYEKVIQTMRHCCRLDIPSVAGKVPETMKCPECPRTMETFVSYKCCHTDSPINAHH

**22687483_peptide|Brapa_Chifu-401_v1.2|Bra040547|Bra040547**

MESLIKSQHAQKQAGHSSKTSGTEIIPATTGLTMSSDESMMLKLIQQTHSPDAREVQVRGLLSLVEDILDRATLDSDDSNASMLPLPTDDKLMQSSMMSVLDSVSYAIDRVACEIAYKSLTGSDAHEITMSVFEHLSSFHWDGKLVLTLAAFALNYGEFWLLVQFYSKNQLAKSLAMLKLVPVQNRVTLESVSQGLNDLLREMKSVTACVVELSELPGRYITLDDPQLSRIISTIPIAVYWTIRSIVACISQINMITAMGHEMMNTQMDSWETSMLANKLKNIHDHLAETLRLCYRHIEKQRSSESLKMLHSLFDTTHIDNMKILTALIHPKNHTMPLQDGLTKRKVHLDVLRRKTVLLLISDLNILQDELSIFEQIYTESRSSLLGADGKSHMPYEVVWVPIVDPIEDYERSPSLQKKFEALRAPMPWYTVDSPKLVERHVGEFMRERGHFMKKPILVVLDPQGNEASLNALHMIWIWGTEAFPFTRAREEELWRRETFTLNLIVDGIDAIIFNWINPGNYIFLYGGDDLDWIRRFTMAAKATAKDSNVKLEMAYVGKRNHSHREQIRRISEAVRAENLSHSWAEPALMWFFWARLESMLYSKIQLGKSDDHDEVMQGIKKILSYDKLGGWALLSKGAEIVMITHGVIERTVTVYDRTWKTHVPTKGYSKAMYDHHHDEVLRETGHPCAHFDFHITARSGRIPEKMNCFECHRPMEKYMSFACCHDEKLLDQDENYNF

**22687490_peptide|Brapa_Chifu-401_v1.2|Bra040546|Bra040546**

MAKRFQLNPKPFSTDPPADPLKRVSLIPRSAEQKLTDNQGERRPVAAKTHEDGEQSDGHHVAPPLKTVEPTKVMDHETEKKGSITPNTAHHPHSSEDIVDANSRHSLVPRSLGDNSLGGRFGPGKKQAFHRNGRPMFSLSDDRVMADRVLKTHSPDMIFFDVKSLLSVVDDIFKSHVPSIDSSAPKPSLVFKDYADHVSFETFADLIDQISCEIECKCLHGGESSHGMKTTGLHLDSRNTTTFSVLSMVSKYRWDAKLVLILAALAVKYGVFLLLAETYATNQLTKSLALIKQLPSIFSRQNALHQRLDKTRVLMQDMVDLTTTIIHIHQLPPHHITPAFTDHVPTAVYWIVRCVLICASHISGASGFKQDQIMSFMEVSEIHENSERLRKINAYLLEQLKKSHLTIEDGIMEEEYQELIQTFTTIIHVDVVPPLIRLLRPIDFLYHGDGASKRRVGINVLTQKHVLLLVSDLENIEKELYILESLYTEAWQQSFEILWVPVQDFWTEADDAKFESLHSSMKWYVLGEPRRLRRSAVRFMRQWWGFKNRPILIALDPKGQVMSTNAFPMVWIWQPFAHPFTTSRERDLWGEQEWNLEFLIDGTDPHSLNQLVEGKYICLYGGEDMQWIRNFTSLWRSVAKAANIQLEMVYVGKRNPKKGIQPIINTIREDNLSHTLPDLFQIWFFWTRIESMWESKQRMLKAHGTKEKDLVLQEIVAMLGFGGEGDGWGLVSKTSDLMVRAKGNLFSNALAEYNEWEVNIPAHGFLKALNDHLLMRIPPHHCTRFMLPETAGIIPNEVECTECRRTMEKYYLYQCCLE

**22691749_peptide|Brapa_Chifu-401_v1.2|Bra040700|Bra040700**

MESLIKSQHAQQLAGHSSRDGKTPRTTEMVPATSTGGLTMSSDESMMLKLIQQTHSPDAREVQVRGLLSLVEDILDRATLDSDDSNASMLPLPTEDKLMQSSMMSVLDSVSYAIDRVACEIAYKSLTGSDAHEITMSVFEHLSSFHWHGKLVLTLAAFALNYGEFWLLVQFHSKNQLAKSLAMLKLVPVQNRVTLESVSHGLNDLIREMKSVTACVVELSELPDRYITLDDPHLSRIISTIPIAVYWTIRSIVACISQINMITAMGHEMMNTQMDSWETSMLANKLKNIHDHLAETLRLCYRHIEKQRSSESLKMLHSLFDTTHIDNMKILTALVHPKNHTTPLQDGSTKRKVHLDVLRRKTVLLLISDLNILEDELSIFEQIYTESRRNLQGIDGKSHMPYEVVWVPIVDPIEDYERYPSLQNKFEALREPMPWYTVDSPKLIERHVVEFMRERWHFMNKPILVVLDPQGNEASLNALHMIWIWGTEAFPFTRAREEELWRRETLTLNLIVDGIDSVIFNWINPENYIFLYGGDDLDWIRRFTMAAKATAKDSNVKLEMAYVGKRNHSHREHIRRISEAVRAENLSHSWAEPALMWFFWTRLESMLYSKIQLGKADDHDEVMQGIKKILSYDKLGGWALLSKGPEIVMITHGAIERTVTVYDRTWKTHVPTKGYTKAMYDHHHDEILRETGKPCSHFDFHITARSGRIPEKMNCFDCHRPMEKYMSFACCHDDKLLDQDENYNF

**22710442_peptide|Brapa_Chifu-401_v1.2|Bra000996|Bra000996**

MAQRFQLNPKPFTDPPADHLNRVSLIPRSAEQKLAADNLGDRRPLAPRTHEDKPFGEHSDALHHHNVAPPPHNKVMDHDPEKKTGSVVPKTPHHPHPSEDLHDANSRHSLVPRSLGHNSLGGRFGPGKNQAFRRNGRPMFSLSDDRVMADRVLKTHSPDMVFFDVKSLLSVADDIFKSYVPSIDSSSSASKPSVVFKDYADHTSFETFAELIDQITCEIECKCLHGGESHGMMTSGIHLDSRNTTTFSVLSLVSKYRWDAKLVLVLAALAVKYGVFLLLAETYATNQLTKSLALIKQLPSIFSRQNALHQRLDKTRVLMQDMVDLTTTIIRIYELPPHHITAAFTDHIPTAVYWIVRSVLICVSHISGASGFKQDQVMSFMEVSEIHENSERLRKINAYLLEQLNKSHLTIEEGIVEEEYQELIQTFTTIIHVDVVPPLLRLLRPIDFLYHGAGDSKRRVGINVLTQKHVLLLVSDLENIEKELYILESLYTEAWQQSFEILWVPVQDIWTDAHDAKFESLHSNMRWYVLGEPRKLRRAAVRFVREWWGFKNRPILVALDPKGQVMSTNAFPMVWIWQTFAYPFTTAREHDLWSEQEWNLEFLIDGTDPHSLNQLVDGKYICVYGGEDMQWIRNFTSLWRTVAKAANIQIEMVYVGKRNPKNGIQPIINTIRDENLSHTLPDLFQIWFFWARVESMWESKQRMLKAQRTKGGRQGFKEEEEKDLVLQEIVALLGFGGEGDGWGLVSKTADLMVRAKGNLFSQGLAEFNEWEVNIPAHGFLKALSDHLMMRLPPHHCTRFMLPETSGIIPDEVECTECRRTMEKYYLYQCCLE

**23021358_peptide|Mtruncatula|Medtr7g032660|Medtr7g032660.1**

MTSIGKLGAMQQLMKGGKMMPANVISDDTILVKKIVADHNPEGLDYDVKPLLHIVEDILRRSTLGSSEHDSMGELSHVDQLEDRTHLPSYTSMLEALSVKIDRISCEISYKILSGVDAHSTTVAIFEMLTIYKWDVKLVLALAAFALNYGEFWLLAHIHDTNQLAKSMAILKQLPGIMEHSSSLKPRFDTLNDLVNVILEVTKCVIEFNDLPIQYIRQDVSAYNTVSNHIPVAAYWSIRSIVACAAQITSLTTLGYEIFTSNDAWELSTLAFKLRSIVDHLRKQLDICKIHVEEAMDTEAYGMLRELFLTPHTDNMKIIKALIYSHDDILPLYDGVSKKRASLEALRRKNVLLLFSGLEFSTDELLILEQIYNESKAHAPRQDNRYELVWIPIVDQTSEWTDQKQMQFENLRESMPWFSVYHPSLISKAVVWFIQSEWKYKNKPILVVLDPQGRVACPNAIHMMWIWGSAAFPFTSFKEETLWKDETWRLELLVDGIDSEILNWIKEGKYIFLYGGDDPEWIKRFVKEARKVAQATRTPLEMVYVGKSNKRDQVQKVCDTIIREKLYTHSWSEQSMIWFFWTRLQSMLFSKIQLKQVDDNDRVMQEIKKLLSYDKQGGWIVLAKGSQIVVNGHASTGLQSLIEYDLMWKEHAERDGFETAFKEHYGKLHAVDNPCCRFEFSHSMGRIPDRLTCPECRRNMHVLTTFQCCHDDNVEEDFFVSSVTPPNTN

**23042197_peptide|Mtruncatula|Medtr1g074990|Medtr1g074990.1**

MVSLVRSLIHIGNDNAIDHNPLTMSDEHILEEIYVTHVHSDTKFDAESLFNIAGNILTRSTHVVDNVLQGHQGGLEHLDNINPPASFTSPLCTLKQINSEMSCKAPGEEIAYKTTLAILKKLSNYSWVAKGVLTLSAFSLEYGEFWLLSQNLPTEPLAKSLGIIKRVPQLSKPEALKKHRNEILELNNLIKATWQVIEIIIELERLNSRHDIKEVPALAPALEQFPVDVYWVIITIVAIVTQFECLTTNSDKRQDLSHFGQKINIIISKLRKHVSQCTIQIDEAEYNKLLRKLFQTPTEIMEVFKVLVFWKDTPKAPIYDGSTKTLVNIEVLKKKDVFLFISTLDISQEDISILIPIYDHIKKTGSQHKIVWVPIVEEWNDKLKKKFDSLKSKMPWYVLHHFAPIKGIKYIKEELHFKQKPLFVVLSPQGKILHHNAFHMIQVWGVKGFPYSKSKEESMTQELMWVDSLLADIDIKIKWKEEKSVIIYGGKDKEWIQQFTKYAGALANDAAIKQTKTSIDLFCLESQQPNVVNNFWKKVESLFVTKMHEKTNTVTQQVEKLLSYKNETGWAIVTKGSIVTSVGHGTSVLKTVSEFDKWKDVAINKGFEFAFREHHHKVASTVHICSHLEIPNVAGKIPDFIECPDCHRTMEVFISYKCCHNGDKVNAEH

**23042339_peptide|Mtruncatula|Medtr1g075030|Medtr1g075030.1**

MIRLSRSSIHIGNGDIIDHNPLTMSDEHILEEIYSTHVHSDTKFDAEYLFNIAGNILTRSTHVVDNFVQGHEQQTSLEQLDNINPPASFTSPLCTLKKINSEMACKAPGEEIAYRTTLAILNKLSNYSWVAKGVLTLSAFALEYGEFWLLSQYLPTEPLAKSLAIMKRVPQLTKPELLKKHRYAVLEVNNLIKATSQLIDIIIALERLNSRHDIKEVPALAPALEQFPVDVYWVIITIVAIVTQIECLTTDSEERQDLSQFGQKINIIISKLRKHVSQITIQIDEAEYNKLLKKLFQTPTEIMEVFKVLIFWKDTPQTPIYCGSTKTLVNIDVLKKKDVFLFISTLDICQEDISTMIRIYDHIQKTGSQHQIVWIPIVEEWNDRGRKKFDSLKSKMPWYVLHHFATIKGIRFIKEELHFKLNPLVVVLSTQGKILHQNAFHMIHVWGVKGFPFTKTKEESMTQELMWVDSVLVGIDIKIKWREDDIVIICGGKDKEWIQQFTKYFGALVNDATIKQTNTSIELICLESQQQNVVNTFWKKVESLFVTKMHEKTNSVTQQVEKLLSYKNESGWAIVTKGSIVIAVGHGTTVLKTFAEFGTWKGDVSTKGFEYSFREYHNTIASSVHICSHLEIPNVDGKIPDFIKCPDCHRTMEVYISYKCCHNG

**23043148_peptide|Mtruncatula|Medtr1g075230|Medtr1g075230.1**

MADLMKSFLHITSGNIIHNNPLIMSDIQIMGEIYSTHVHSDTKFDVESLFNIAANILRRSTYIVENVVQGNQGGLEPLDNTHPPASFTPPLCILKKINSEMSCKAPGEKIAYETTLTILKKLSNNLWVEKGVLTLSAFAIEYGEFWNLSQHLPTEPLAKSLAIMKRVPQLTKTEALKKHRNEILELNNLIKATWKVIEIIIELERLNSLHDIKKVPALALALEEFPVDVFWVIITIVTIVTQIECLTTDSIRKLFELPDEAEYIKLLKKLFQTPTEVMEVFKVLIFRKDAPKESIYDGSTKTLVDIEVLKKKEVFLFISTLNISQEDISILIPIYDHLKETGSQYKIVWIPVVDEWTDKLRKKFDSLKSKMPWYVLHHFAPIKGIKYIKEELHFNQKPLVVVLSPQGKILHHNAFHMIQVWGVKGFPYTEDKEKSITQELKWVVSLLVDIDIQIKWEEEKFVIIYGGKDKAWIQEFTKFATALANDTNIKQAKTSIELLSLESQKPNVVNKFWTKVESLFLTKMNNNDTTDSVKQQVEKLLSYKNETGWAIVTKGSIVIAVGHGTTVSKTVSEFDKWKEVAIKKGFEHAFNEHHKKVAPSFHLCSHLEIHKVAGKIPDFVECPDCRRRMEVFITYKCCHDEDKTY

**23043845_peptide|Mtruncatula|Medtr1g075170|Medtr1g075170.1**

MSTALSYNVPISGTTTQKNDTSQQQKSQLPNPFKLEDIEILNKVYLTHVNDNMKYDRDTLFNLVSNIISASTQTSGTNSGLNTQISFKPDFSVLKRISCQMITTRGTAECAHQTTMWVLHHLRGFSWEAKALITLAAFSLEYGAIMHLHRIQSSDTLGNSLKQLSQVQFRKVPADITELVTFLLQVLQDIKTWAAWSAFGYDLDDVNSLPDAMQWIPLVVYWTVATIVACTGNLVGISEHKLSDYVKSLSDVVKELRRHLKSCELEIGKIHENENLLKDSDNIKDVVAFLRLLIKGNGTDQIPPIFIGNDQVKTGIEVFKKKHVLLFVSGLDTLRDEILLLNSIYKRLQDKPQEVLKGSFKKEDFKILWIPIVNKWDEDRKKEFKNLKESMKWYVLEHFSELPGRGIIKKKLNYDIGYPPILAVINPQGDIINKDAMEIIFQWGIDAFPFRISDAEDIFKKWEWFWKLMKKVDVNIEKMSWDRYIFIYGGNDPKWIQDFTRAIGSIKKHQTIQNVDVNIDYHQLGKNNPTEIPYFWMGIDGRKQQNKTCKDSVDCEIQTAVKKLLCLKQDPLGWVLLSRGRHVTVFGHGEPMYQTVADFDKWKNNVVEKESFDEAFKEYYDTKLSEISSSASCAVNSSDVLATITCPNPFCGRVMEVTSVNYKCCHRDDPDSCCI

**23044977_peptide|Mtruncatula|Medtr1g075180|Medtr1g075180.1**

MSLSNGTKLPNPFDLDESQILDKVYLTHLHDDDKCDKDVLFHILSNVILRTRLAESRAEFEPEFRTLKLISCQMITTPRGERYVHQTTMWILQQLKTYSWDAKALIALAAFTLEYGNLLYLTETSTSSDQLVNSLKILNQIQNRKVTVPATDLVELIMDVLLHIHEWATRSGVGYNTLDVPSLSDALQDIPVAVYWIIASTVAATGNIIGVSDYTLSDFKEKLNFVDSKLKEHLKLSKWQIDSVEEYLKRKKAISNPKDIIDFLKLLIQRNGDNLLIYDGTTKNKTDIEVFKDKYVLLFISSLNKVDDEILLLNSIHDRLQDNPQVIKGYKKEDFKILWIPIWDVDDQKIKFDSLKNKIRFYAVDYFSELPGIRLIREHLNYSDKPIVPVLSPLGEKMNDDAMDLIFQWGIDALPFRKQDGYDLTQKWKWFWDVTKRVNLGIQVKGDRYIFIYGGSDKKWIQDFTLALEKTKRHETILRADAIIEHYHLGKDDPKIVPRFWIEIESKRLKKHQDGIDCEIQDIVKSLLCLKQDPQGWVILTKGYNVKLLGHGEPMYQTLADFDIWKDRVLQKEGFDIAFKEYYDTKVKDTYVKQPCEIINVDNNINGNVIATISCPNPTCGRVMEVSSVNYKCCHRDDAAAPQNGKI

**23045283_peptide|Mtruncatula|Medtr1g074950|Medtr1g074950.1**

MATIIKGTLKTLLSAGEIESNPLTMSDDQILDQIYSTHVHSDTKFDAASLFTLAQNTLARSTHIVDSVVQGTKVSLEQADDKSLIPNFSSPLCTLKSISSEMSCKPPSEEIAHKTTLAILNKLSHYDWEAKAVLTLSAFALEFGEFWLLEQHLSTDPLAKSVALLKRVPILAKPAAIQKHRQAITELNSLVKITLQVIEFILELDYLNDRYDTKVVPALELAYEQIPVDVYWTIITIAAIVTQLDCLITESEHKQELSHYGQKINIILSRLRKQITVCRQQIDTAKYIQELKKLLQTPTEITVVLSFLIFPKDVPQLLYDGATKTTVDINVVLKKKNVYLFVSTLDVTEEEITAVRSVYESIKTNEQYKIVWIPIVEGWNEQLRKKFDILRSKMPWYVVQNVENIAGFKFINEEWDFKKKSMFVVFSPQGKVQHKNAFHLIKSYGIKAFPFTMDDEIRIQKDRNWIVSVVGNIDRNISIWTEQNKHIFFYGGHDKEWIQQFTKYATALANDATIKEAKISIELFYVDKEDKNLVSRFWSGIESLFVTKIHKTTDVVTQEVQKMLSYKNETGWALLSKGPSVVLSGHGTTILKTVAEFEKWKDVVIKKGFEFAFTEYHTNVARVTHRCSHLEIPIVAGKLPETIKCPDCPSTMEIFISYKCCHNKTNANGKH

**23139345_peptide|Lusitatissimum|Lus10022306.g|Lus10022306**

MHTSENPSPLSPPSLLTMAFRGLPPPTSHIPSKQLLARGGEHHGSNMLMMSDENVMMKQIVATHLPDGREVDVRPLLVLVEDILSRATLQVDTNIHTPIQARTEIGDKANQINQVTMMEALSYTIDRVSKEIAYKSLGGADGHSTTVMILESLASFPWDAKLVLTLASFALHYGEFWLLAQIYPSNQLAKAMATLRQLPMIMEHSGPLKPRFDALNKLIKAMLEVTRCLVQFKELPPTYISDDVPALSTALTHVPTAVYWTIRSIVASANQITLITTMGHEYALSTTEAWELSSLEHKLNNICDHLRQQLGSCYHHIDDRKNSEAFRMLVDLFEMTHIDNMKILKALIHAKDDLMPIVDGSTKRKVSLEVLRRKNVLLLISDLNISQDELAILEQIYNESRLHMTRMESQYEVIWIPVMDTSIQWTEAMHKQYESLQSTMPWYTVSHPALIENAVIRFIREKWHFRNKAILVVLDPQGRILSPNAIHMMWIWGSNAFPFTSMREESLWRDETLRLELLVNGIDPMILNWIKEGKYIFLYGGGDVEWVRKFTNTARAVAVASRIPLEMAYVGKSTKKELVRRVIATITVEKLSYFWQDPTMMWFFWTRLESMLFSKIQLGKNDDHDPMMLEIKKLLSYDKEGGWAVLSRGSNIISNGHGNTVLTTLVEYDLWKEQIPVKGFDLAYDDHHKKVHGVTYPCCRFEFPNTAGRIPETMKCPECHRHMEKHTTFLCCHDEQGHSSSSSF

**23139458_peptide|Lusitatissimum|Lus10022305.g|Lus10022305**

MSLRSLAAGTGANMQLIKNDRGGMLSMSDDNVMMKQVQATHAPDGREVDVRPLLNLVEDILSRATLQVDTPLLANKAHAELEDKNHQMNYVTMLDALSYTIDRISCEIAYKALGGGDAHGTTMTICETLTNYQWDAKLVLTLAAFALHYGEFWLLAQIYSSNQLAKSMAILKQLPSIMEHSGPLKPRFDALNKLIKAILEVTRCVVHFKELPPMYISHDVPALSTAMSHIPTAVYWTIRGIMAFANQITLLTTMGQQYALSTTESWELSTLEHKLANICEHLRMQLATCQQHIDEKRSVEEYKMLVDLFEMIHIDNMKVLKALIYPKDDIQPLVDGSTKRRVNIDVLRRKNVLLLISDLNISNDELSILEQIYNESRLHATRMDNQYEVVWIPLVDPSIKWNDQMQNKFESIQSTMPWYSVYHPSLIHKAVINFVRESWHFRNKPILVVLDPQGKVVSPNAIHMMWIWGSNAFPFTSRREEDLWSTEAWKLELLVNGIDPMILNWIKEEKYIFIYGGDDVEWVRKFTNSARAVATAARIPLEMVYVGKSSKREQIRRVIATITVEKLSYFWQDLTMIWFFWTRLESMLFSKIQLGKVDDHDPMMQEIKKLLSYDKEGGWAILSKGSNIISNGHGNTVLTTLVEYDLWKENIPVKGFDLSFDEHHKKIHGVTYPCCRFEFPNTAGRIPEMMRCPECHRHMEKYTTFLCCHDDGTHGSLF

**23159340_peptide|Lusitatissimum|Lus10021828.g|Lus10021828**

MTLVPHKMSRGDRHMFASSDDNAMMKQIQATHAPDGREFAVEPLLHIVEDAFHRSHPSLPPTSGELAGAHQAQLDALEDKALENGLHETLEILSFTINKISCEISCKCSGGGDAHATTMSIFNIVSSYSWDAKVVLALAAFAVNYGEFWLVAQLYLTNPLAKAVALLKQLPDILERAETLKLRFDALSNLIKAMMDVAKCIVEFKELPSQYISPDTPEMLAATAHIPTAVYWTIRSIVACASQIIGLIGMGHEYITSTTEAWELSSMAHKVTNIHSHLMKQLTVCYQHIDEKRHIEAYQALIRLFEMVHIDNLKILRALIYAKDDQLPLVDGATRKRASLDVLRRKNVLLLISDLELSHEELSMLEQMYHEARRHPTRSESQYEVVWMPVVDRSTPWNEEKQKGFERLQSMMPWYSVYHPSLLDLAVIRYIKEAWHFNKKPLLVVLDPQGKVVNPNALHMMWIWGSLAFPFTTMREEALWKDESWRLELLADAIDPMIPSWISQGKFICLYGGEDMEWIRKFTVLAQAVAQAAGISLELLYVGKSSPREKVRKNNVMIQSENLSHVLPDLTLIWFFWVRLESMWHSKVQHNRTVENDVIMQEIVTMLSFDGSEQGWAVVSRASAEMAKGKGETILKSLMEYEEWIGIAEEKGFVPALNDYLLGLHSPLHCNRLILPGSTGSIPDRVVCAECGRAMEKFIMYRCCTD

**23161222_peptide|Lusitatissimum|Lus10032103.g|Lus10032103**

MAFRGLPPPKATSSSLVPGAGRPSQLMSRGPGDQQPHGGHGNMLMMSDDNVMMKQIAATHTPDGREVDVQPLLVLVEDILNRATMQVDTTLRPTKAVTEIEDKANQINQVTMLDALSYVIDRVSKEIAYKALGGSDAHSTTVTIFETLSSYPWDAKLVLTLAAFALNYGEFWLLAQIYSSNPLAKAMATLRQLPTIMEHSGPLKPRFDALNKLIRAMVEVTRCVVQFKELPHGYISQDVPALSTALAHVPTAVYWTIRSIVACANQITLITTMGHEYALSTTEAWELSSLEHKLNNIYEHLRSQLGSCYQHIDERKNMEAFKMLVDLFETTHIDNMKILKALIHAKGDPAPIVDGSTKRKVSLEVLRRKNVLLLITDLNISHDELSILDQIYTESRHHTGRMESQYEVIWIPVVDPSIQWTDAMQNKFESLQQAMPWYTVDLPSLVEKAVIRFIREKWHFRNKAILVVLDPQGRVVSPNAIHMMWIWGSNAFPFTSLREQSLWGEETWRLELLVDGIDPMILNWIKDGKYIFLYGGDDVEWARKFTNTARAVAVASRIPLEMAYVGKSTKKELVRRVIATITVEKLSYFWQDPTMIWFFWTRLESMLFSKIQLGTIDDHDPMMLEIKKLLSYDKEGGWAVLSKGSNIISNGHGNTVLTTLVEYDLWKEHIPAKGFDLAYDDHHKKVHGVTYPCCRFEFPNTAGRIPETMKCPECHRRMEKYSTFLCCHDHEGH

**23161688_peptide|Lusitatissimum|Lus10000551.g|Lus10000551**

MSFRLTPARMQRKFKGDRLIFTNSDDAAMMKQLESTHYPDGVEFDVKPILLIVEDIFLHANNPTLASTVSPGTEGHVEALLADQTLQASTMELLESLAFVIDRVASEMSCTCFGGKDPHGSAMAMLKMLANFSWDAKLVIVLAAFAMNYGEFWLIAQNYTSNQLAKAVAILNQLPEILEKSNSQMPLFDAVRNLIKPMLDIGKCIIKLKELPSEFITSDVPALSMAMSHIPIAVYWIVRSIVACASQMTGLIGQGHGDFEEVSDQIVGCRHLVATTEAWELSSLDEKKFLQSYQNLIHLSETMQIDNMKILKALIYAKDDLQPLVDGASKKRLMVSQWQFHLDVLRKKNVLLLISSLDILQEELAILEQIYNESRVDPTRRDVQYEVVWLPIVDPAIPWTEMKQKQFESLQATMHWYSVHHPSIINHAAIKFIKEVWNFGEKTILVVLDPQGQVASPNALHMMWIWGSAAFPFTTSREESLWKEETWRLELLVDGIDPMILQWMSEDKYICLYGGEDMEWIHKFTSVLRAAANAAGISLEMAYVGKSNPKERVRKNISMITADNLSYCWQELTSIWYFWIRIESMWHSKNQLQRTVDNDPIMQEIMTFLSYDGSEDGWAILCRGSGEMVRAKGSTFVTCLTDFTSWESQVQLMGFLPALKQHLMELHTPHHCNRLVLPGTSGTAPERVVCSECGRSMERFIMYQCCDE

**23161691_peptide|Lusitatissimum|Lus10000548.g|Lus10000548**

MQLIKNDRGGMLSMSDDNVMMKQVQATHAPDGREVDVGPLLNLVEDILSRATLQVDTPLLASKSHAELEDKNHQINYVTMLDALSYTIDRISCEIAYKALEGGDAHGTTMTICETLTNYQWDAKLVLTLAAFALHYGEFWLLAQIYSSNQLAKSMAILKQLPSIMEHSGPLKPRFDTLNKLMKAILEVTRCVVHFKELPPMYISHDIPALSTAMTHIPTAVYWTIRGIVACANQITLLTTMGQQYALSTTESWELSTLEHKLANICEHLRMQLATCQQHIDEKRSVEEYKMLVDLFEMIHIDNMKVLKALIYPKDDIQPLVDGSTKRRVNIDVLRRKNVLLLISDLNISNDELSILEQIYNESRLHATRMDNQYEVVWIPLVDPSIKWNDQIQNKFESIQSTMPWYSVYHPSLIHKAVINFVRESWHFRNKPILVVLDPQGKVVSPNAIHMMWIWGSNAFPFTSRREEDLWSTEAWKLELLVNGIDPMILNWIKEEKYIFIYGGDDVEWVRKFTNSAQAVATATRIPLEMVYVGKSSKREQIRRVIATITVEKLSYFWQDLTMIWFFWTRLESMLFSKIQLGKVDDHDPMMQEIKKLLSYDKEGGWAILSKGSNIISNGHGNTVLTTLVEYDFHSFPMLP

**23162807_peptide|Lusitatissimum|Lus10034567.g|Lus10034567**

MALVPHKMSKGERHMFASSDDNAMMKQIQATHAPDGREFAVKPLLHIVEDAFRRSHPSLPPTSGEVTGVHQAQLDALEDKALENGLHETLEILSFTINKISCEISCKCSGGGDAHATTMSIFNIVSSYSWDAKVVLALAAFAVNYGEFWLVAQLYLTNPLAKAVALLKQLPDILERAETLKPRFDALSNLIKAMMDVAKCIVEFKELPSQYISSDTPEMLAATAHIPTAVYWTIRSIVACASQIIGLIGMGHEYITSTTEAWELSSMAHKVTNIHSHLMKQLTVCYQHIDEKRHIEAYQALIRLFEMVHIDNLKILRALIYAKDDQLPLVDGATRKRASLDVLRRKNVLLLISDLELSHEELSMLEQMYHEARQHPTRLESQYEVVWMPVVDRSTLWNEEKQKGFERLQSMMPWYSVYHPSLLDLAVIRYIKEAWHFNKKPLLVVLDPQGKVVNPNALHMMWIWGSLAFPFTSMREEALWKDESWRLELLADAIDPMILSWISQGKFICLYGGEDMEWIRKFTVTAQAVAQAAGTSLELLYVGKSSPREKVRKNNVMIQSENLSHVLPDLTLIWFFWVRLESMWHSKVQHNRTVENDVIMQEIVTMLSFDGSEQGWAVVSRASAEMAKGKGETILKSLMEFEEWRGIAEEKGFVPALNDYLLGLHSPLHCNRLILPGSTGSIPDRVVCAECGRAMEKFIMYRCCTD

**23170964_peptide|Lusitatissimum|Lus10014580.g|Lus10014580**

MAFRGLQPPATTSSALVPAAGRSSQLMSRGPGDQQQHGGHGNMLMMSDDNVMMKQIAATHTPDGREVDVHPLLVLVEDILNRATMQVDTTLRPTKAVTEIEDKANQINQVTMLDALSYVIDRVSKEIAYKALGGSDAHSTTVTIFETLSSYPWDAKLVLTLAAFALNYGEFWLLAQIYSSNPLAKAMATLRQLPTIMEHSGPLKPRFDALNKLIRAMVEVTRCVVQFKELPHGYISQDVPALSTALAHVPTAVYWTIRGVVACANQITLITTMGHEYALSTTEAWELSSLEHKLNNIYEHLRSQLGSCYQHIDERKNMEAFKMLVDLFETTHIDNMKILKALIHAKGDPAPIVDGSTKRKVSLEVLRRKNVLLLITDLNISHDELSILDQIYTESRHHTGRMESQYEVIWIPVVDPSIQWTDAMQTKFESLQQAMPWYTVDRPSLVEKAVIRFIREKWHFRNKAILVVLDPQGRVVSPNAIHMMWIWGSNAFPFTSLREQSLWGEETWRLELLVDGIDPMILNWIKDGKYIFLYGGDDVEWARKFTNTARAVAVASRIPLEMAYVGKSTKKELVRRVIATITVEKLSYFWQDPTMIWFFWTRLESMLFSKIQLGTIDDHDPMMLEIKKLLSYDKEGGWAVLSKGSNIISNGHGNTVLTTLVEYDLWKEHIPAKGFDLAYDDHHKKVHGVTYPCCRFEFPNTAGRIPETMKCPECHRRMEKYSTFLCCHDHEGH

**23176349_peptide|Lusitatissimum|Lus10017777.g|Lus10017777**

MEGAQNQREKLMIKDMRMSRGSDDRDMMKQILASHSPDGRHFNVKPIIHVVEAILRRANSSIYHVLNGTQSEAVENLEDTSSPAGPDDNIEYLAYSIHTVACEMSCKCSGGGDAHSTTLAVLESLKSYAWDAKVVISLAAFSVNYGEFHLVIQLLSVNPLAKSVAILKQLPDIIEQANSLKPQLDALNKLITAVVEMTKSMVDLKDLPSHYIGADTPAMSLALAHIPMAVYWAIRSIVVSATLIARNEYSDSTSQAWELSSLTHKADNIHGLLKQQLAICYLHIDEKKHEEAYQILVRLFKTHQLDNMKILKSLIYPNDDQQPLFHGATKTKVHVEVLKRKHVLLLISDMRISNEEVMVLAHMYTEAQDTSEFHYEIVWLPVVGGENWQEADERKFMELQSIMPWYSIIHPSLMQPAAVRYVKEMWHFSKRTILVPLDPAGKVMSTNAFNMLWIWGNLAFPFTMEREETLWRAEDWTLQLLVDRIDMSIMQWATQGKLICLYGGDDIEWIRKFAAATKEAASAYGTELEMVHVGKSNIPKERMTKINAAITSEGMSRCWTDPTSVWLFWTRLESMFYSKIQGGRTMENDRVLQEVMTLLSFDGGDGGWAVFCSGEAEMARAKGELALASMEDYEKWANVATEIGFVPAIRNYLQGLHTPLHCNRLILPGNEGGVPDSVVCAECGRSMEKFFMYRCCVD

**23565215_peptide|Egrandis|Eucgr.B00151|Eucgr.B00151.1**

MDQALFVPAKSKAEEEVTQGSYICLFGGEDLEWIRKLIATAKEVANIAGIMLGMVYVGKSNSKQGVQRTAATITSEELSYCWNDPMFFWLFWARLESMFHSEVHLGMSIENDPLLHVIQTILNFDRSNKRWAVFCQSVGPDMVAAEGNIVLESIEEFDKWEDDANQNGFLKALFDRLIQKHLIRLFDEIHLDNLTILKHLIYAKDDIQPLVDVLRKKTVLLLISDLNISFEDMVLLDQIYRESRARPENQFEIVWLPIVDIDPKSAAWDMTHQQIFETLQSIMPWYTVHHPSILEPAVTKYIREEWHFSKSIIIVALDPQGRLASPNALHMIRIWGNLAFPFAKEREEALWRKERWTLKLIIGGLDDRTIKEWVTQGSSICLFGSEDLEWIRKFTATAKQAAKTDGITLEMVYVGKSDSKERVQSIAAMITSEKLSYCWNDPTFFWLFWARLESILHSKAHYGITGNDPVIDEIRKIRTYDRTAQGWAILCQGAGMEMGTARSDIALLDSNGVHPSNRLITPGSGVNSPDRIVCAECGEEMGTIYRCCNE

**23565237_peptide|Egrandis|Eucgr.B00175|Eucgr.B00175.1**

MAGVPAPSRLPRAQPRGGHRMFAASDDSAMMKQIQATHTPDGREVDVKPILGLIESLFRHATPGIDGALSGKHGAEEASSEEKAYLASFDSVLEGLPYIIQKISCELSCKCSGGGDAHGTTMAIFNMISAYSWDAKVVLALAAFGVNYGEFSLMIQLYSRNPLAKSVALLKQLPDMMVHSNALKTQFDALHNLIVAMVDVTKCIIQFSELPPQYISSETPPLSTALTHIPTAVYWTIRSIVSCASQIASLIGLGNEYMPSTTEAWELSSLAYKIGSIYDHLKKQLAICYQHIDEKMHFEAFQTLIRLFDRSHMDNMTILKHLICLKDDIHPLVDVSKKSRVSLEVLRKKTVLLLISDLDIATEDIDYLHLIYKEPSAKPASQFEIVWLPIVDIDPRSAAWDMNHQQKFEALQSIMPWYTVHHPSILEPAVIRYIREVWRFSKRVIIVALDPQGKLASPNALHMIRIWGSLAFPFTKEREEALWKEESWKLSLIIDGLDDGTISKWTEQGSYICLYGGEDLGWIRKFTNAAKNVAKIANIPLGMIYLGKSNAKERVKRIAAAITSENLSHCWKDPRYFWFFWARLESMLHSKLQHGMSTENDPVMHEIMTILSYDGSDQGWAIFCHAAEPTMARAKAKNVLDSMEEINDWKDGIDEKGFIPALQDHLRQKEIDSPHHCSRLILPGIDGGIPERVVCAECGRVMEKFIMYRCCTE

**23568528_peptide|Egrandis|Eucgr.B03241|Eucgr.B03241.1**

MALVSKAPQRRSERLKFSSSDDSAMLKQIQSTHSPDGREVDVTYLLHIIEVIFERAKPFSTHETHQAAQPPLDILEDRDLREMLELLAHTINKISCEMACKCMGGGDAHATTVNIFNILTNYSWDAKVVLALAAFSVNYGEFWMLAQLYPTNYLAKAVAILKQLPETLERVDALKPKFDALTSLVTAMVDLTKCIIEFRQLPSQYISPDATELLTATAHIPTAAYWIIRSIVACASQITNLIGMSHEYIASTAEAWELSSLAHKINSIYTHLKKQLDLCYQLINEKKYLEAFEKIVHLFATPHVDNMKVLQALIYARDDQLPLLDGSTKKRASLDVLRRKNVLLFISDIDLPLDELLVLEQMYQEAKQAPTRMESHYEIVWIPVMDRSTPWDEEKQKKFETLQGMMTWYSVFHPSLIDAAVIKYIKEVWKFNKKAMLVVLDPQGRVTNPNAIHMMWIWGSAAYPFTSEREEALWKSEKWRIELLAGPVEPLIFTWISEGKYICLYGGEDIEWIRKFTNTARAVAKAAQIPLEMLYVGKSNTREKLRNNSNTILAENLSHVLPDLTLIWFFWVRLESMWHSKVQQGKSVDNDPIMQEIMMMLSFDGSDHGWAVICRGADVAKAKGDVILKTLLDYDSWKDRIGQMGFLPALGSHIHDEKTPHHCNRLILPSTTGTIPERVVCAECGRVMEKFIMYRCCTD

**23587126_peptide|Egrandis|Eucgr.G01580|Eucgr.G01580.1**

MVSLFHQLSSFSWVNKALIALSSLAVFYGDFWRLARVEPSDKLAESMAILKGLPAITKPSDLQKISVFGVLNEMIKTTLNMTQCIVDFEHESKDFPQLSTMIDVASSVYQIIISIRACSIQFTSLISIVDDNKGKDLYAFARKEIKEYQRLLQIIDAPTGNVELIKALLYTKDDPQPLFIGSKKARVHDKVESLRRKNVLLLISDLKLTSHDLSILIKIYEECKFHEEGYEIVWVPIIEQECEDVIKRFQSLQLQMPWCSVHSLKLINRVAIRIIKEKWHFKQDAIVTVLDPRGRVSNQNAMSMIRIWGRDAFPFTGAVAADIWKRPGINWFELLATDFIFPGIQEAIKAEKYIFLYGAEEPKTIQVIEDSLKKIDDKAIVALNVTKSQFFWNRLKSCMLSKLQIGGDLHEPIMQDILRLYTCFKKDGGFTVLTKGSRVVINDSHMYVTMVLVQYETWKKHVNVNGKTFDMVFKEQFDLLYVPPRCHHFYIPNMVGYIPEDVKCPIFPGMMNNIVKFECCHGAH

**23589545_peptide|Egrandis|Eucgr.H00199|Eucgr.H00199.1**

MSTILAHLLGMDHKGKPHQVDQSMWTNLLASFDDEKFMKEMVNTHAPEEEDMDVQSLFHLVDNALHGTTAIVDSIVNPTGTQGSGKPSFKPLKEGFNPPLDAINEVGCQLTCKALDTKNVRQTLVSLFHELSSYSWVNKALITLSSLAVFYGDFWRLARVEPLDKLAESMAILKGLPAITKPTDPQKIQVFGVLNEMIKTTLNMTQCLVDFEHDSKDVPELSTMIDVASSVYQIIISILACSIQFTSLISTVDDNKGKDLPTFARKVNMIHHSIKRHYEDFLQKKEEIKEYQRLQRLFNAPTDNVELIKALFYIKDDPQPLFLGSKKTKDKVESLRRKNVLLLISDLKLTSHDLSILAKIYNERKFHEERYEIVWVPIIEQDGEEVIKQFQNLQLQMPWYSVHSPKLTNRVAIRIIKEKWHFRQDTMVTVLDPLGRVSNQNALSMIRVWGWDAFPFTGAVATDIWKRPGISWFELLATDFIFPKIQEAIKAEKYIFLYGAEEPKVIQEIEESLKKMIDDGFSIVAFNVTKSQLFWTRLESCMLSKLQTRADLHEPLMQDILRLYTNFKKDGGFAVLTKGSRVVINESHVHITRVLMQYETWKKHVNVNGKTFDMIFREQFDRVYILPRCHHFYIPNMVGYIPEDVKCPVCPRMMNNIVKFECCHGAH

**23589546_peptide|Egrandis|Eucgr.H00200|Eucgr.H00200.1**

MTTVLAHLLGMDRKGKTHQIDQSMWTNLLASFDDEKFMKEMVSSHAPEEGDVDVQSLFHLVDNTLHGTTAIVDSIVNPKGIQGSGSHEFKPLKEGFNPPLAAINEVGCQLTCKALDTKNVRQTLVSLFHELSSYSWVNKALITLSSLAVYYGDFWRLAQVEPSDKLAESMAILKGLPAITKPSDPQKIHIFSVLNEMIKTTLNMTQCIVDFEHDSKDVPELSTMIDIPSSVYQIIISILACSIQFTSLISMVDDNKGKDLPTFARKVKMIHHTIKRHYEDFLQKKEEIKEYQRLQRLFNAPTDNVELIKALFYIKDDPQPLFLGSKKTKDKVESLRRKNVLLLISDLKLTSHDLSILIKIYNERKFHEGRYEIVWVPIIEKEGEEVIKQFQNLQLQMPWYSAHSPTLINGVAIRIIKEKWHFRQDTMVTVLDPQGRVSNQNAMSMIRVWGWDAFPFTGAVATDIWKRPGISWFELLATDFIFPKIQEAIKAEKYIFLYGAEEPNVIQEIEASLKKMIDDGFSIVAFNVTKSQLFWTRLESCMLSKLQTRADLHEPLMQDILRLYTNFKKDGGFAILTKGSRVVINDSHLHVTKVLVQYETWKKHVNVNGKTFDIVFREQFDRVYVLPRCHHFYIPNMVGYIPEDVKCPVCPRMMNNIVKFECCHGAH

**23589547_peptide|Egrandis|Eucgr.H00201|Eucgr.H00201.1**

MASKTLGLILRRERSKVDQSMLNSLLAIFDDEKFMKEMVDTHAPDEGDVDVQSLFLLVDNALRGTATIADSVINPKGTQGSGGPAFKPPKEGFNPPLAAINEVACEITCKALDTKNVRETMISIFHELSSYSWVNKAVITLSSLALFYGDFWRLAQIQPSDKLAESLAILKGLPAITKPSDVQKIQVYGVLNEMIKTTLSMTECIVEFEHDSKDVPELSTAIDIPSSVYQIIISVLACSIQFTSLISLIDDYKGKDLPTFVRKVNMTHHTIKRHYEDYKQKKEEIREYQRLQRLFNAPTDNVELIKALVYIKDDPQPLYIGSKKTTDKVESLRRKNVMLLISDLKLTSHDLSILIKIYSERKFHEERYEIVWIPIIEQEGDDVNNQFKNLQSQMPWYSVRYPTLINKVAIKIIKEKWHYRQETIVTVLGPQGRVSNKNAMSMIRLWGWDAFPFTESVGASLWSKPGISWFELLLTDMVIPKINEAIKSRKYILLYGAEDNKAIHEIEEPMKKIIEDGVPIAAYNVTKNQLFWSRLESLMLSKLQTKADVHEPLMQDILCLYTNFKKDGGFAVLTRGSQVVINDSIIDVTKVLSQYETTWKKQVKGNEKTFDAAFKEHRERVFVLPRCHHFYIPNMVGYIPEDVKCPVCPRMMKNIVKFECCHGAH

**23589550_peptide|Egrandis|Eucgr.H00204|Eucgr.H00204.1**

MAGRLISAVFSGSQRAKQPDQGLLNSLVAVFDDNKFMKEMVDTHAPDESDVDVQALFALVENTLKGSTAIVDNVVNLNKASSTPAAIPEFKPAKEGFTPPLSTISEVTCQITCKALDTKNVRETMSSIFHELSSYSLVTKAVVTLSSLGLYYGDFWRLAQIEPSDKLAESMAVLKGLPAITKRVETQKIQVFGVLNDLIKTTLEMTRCIVEFDHDSKDVPELAKVIDIPSSVYQIIIAVLGCSIRFTDLITTTPVDESKGRDLPAFARKVNIIHHNIKRQLEDFKQKKEEIREYQRLQRLFTAPTDNVELIKALFYTKDDPQPLYIGSKKNTDKIEYLRRKNVLLLISDLKLSSHDLSILVKIYNEPKFHENHEILWIPVVDQDSEDLPNVFKNLQSQMPWCSTHYPTLINKVAIRIIKEKWHFRNEPIVVVLDPQGKVESQNAMSMIRMWGFNAFPFTDGVVATLWRRRDISWFELLVNDSIAPNVQEARHPNNPKEQLNCSKLNELHYDFVISINQIKSEKLIFLYGGEDTKEVHEVEVHLKKISDDGNSIVAYNVGKIQLFWTRLESCMFSMLQGKADVLDPLLQDILKLYTNFKKDGGFALLTKGTRVVINTPLASSSKVLAQYDTWKKQVNVDGKTFDVALKEHHDKVVAPQTCHHFYIPNMVGYAPENVKCPVCPRVMKNIVKFECCHGAH

**23589551_peptide|Egrandis|Eucgr.H00205|Eucgr.H00205.1**

MASKTLGQILRRERSKVDQSMLNSLLAIFDDEKFMKEMVDIHAPDEGDVDVQSLFLLVDNALRGTAAIADSVVNPKGTQGSGSPAFKQPKEGFNPPLAAINEVACEITCKALDTKNVRETMISIFNELSSYSWVNKAVITLSSLALFYGDFWRLAQIQPSDKLAESLAILKGLPAITKPSDVQKIQVYGVLNEMIKTTLSMTECIVEFEHDSKDVPELSTAIDIPSSVYQIIISVLACSIQFTSLISLVDDYKGKDLSTFARKVNMIHHTINRHYEDYKQKKEEIREYQRLQRLFNAPTDNVELIKALVYIKDDPQPLYIGSKKTTDKVESLRRKNVMLLISDLKLTSHDLSILIKIYSERKFHEERYEIMWVPIIEQEGEDVNNQFKNLQSQMPWYSVRYPKLINRVAIKIIKEKWHYRQETIMTVLDPQGRVSNKNAMSMMRLWEWDAFPFTESVGASLCSKPGISWFELLLTDMVIPKINEAIKSQKYILLYGAEDNKAIQEIEEPVKKIIEDGVPIAAHNVTKNQLFWSRLESLMLLKLQTKADVHEPLMQDILSLYTNFKKDGGFAVLTRGSRVVINDPIVDVTKVLSQYETNWSKQVKGNETTFDAAFQKHRERVFVLPRCHHFYIPNMVGYIPEDVKCPVCPRMMKNIVKFECCHGAH

**23589552_peptide|Egrandis|Eucgr.H00206|Eucgr.H00206.1**

MAGRLISAVFGGSQRAKQPDQGLLNSLVAVFDDNNFMKEMVNTHAPDEGDVDVHALFALVENTLKGSTAIVDNVVNPNKASSTPAAIPEFKPAKEGFTPPLSTISEVTCQITCKALDTKNVRETMSSIFHELSSYSLVAKAVVTLSSLGLYYGDFWRLAQIEPSDKLAESMAVLKGLPAITKPVEPQKIQVFGVLNDLIKTTLEMTQCIVEFDHDSKDVPELAKVIDIPSSVYQIIITVLGCSIQFTALITTTPVDESKGRDLPAFARKVNMIHHSIKRQLEDFREKKEEIREYQRLQRLFTAPTDNVELIKALFHTKDDPQPLYIGSKKTTDKIEYLRRKNVLLLISDLKLSSHDLSILVKIYNEPKFHENHEILWIPVVDQDSEDLPNVFENLQSQMPWCSTHYPTLINKVAIRSIKEKWHFRNEPIVVVLDPQGKVESQNAMSMIRMWGFNAFPFTDGVVVTLWRRRDISWFELLVNDSIAPNVQEAIKSEKLIFLYGGEDTKEVHEVEVHLKKISDDGNSIVAYNVGKIQLFWTRLESCMFSMLQGKADILDPLLQDILKLYTNFKKDGGFALLTKGTRVVINTPLASSSKVLAQYDTWKKQVNVDGKTFDVALKEHHDKVVAPQTCHHFYIPNMVGYVPENVKCPVCPRVMKNIVKFECCHGAH

**23592479_peptide|Egrandis|Eucgr.H03162|Eucgr.H03162.1**

MDSLLNLLSSFSWDAKAVLMLLTLSVYFVENRRHAQIQASDKVLCLVAILRGLPSEKQQDEVFVELIKQTLAYTKCVFEYFERYSAHEDSLKRSALIDTSANFFHIIVIVLGCSILYNGWTSESNEFAWQDLLDLLKQVTKEYEVFKKRVQDFEKEEEEQLYWQIEQVSESCSDIVELLRTLFDAEVYQCRQSTTVKVEKLKDKRVMLLISDSNLSNDDLATLTSIYNVSAFKSNNYEIMWVPIMEARDEVMEKQFLDMRSRMQWYSCNSMVSRKAAKFIRDKWQFSQQTKVVVLNEQGKVVNMDAISMISLWGWDAYPFTEAKGKELWKSPEINWFKLVVNKTIYPFIEQCFERRELLFLYDSAEDSNAVREIEEHLEKINYSVSHIAIRINTKRKQFLTRLEYCISSKMQANSDVYDSLTQDLFELYTSYKKHGGFAIMARGSSTIVNARLTDFTTVLSQHQMWIPRVTQTQTFEAVFQEHYRHAIVKPHCHRFYIPNMVGNMPKSLRCKKCSQNMKISVTFECCHGEH

**23592480_peptide|Egrandis|Eucgr.H03163|Eucgr.H03163.1**

MLSSFSWDAKAVLTLLALSVYYAEHWRLTQIEESDDLLSLTAMLRGSHAIGKSSDTQRIKAFVALNNLIKLTLEFTKCAVEFVSYPRDFGEISASIDISARFYYIIVIILGCSVQFSGLISMSNEYVITLTLLLTTFVNRLGFNSKLSLSLSLSLSLSLSLSDCFTSMLNLFLDYFRFLGQNLWTFSDQVTKKYESFKKEVEYLREKHAEQISYEKIKKLSKHCTDIVELMAALFCTKHDFLTVYHCSKRTTVTVEKFRNKIVMLLISDLDLSDEDLKTITSIYSESNIQRSNYKIMWVPIVEEHNEVTQKKFLEKRSKMRWYTSNSMVSKPAAKFIRKKWQFRQQTKVVVLNRQGKVENMDAMAMIRLWGQEAFPFTESKGKELWNKQGINWFKLVVNMVFPDVEKSIEGEELIFLYGSDEDPQTVQKIEKYLSEINGYIGSYKVINISTKREQFLTRLESCLSLKMQASSDVYDSLAQELLQLYTSYKQFGGFAVIARGSRVVVNTRLMDFETVLSQHQMWVKAAQPFEAAFKRHYDEVIKKPRRHHFYIPSVAGNMPENIKCAKCSRYMKTAVSFECCH

**23593328_peptide|Egrandis|Eucgr.H04052|Eucgr.H04052.1**

METLALSNSSRGGSSGHRTNISGGFGIIHHIDIHQFIEKLSYDSTNEAKCNANELLKIISNILTSAIELVRGDSNTQKVPMHASETIISSDISHLINWICCQMTCEATRGENLGEILPMIFQKLSSYQWTTQVLLVLAAFSLHYGEFWYICRAPSDGGNALSTCLLKGLISLKKKLWSEKNMKTAITTLNNVVHDLLELVTSLVGLYKLHEQHRVKHVPGLESFGEIPTWSCETIIAILVTGNYFARLVNADDKFAESELENLVASVSHLKSTVLQKVEGCKRRIAQMEEYQKYAKKVEAPVDIVDFLKSLLAAKASSRDPIVTGPNNTPVKLESFRKKKGLLIISGLEISADDAATLKRIHDHLKHSQTEKETHYEFVWIPILDAKPPNNSEALFKSLMPWAFKVDPRKMNVLAPKYIETEWGFRRETMVVVLDSHGWVENLNFMHMVRIWGTFVFQETWKDPFNWVQLVITPTVPREKLVVIKTAYTLFYGGTAAMKSDIAPEIPHLSMVHIKDTKGFFIRLRSCLISRLEAVKINPGKINPLIDPIVCEMCEAYKACHIGGFAILTKGPDLQKQHISLFDAASTLANDLKRVKELVDKDKGIDLAEAFKKKEPGVQELSGRKHFYVPHYHGLGDLCCPDDSWKVSYDLSFTCCHSGSDQGGARQAKSSTDANAK

**23596372_peptide|Egrandis|Eucgr.I01709|Eucgr.I01709.1**

MAMTTTGQMMPPSMQRLIKGGDRSMITMSDEDVLTKQIKETHAPDGREVDVKPLLHLVKDILNRSTLNVDAVAAEGSPAHTENMEDQTYRASDTDLLDALSYIIERVSSELHAKALTGGEAHSTTLAICNMLAPFSWDAKLVLTLAAFAFNYGEFWLLAQVSSTNQLAKLMATLKQLPMIMEYSSVLKSRFDALNSLIQAMLKVTKCVVDLKELPSAYISSEFPALSAAMNHVPTAVYWTIRSAVACYGQITSLTSYGLEYITSTTEAWELSTLAHKLTNIYEHLQKQIGICYQHIEEKRRLDAYKSLLNLFETIHIDNMKILKALIYPKDDVLPLVEGSTRQRVNLEVLRRKNVLLLISGVDISQEELSILEQIHSESKLHAIRHEIHQYELVWIPMVDRSVEWTGPKQKQFEALQSSMPWYTVYHPSLIDDLVIRFVKEKWHFRNKPILVVLDPQGKVVSPNAIHMMWIWGSNAFPFTTLREEALWKEEKWRLELLVDGMDPTILNWIRDGKYIFLYGGDDIEWIRKFTAAARQVAQAARIPLEMIYVGKSSKREKVRQVIATINVEKLSSTWQDLTLVWYFWTRLDSMLFSKIQLGKADEDDPMMQQIKRILSFDKTGGWALLSRGSAIAVNGHGTTVLPALVEYDLWKEHVVTKGYDVAFKDHHDKIRDVTLPCCRFEFQSAGGRIPEGMRCPECQRRMEKLTTFACCHDEVVPTI

**23596377_peptide|Egrandis|Eucgr.I01714|Eucgr.I01714.1**

MAMTTMTTDKVMPLSMQRLIKGGDRSMIALSDDSVMMKQVVETHAPDGREVDVKPLLLLVEDILNRATLNVDTLADTGSTKHAENMEDKIYQANFNATLDVLSPIIERISCELSAKALSGSEAHATTLTLLNMLAPFSWDAKLVLTLAAFAFNYGEFWLLAQIQSSNQLAKSMAVLKQMTLIMEHSAALKSRFDKLNSLIQAMLEVTRCVVKLKELPSAYISSDVPALSTAMAHVTTAVYWTIRSAVACASQITNLTTFGYEFITSPTEAWDLSTLHHKLKNIYEHLQKQIDICNQYIEEKRYLEAYQNLLNLFETIHIDNMKILKALIYPKDDILPLVEGTTKKRVNLEVLRRKNVLLLISGLEILQDELAILEQIHSESKTHATRHEIHQYELVWIPMVDRSVEWTDPMQKKFETLQSTMPWYTVYDPNLIQKAAIRLIKEKWHFRNKPILVVLDPQGKVVSPNAIHMMWIWGSNAFPFTTLREEALWREEKWRLELLVDGMDPTILNWISDGKYIFLYGGDDIEWIRKFTAAARQVALAARIPLEMVYVGKSNKREKVRQVIATINVEKLSSTWQDPTLVWYFWTRLESMLFSKIQLGKADEEDHMMQQIKRLLSFDKAGGWAVLSRGSAITVNGHGTTVLPALLEYDLWKEHVATKGYDVAFKEHHDKIHDVTHPCCRFEFQSMGGRIPEGMRCPDCQRRMEKLTTFVCCHDEVVPTI

**23596378_peptide|Egrandis|Eucgr.I01715|Eucgr.I01715.1**

MAMMTTTSKMMPPAMQRLIKGGDRSMITMSDDTAMMKQVTDTHAPDGREVDVKPLLLLVEDILNRATLTASTPTMGSLYAEHMEDKGYQASFNAVLDVLSYTIERISCELSAKALTGGEAHSTTLAICSMLAPFSWDAKLVLTLAAFAFNYGEFWLLAQIYTTNQLAKSMAILKQLPMIMEHSGALKSRFDALNGLIQAMLKVTRCVVDLKDLPSAYISSEAPALSAAMAHVPTAVYWTIRSAVACASQITSLTSYGIEYISSTTEAWELSTMDHKLKNIYEHLQKQIDICKQYIDEKRDIEAYQTLLNLFETIHIDNMKVLKALIYPKDDILPLVEGTSKKRVNLEVLRRRNVLLLISGLDISQDELSILEQIHSESKAHASRHDIHQYELVWIPLVERAGELTGPMQTKFETLQSTMPWYTVYHPNLIHKAVIRFIKEKWHFRNKPILVVLDPQGKVVSPNAIHMMWIWGSNAFPFTTLREESLWRDEAWRLELLVDGIDQTILNWIKDGKYIFMYGGDDIEWIRKFTSEARKVALAANISLEMVYVGKNSKREKVRRVIETINFEKLSHTWVDLAMVWFFWTRLESMLFSKIQLGKVDESDDLMQQIKRLLSFDKVGGWAILSKGSSITVNGHGTTVLPTLLEYDLWREHVPTKGYDVAFKEHHDKIHDITHPCCRFEFQSTAGRIPESMRCPECQRKMEKLTTFVCCHDEAIPTIYD

**23605982_peptide|Egrandis|Eucgr.L00852|Eucgr.L00852.1**

MASRNLGQILRRERSKVDQSMLNSLLAIFDDEKFMKEMVDTHAPDEGDVDVQSLFLLVDNALRGTAAIADSVVNPKGTQGSGGPAFKPPKEGFNPPLAAINEVACEITCKALDTKNVRETMILIFHELSSYSWVNKAVITLSSLALFYGDFWRLALIQPSDKLAESLAIMKGLPAITKPSDVQKIQVYGVLNEMIKTTLSMTECIVEFEHDSKDVPELSTTIDIPSSVYHIIISVLTCSIQFTSLISLVDDCKEKDLLTFVRKVNMIHHTIKRHYEDYKQKKGFNLNAEEIREYQRLQRLFNAPTDNVELIKALVYIKDDPQPLYIGSKKTTDKVESLRRKNVMLLISDLKLTSHDLSILIKIYSERKFHEERYEIVWIPIIEQEGDDVNNQFKNLQSQMPLYSVRYPTLINKVAIKIIKEKWHYRQETIVTVLGPQGRVSNKNAMSMIRLWGWDAFPFTESVGASLWSKPGISWFELLLTDMVIPKINEAIKSRMYILLYGAEDNKAIQEIEEPVKKIVEDGIPIAAYNVTKNQLFWSRLESLMLSKLQTKADVHEPLMQDILCLYTNFKKDGGFAVLTRGSQVVINDSIIDVTKVLSQYETTWKKQVKGNEKTFDAAFKEHRERVFVLPRCHHFYIPNMVGYIPEDVKCPVCPRMMKNIVKFECCHGAH

**23606328_peptide|Egrandis|Eucgr.L01341|Eucgr.L01341.1**

MDQALFFSAKSKAEEEVRLGSYICLFGGEDLEWIRKLTATAKEVAKTAGIVLGMVYVGKSNSKERVRRTAATITSEKLSYCWNDPRFFWLFWARLESMLHSKVHLGNSIENDPLLPRIQTILNFGLSDEGWAVFCQGVGPDMVEAEGNIVLENLEEFDKWKDDANQKGFVNALRDHLIQKRLMRLFDEIHLDNLTMLKHLIYAEDDIQPLVDDMELLDHIYRESRARPENQFEIVWLPIVDIAPKSAAWDMTHQQIFETLQSIMPWYTVHHPSILEPAVIKYIREEWRFSKSIIIVALDPQGRLASPNALHMIRIWGTLAFPFTKEREEALWREESWTLKLIIGGLIDGTIKEWVTQGSSICLFGSEDLEWIRKFTATAKQAAKTDGITLEMVYVGKSDSKERVQRIAAMITSEKLSYCWNDPTFFWLFWARLESILHSKAHHGITRNDPVIDEIMKIRTYDKTAQGWAILCQGAGMEMGTARSDIALLDSMGVFHAPT

**PGSC0003DMP400029642|Stuberosum|PGSC0003DMG400016967|PGSC0003DMT400043688**

MASVNPLNEKESMNNPMTTNPMNNMSSGSADVVANRMHPMSNATTGHHINPLNAQINPLNAHINPLNVQTNPHSVVKPVSHDMIPASVVPAAHHTGPINPRTSNLAARPPHRRGDHHMFLTSDDNAMMKHIVETHIPDGRDFDVKPLVHIIEDIVHRATPIAGRVHEAKVQAHLEALEEKAPHSGLTEILNYLAYPIHRISMELISKCANKEDAHSTTMSLLHSLTTYAWDTKVVITFAAFAQQYGEFGLLVHQYTTNPLAKSIAIIMELPEIMTRQDVLKQKFDAIHDLIDKMLDVTKCIIEFRDVQTSHSQHVITQELEMLINTAHISTAAYWTMRAAVMCAAMILNLIAIGHEQISSTSEAWELSSLTHKLANILDHLRKVLDLCHQKIEEKRQHDAFEALLRLLRTPHIDNMKILSILIHSRDDQLPLFDGTHKRRVSLDVLRRKHVLLLISDLDIAPEELFVLHHMYDESKTQPNRPESNYDVVWIPVVDKRLSPWTEAKQMKFEEVQASMPWYSVAHPSMIDPAVIRCIKEVWGFKKKPQLVVLDPQGKEANNNAYHMLWIWGSLAFPFTKARETALWKEQTWNIELLADSIDQNVFTWISEGKCICLYGGEDIEWIRSFTSATRAVANAARVPLEMLYVGKKNPKERVRKNSSIIQIENLSHVVQDQTLIWFFWERLESMWHSRTQQDIPGETDPILQEIVTILSYDGSDLGWAVFSRGLAEMTRGKGDLIVQVMKGFDRWRDEVSDVTTFVPALDRQLRDLHSPHHCTRLILPSTTGHVPERVVCAECSRPMEKFIMYRCCTD

**PGSC0003DMP400029652|Stuberosum|PGSC0003DMG400016971|PGSC0003DMT400043699**

MLDVTKCIIEFRDVQTSHNQHVIITQELEMLINTAHISTAAYWTMRAAVMCAAMILNLIAIGHEQISSTSEAWELSSLTHKLANILDHLRKVLDLCHQKIEEKRQHDAFEALLRLLRTPHIDNMKILSILIHSRDDQLPLFDGTHKRRVGLDVLRRKHVLLLISDLDIAPEELFILHHMYAESKTQPNRPESNYEVVWIPVVDKRLSPWTEAKQMKFEEVQASMPWYSVAHPSMIDPAVIRCIKEVWGFKKKPQLVVLDPQGKEANNNAYHMLWIWGSLAFPFTKAREEALWKEQTWNIELLADSIDQNIFTWISEGKCICLYGGEDIEWIRSFTSATRAVANAARVPLEMLYVGKKNPKERVRKNSSIIQMENLSHVVQDQTLIWFFWERLESMWHSRTQQDIPGETDPILQEIVTILSYDGSDLGWAVFSRGLAEMTRGKGDLIVQVMKGFDRWRDEVSDVTTFVPALDRQLRDLHSPHHCTRLILPSTTGHIPERVVCAECSRPMEKFIMYRCCIE

**PGSC0003DMP400026590|Stuberosum|PGSC0003DMG400015156|PGSC0003DMT400039197**

MASHALVPAATTRGMKPTQQAMAKRERPVFSLSDDHAMSKKILDTHNPDGREVDVNVILHIAEEVFQHAYPAGMDGVLHGAGAHHLEGNIEALKLEENASLAFDGILEGLAYIIHKVSCELTCKCSSGGHDTHSTTMSILGMLSGYQWDAKLVISLAAFAVTYGEFWLVAQMFATHPLAKSVAILKQLPDIMEHHGSLRSRFDAINELIKAILEVTKIIIEFKKLPSQYISEDQPPLSVAITHIPTAVYWTIKSIVACASQLTTLLGMSYDMIVATTADTWEMSSSTHKLRNISEHLRAELNRCYQHIQDKMHVEYFQMLVHLFEATQFDNMKIMRAMIYIKDDLLPLEVGTTHTRASVEVLRRKTVLLLLSDLEASHEEILVLSQIYLESRSRPEFQYEIVWLPIVDRSKGWNDAQEQKFKELQALMPWYTLHHPSLLEPAIVKFVKEKWHFSKKMMLVTLDPQQGKVACPNAIHMAWIWGNLAYPFTISKEESLWNMESWRLELVVDGIDPNLIDWMASGKFICLYGGEDMDWIRNFTKSARSVAQRAGIDLQMLYVGKSNNKERVRRINSMITAENLSYCLMDLTSVWYFWTRIESMFYSKMQLGKTIQEDKVMQEVLTMLSFDGSDQGWALISRGSFEMARAKSQIITKTLEDYTIWEEDARSKGFVPALIEYFLQLHTPQHCNRLILPGLDGDIPEMIVCAECGRAMERFFMYRCCTD

**26290867_peptide|Gmax_v1.1|Glyma02g00280|Glyma02g00280.1**

MALVTSLSPATPSSVQTNTLNPLGWTDDQILEKVYITHVHTAERYDVESLFNVTTNIIKRATALADSVAVKTGTPVGLIEDKVPLSTFDPPFLKLKHIASQMMNTPHGEHHAHNTAMSILDQLRTYTWDGKAIMGLAALALEYGNFWHLVQTPTGDHLGRSLAQMSRVHIVERNRQAVADYNSLVKNLLIAVECITELERLSTKGYDLKDVPALAEAMQETPVAVYWAIVTTVVCANHFDFLLGESDSRYEIANFDDKLAAVISKLKANLTRSRKKIGDLEDYWRRKKLLQTPTEIVEVLKVLIYHNEVHDPHVYDGITRQMVSIEVFRKKHVLLFISGLDSIRDEVRLLQSIYEGLQEDPREVKGYRKEDFRILWVPVVDEWNLLHRAEYDNLKLEMPWYVAEYFYPLAGIRLIREDLNYKNKPIIPVLNPQGRVVNYNAMHMIFVWGIDAFPFRPSDDDVLTQKWNWFWAEMKKVNPKLQDLIKADSFIFIYGGSDKKWLQDFTQTVEKIKRHEIIKRADAVIEHYPFGREDHRIVPRFWIGIESLFANMIQKTQKDPTIEEIKSLLCLKQQQPGWVLLSKGSNVKLLGGGDPMLATAADFEIWKEKVLEKAGFDVAFKEYYEQKRRNYPQECSHMQLANYPADILHPINCPDAACGRSMEIASVSYKCCHGQTAHHKAEVPESGDVMIEKKLYAS

**26298425_peptide|Gmax_v1.1|Glyma06g18430|Glyma06g18430.2**

MSNHTRALPPAATPKMQQRKERQRFSTSDDSAMTKQVQATHAPDGREIDVKPILQIVDEILVRFIARTVEGYEVKRDQDALEMTAALAEFDMLDSLAYIINKISCELSCKCSGGGDAHSSTMVLLNYISSYAWHAKVVLTLAAFAVIFGEFWLVAQLSAENTLAKSVALLKQLPDIAENFMSMKPHFEALIRLVKAAMDVTMCIVEFKELPSEYISEDTPPMSVASTHIPIASYWVIRSIVACSSQISSFIGMRNESISSTTEAWELSSLAHKVSSIYEHLKNQLVLCYQYIDDKRHIEAFHNLIRLFETVHVDNMKILRALIYAKDDVLPLVDGTAKSRVSLEVLRRKHVLLLISDLDLSQEEILVLDNLYKDARARGDTQYEMVWIPIVDKATWNDMSKQKFEYLQSLMAWYSVYDPFIIEPSAVKYIKEVWNFSKKAILVALDPQGRLSSPNAVHMIWIWGNLAFPFTSEKEESLWKQEIWSLELLVDGIDPTVLEWMTEGKLICLYGGEDLEWIEKFTATAISVAKAGKFELEMAYAGKSNAKERMQKMIKTFTTRKFSYFWPNVTSIWFFWTRLESMLYSKLLHGRTVEKDEIMSEVMTVLSFDGSDRGWAIFCRGATEMARAKGDSALRCLQDFDKWKGRIEEDGVVHAINDYLNKNKPPHHCNRLILPGSTGGIPQKVVCAECGRQMEKYFMYRCCVE

**26302685_peptide|Gmax_v1.1|Glyma04g36440|Glyma04g36440.2**

MSNMSNPKMQQRRERRMFSTSDDSAMTKQVEATHAPDGREIDVKPILQIVDEILVRFIARTVEGHEVKRDQDALEMTAALAEFDMLDSLAYIINKISCELSCKCSGGGDAHSSTMVLLNYMSSYAWHAKVVLTLAAFAVIFGEFWLVAQLSAENTLAKSVALLKQLPDIAENFMSLKPHFEALIRLVKAAMDVTMCIVEFKELPSEYISEDTPPMSVASTHIPIASYWVIRSIVACSSQIASLVGMRNESISSTTEAWELSSLAHKVSSIYEHLKNQLVLCYQYIDDKRHIEAFHNLIRLFETVHVDNMKILRALIYAKDDVLPLVDGTTKSRVSLEVLRRKHVLLLISDLDLSQEEILVLDNLYKDARARGDTHYEMVWIPVVDKATWNETSKQKFEYLQSLMAWYSVYDPFIIEPSAIKYIKEVWNFSKTAILVALDPQGKLSSPNVVHMLWIWGNLAFPFTSEKEESLWKQEIWSLELLVDGIDPTVLEWMTDGKLICLYGGEDLEWIEKFTTTAISVAKAGKFELEMAYVGKSNAKERMQKMIKTFTTRKFSYFWPNVTSIWFFWTRLESMLYSKLQHGRTVENDDIMSQVMTVLSFDGSDRGWAIFCRGATEMARAKGDSALICLQDFDKWKDRIEEDGVVQAMNDYLNKNKPPHHCNRLILPGSTGGIPQKVVCAECGRQMEKYFMYRCCVE

**26306078_peptide|Gmax_v1.1|Glyma08g13870|Glyma08g13870.2**

MAMVPRKMQSRPSRHIFSASDDTTMTKNIRATHAPVDGHIGVRPLLHVVQDIFHRAASLIPGIVQGKQVQMDAMKDSAYQSDLANVIDISYHTINKISCEICCKCSSGGDVHATTMGILGMLSSYSWDAKVVIALAAFAANFGEFWLVAQLYATNRLAKSVAKLKHIHETLEQVDDLGPKFQTVNNLLKAMLDVTNNIVQFHELPSQYIDPEAPEMLTASNLIPGAVYWTIRSIVSCASHILGITGLGQGYMTSTTETWELSSLAHKLDNINSHLRKQLTVCRQHLDDNRQREAFATLRLLFETPHQDNSKVLKAMFCSKDDPLPLFDGSSKQRVSIEVLRRKIVLLYITDVHNVPDQELVIFEQMYQESRQDSTRLESQYELVWIPVVDKAIPWNDVKPKFEKLQSMMSCYSLYDPSLLEPATIRYIKEVWLFKTKPILVVLDPQGKVVNLNAIPMMWIWGSLAYPFSSSREEALWNAETWGLVLLADSIDPSLLEWISEGKYICLYGGDDIEWIRKFTNTAYSLARTLQLPLEMIYVGKSNPGKKVQEINNAVQTEKLSNVLPDLAISWFFWVRLESMWHSKSQQSKTVENDHIMHEVMRILTYDSGDPGWAVISQGTGKMAQGKGDTFLKCLNEHEQWKDAAKDKGLLPAMDDYIKELQTPHHCNRLILPGTSGGIPDKVSCAECGQTMEKFYMYRCCNE

**26314812_peptide|Gmax_v1.1|Glyma13g03611|Glyma13g03611.1**

MSKSLSSTNVQQGKTLLQNPFEFSDEQILDNVYRTHFHCVEKCDVASLHTVASCVISHSIEITDTVITKGSQLSDRFREDTTITSQQLTAKLKRIACQMVCTARGDHYAHHTTMLILEQLKAYSWDAKALIVQAAFALEYGKFLYLPLTTQYQMSEKSLADLNGLLMIQHNTQHLTFFNSVVKKVMQVIECITEWKRLTSAGYDIKDVPTLAETLHEIPVVVYWAIFTFVTCTGQIDDFTTDHKIHKHELSKNFENKLDLILRNFKEHLEMCGREIGRIEDYTRRKNIVIHTGKDIVKVLKALIISGENRESRQSVFNVLTGEQIKIEEFKKKHVLLFISGLDSIEEETLLLKSIYEKLKEKPREVEGYRKEDFKILWIPIVDEWNEERRKTLETKLQRTKFGWYVVKHFSFETGFKLIKEVFNYKERSIIPLISPEGRVENIDTKQIISVWGIDGFPFRTSDHTRLTQQWNWFWSEMTKLNPRIGDLIEEDRYLFIYGGTDAMWIQEFTTAVEKLKRVVDSISLQIDITIESYQLGREDPKVVPRFWIAIDSLLANRKQQMMKGGDQGVQDFATREIKRLLFLKQDPKGWVILSKGYNVKLLGQGEAMCRSVRDFGIWHGKLHEEVSFDVAFKEYYESIKVKDCPKKCEHSEISNYPTDILAHIPCPNMDCGRSMEVTSVNYRCCHGLEP

**26316356_peptide|Gmax_v1.1|Glyma13g26030|Glyma13g26030.1**

MANKLEQVRRRTGSINSFQRLPAMKRIACQMMCTARGEQYAHQTTMLILEQLRDYSWDAKAVIVLAAFALEFGKFWQLAHIPRDKLGQSLAELNGLQSIMENIQHLANFNNLVKKIVQVVKCITDWKKMITAEYNVKDVPSLTDTLHEIPVLAYWTISTLVTCTSHIDFLGDKGYRYDLSKFDYKLDFILKNFKDHQDKCSTQIGQIEDYSRRKDIITSIQTDTQIDIVKFLEALIIPSYSQDSRPIVYNGLTGPQVALGEFKNKHVLLFISGLDHIDNEIQLLKSINAKLKEEPNELEGYRKEDFKILWIPIVSVWDEEQKKKLDVTKVEWYVVKEFNFQTGIDLIKEVFNYKGNPIIMLISPEGKVENSDAKQIISKWGIDGFPFRTSDHTRLTQQWNWFWNEMITLSPIIRELIKRDSYIFIYGGTNTKWIQDFTTAVEKLKKNETLTLEEETTIESYPLGRDSPKIVPRFWITIDNLLASRKLTKKGSEQVQDSTTREIQKLMFLKQDPLGWAILTKGSHVKLLGHGDAMLRTVTDFESWKGTMHNEVSFDVAFKNYYDKCKVKSVPPKCEHREFANYPTDILAHIPCPNKCGHEMEVSSVKYMCCHGREASDIA

**26316440_peptide|Gmax_v1.1|Glyma13g03620|Glyma13g03620.2**

MICTARGEQYAHQTTMLILEQLRDYSWDAKAVIVLGAFALEYGKFWQLAHIPRDIKLGKSLAELNGLQSIMGNVQHLANFNSLVQKIEQVVKCITDWKKMITVEYNVKDVPSLTDTLHLIPVLAYWTISTSWSLVPATLISRLQLLGFRGYRYDLSKFDYRLDFILKNFKDHQDKCSTQIGRIEDYSRRKDIITSIQTDTQIYIVKFLEALVRKITYPLLIIISLTLLQAALGEFKNKHVLLFISGLDHIDNEIQLLKSIHVKLKEEPKELESYRKEDFKILWIPIVGVWDEEQKKKLDVTKVEWYVVKEFNYQTGNPIIMLISPEGKVENSDAKQIISKWDIDGFPFRTSDQTRLTQQWNWFWNEMITLSPIIRELIKRDSYIFIYGGTNTKWIQDFTTAVEKLEKNETLTQEEETTIESYSLGRDNPKIVPRFRIAIDNLLASRKLTKRGGEQVQDSTTREIQKLMFLKQDPLGWAILTKGSHVKLLGHGDAMLKTVSDFYAWKGTLNNEVGFDVAFKDYYEKFKFKSVPHKCEHREFANYPADILAHIPCPNKCGHEMEVSSVKYMCCLGRETNPSSKHREPSPPSPWLPSTPNR

**26337108_peptide|Gmax_v1.1|Glyma20g34670|Glyma20g34670.1**

MSIIMSTKSSLKSLLQKGENEHNPLTMSDEQILEQIYSTHVHSDTKFDVDSLFTLVENTLRRSTHIVDNLVQGSHASLEHIDDKIPQFNSPLCTLKQISFEMSCKPPSEEIGHRTTLAILNKLSNYEWDAKAVLTLAAFALEYSEFWLLAQYQPTDPLAKSVAILKRVPVLAKPAALQKHRQAILEVNNLVKATLQVIEVIFELEKLTTYDTKDVPALGLAIEQIPVDVYWAIITIVAVVTQIDCLTTDSEHKQELSHYGQKINIILSKLRKQITLCRQQIDEAQYYRKLRKFFQTPTEIMEVFKVLIFNKDAPQPLFDGATKTKVDITVLKKKNVYLFISSLDITEEEISVLRPVYDSIKTNDQYKIVWIPIVEEWTEQLHKKFEVLKSKMPWYVVQHSGTIAGYKYIKEEWHFKKKPMVVVLSPQGKVQHSNAFHLIQAHGTRAFPFTTLNEEKINSENDWVGSVLGSIHPSISTSIKEQKYIFFYGGNDKDWIQQFTKYVTALANDAAIKEAKISIELFCVDKEDKSLVRRFWSGIESLFVTKVHKQADAVTQEVQKMLSYKNETGWSLLSKGPSVVVSGHGTTILKTVAEFEKWKEVVIKKGFAVTFKEYHQKIVGTTHRCSHLEIPNVAGKLPETIKCSDCPRVMEIFISYKCCHNENTANAIH

**26337163_peptide|Gmax_v1.1|Glyma20g12020|Glyma20g12020.2**

MSKSLSNTNNVQQGKTNLLQNPFEFSDEQILDNVYRTHFHCVEKCDVTSLHTVASCVINHSIEITDTVITKGSQLSDRFREDTTITSQQLTAKLKRIACQMVCTARGDHYAHHTTMLILEQLKAYSWDAKALIVQAAFALEYGKFLYLPLTTQYQMSEKSLADLNGLLMIQHNTQHLTFFNSVVKKVMQVIECITEWKRLTSAGYDIKDVPTLAETLHEIPVVVYWAIFTFVTCTGQIDDFTTDHKIHKHELSKNFENKLDLILRNFKEHLEMCGKEIGRIEDYTRRKNIVIHTGKDIVKVLKALIISGENRESRQSVFNILTGEQIKIEEFKKKHVLLFISGLDSIEEETLLLKSIYEKLKEKPREVEGYRKDDFKILWIPIVDEWNEERRKTLETKLQRTKFGWYVVKHFNFETGIKLIREVFNYKERSIIPLISPEGRVENIDTKQIISVWGIDGFPFRTSDHTRLTQQWNWFWSEMTKLNPRIGDLIEEDRYLFIYGGTDIMWIQEFTTAVEKLKRNVDSISLQIDITIESYQLGREDTKVVPRFWIAIDSLLASRKQQMMKGGDQGVQDFATREIKRLLFLKQDPKGWVILSKGYNVKLLGQGEAMCRSVRDFGIWYGKLHEEVSFDVAFKEYYESIKVKDCPKKCEHSEISNYPTDILAHIPCPNMECGRSMEVTSVNYRCCHGLEP

**26339105_peptide|Gmax_v1.1|Glyma20g11990|Glyma20g11990.2**

MSHSLSSTNVQEQEGMQNPFEFSEDKILDDVYRTHFDCFEKCDVTSLQTVASNVINHSIDISEKVISKMICTPRGEHFGHRTTMLILEQLKHYSWDAKVLIVQAAFSLEYGKIMYLPLTTQCQQQIENLFADLNGLLMVPQNTQHLPYFNSVVKKAMQMIECIIEWKRLISLGHDIKDVPTLAETFHQIPVVVYWAIFTFVSCTGQIDEFTDYKVQRHELSKSFEPKLDSILGKFKEFLERCSKEIVRIEDYTRREKIVIHTGKNIVKVLKALIISRENRDLRQNVFNVLTGEQVKIEEFKKYVLLFISGLDKIEDEIRLLKSIHEKLKEKPREVEGYRSEDFKILWIPIVDEWNEERRKKLESHLQCNKFGWYVVKYFNFETGMKLIKEVFKYKEKPIIALINPQGKVENIDTKQIISVWGIDGFPFRTSDHYRLTQQWNWFWSEMTKLNQGIESLIEEDCYLFIYGGMDTKWIQEFATAIETLKRDVAKLKLNINTTIESYQLGKDDPKAIPHFWIAIDSLLTRRKQMKKGIDFATSEEIKRLLFLKQDPKGWTILSKGHNVKLLGHGEAMCRTVKDFGMWHGKLHEEVSFDVAFREYYEEIMKDNKDCSKKCLNVISAGYAMDILERIVCPKKDCRRPMEVASVSYKCCHDRKK

**26339341_peptide|Gmax_v1.1|Glyma20g34660|Glyma20g34660.1**

MATKHSLMSLFHRTSSQGEHNPLNMSDEQILDQIYSTHVHSHTKFDVDSLFILVENTLRRSTLIVDNVVQGSKASLEQVEDKIPQANFNSPLYTLKQIYSEMSCKPPGEEIAHITTMAILVKLSNYEWDAKAVLTLAAFAMEYGEFWLLAQHQPTDPIAKSVAVLKGVPVLTRPAAVQKHRQAITELNNLVKTTLLVIELIFELEKLTTFDTKDVPALLPAIEQIPVDVYWAIITIVAIVTQIDYLTTESGNKQDLSHYGQKINIILSKLRKQIMLCRQQIEEAEYHHRLRKFFQTPTEIMEVFKFLVYSKDAPQLLFDGAAKTTVEITELKKKHVYLLISTLDITEEEISVLRPVYDSIKANDQYKIVWIPIVEEWTEKLHKKFEFLKSKMPWYVVQHSGPIAGYKYIKEEWHFKKKPMVVVLNPQGKVQHANAFHLIHVYGMKAFPFTIADQERIDREIHWIGSVVGDSHPHISTWIREQKYILIYGGSDKEWIHQFTKHATAFANDAALKDAKIHIELFCVEKEDKSFLRRFWSGIESLFVTKAHNTVDAVTQEVQKMLSYKNETGWAVLCKGSSVVMSGHGTTILKTVAEFEKWKEFVVKKGFEPSFKEHHERIRRTHHRCIHLEIPNAAGKLPETIRCPECGRIMEIFISYKCNHSDNTSIASN

**26339455_peptide|Gmax_v1.1|Glyma20g34720|Glyma20g34720.2**

MSLSNGDDSTVTSKQQKPQLPNPFDLTDSEILEKVYLTHLHDDDRCDVEVLSDIVSTVVLKTRLAEGKAYQTVFQPEYRTMKLISCQIITTPRGERYVHQTTMCILQHLRSYSWEAKALVTLAAFALEYGNLLHLSDVATPEKQLTNSLKQLNQVEARKKPATTLVELVMEVLHGIQEWTRLSGLDYDIVEVPSLTEAQQEVPVVVYWIIASLVAATANLVGLSEYKLSAFLERLSSAAYKFKEHLKSSLVQKGYADEYYKRRNAISKPKDIVEFLKLLIHHNGSKVQIYHGSIKTKTDIEVFSQKYVLLFISSLDKIEDEISLLNSIHDRLHENPNEVVKNYKKGDFKILWIPIVDAWDDTIKWYAVEFFTELPGTDLIKEKFNYLGKPIIPVLTPLGDIMNEDAMNLIFQWGIEAFPFRKIDGIELTLKWKWFWDATKKANLGIQQVTGDRYIFISRVAVEKTKGHATILNTDTIIDHYQLGKEDPTDVRRFWIEIERKRLKKHKDAVDCEIRKIVKTLLCLKQDQQGWAILTKGSNVRVLGHGEPMRQTLAEFDTWKEKVFQKEGFDVAFDEYYKTKLDELYARQECDFVKNNADVLVTIACPNPTCGRVMEVTSVNYKCCHRDATNNVNI

**26339798_peptide|Gmax_v1.1|Glyma20g34710|Glyma20g34710.2**

MALVLSKTASNGTTPQQKDQLPNPFELQDSQIRHKVYLTHVNDEKEFDRDVLFTLVSNTLNSASAQLSAAASSVTSFKPDFPTLKWLSCQMITTRGTPECAHQTALRILQQLSGFSWDAKALVAVAAFSLEYGEFLRLDRVQAADQFGNSLKQLNQVQISRRVPADMTDLVTVIGEVLNYINLWAKWSAMDYDIEAVHSLQVAMQEIPLVVYWTIASTVASIGNLVGISEHKLSAYKERLEFIFKKLQFHLENCRVEIGRIQDYCFRNTIRYPKLKDVVELLDILIIPGSDNGTSIPKIFEGGVLIKNGIEVFKQKYVMLFFSSLDNIGDEISLLNSINNGLQENPGEEIKGFKKGDFKILWIPIVDDWKTTREQFNNLKEKIKFYLVEYFEKLPGYDIIVDKFKYEGLPIVSVVNPQGQIMNDNAMQIIFEWGIDAFPFRRSDVYDLNKKWKWFWNLLEKTDDNAKRLGKDNTSYVFIYGGNDSSWVQNFKIAIGKIEKHVINNVDINIEPYQLGESNPDNVPSFWIGLDGKKKNKGCKDKVDCEIQEVVRTLLCLKQDPSGWVVLGRGRNLKILGHAEPMYQTVLDFDKWKSKVLEKETFDVAFKEYYDVVKEKYASLPYDHTSSVLATITCPNPLCGRVMEVTSINYRCCHGSANSCNL

**26345424_peptide|Gmax_v1.1|Glyma10g32950|Glyma10g32950.2**

MHHHPKHRAKHHLLSNTNLRGYYSQIGSPSMAQLSNGTSSTTLISKSGTTSIPHRASLPNPFDLTDDQILDIVYLAHLNDDETCDTDKLYNLVSNIVLRSQSPISAASFKPDFLTLKLISCQMISTRSAAHCVHQTTMWILQHLKCYSWDAKALIAIAALSLEYGSFVHLTQFQTNDVLGNSLRQLNQVQNRNASAVGELVMYVVQVFQHINEWATYAADGYDPEDVPDLTEAFQAILVVVYWSIASTVASTGNLIGVSNYKLSEYTFRLSTAVNKLTMHLTKVKEQIANVRDYITIRNIFDRPKDIVDLLKALIYPQQKGAENPKIFEGTNLVTKGIEVFRQKHVLLFISGLDSIEDEISLLNSIYERLQEDPREAKGFKKEDFKILWIPIVVKWSQSSREQFKALKSGTKFYAVEYFFELPGLKIIKDTERLNYEIQPIAPLFSSKGTLLNGNALEVIFEWGIEAFPFRKIDGDELTQKWKWLWDLILKATPGLQVKENRYIFIYGGANNTWVQNFTQELSKIKMNQSIQRADIIIENYQLGKGKGELNNSVPSFWIGVERKKQNKKHQEAVDCEIQKIVKCLFCLKRDPQGWAILSKGHNIKHLCHGQAVYQTVAEFQNWKEKVFEREGFDIAFKEYYDAKEKEISDTQPCEDYTSASSVIATIACPNPTCGRVMEVSSVNYKCCHRDDALNC

**26345444_peptide|Gmax_v1.1|Glyma10g32970|Glyma10g32970.1**

MAAKHSQVSLFHRTSSEGEHNPLNMSDEQILEQIYSTHVHSHTKFDVDSLFILVENTLRRSTLIVDNVVQGSKASSEQVEDKIPQANFNSPLCTLKQIYSEMSCKPQGEEIAHITTMAILVKLSNYEWDAKAVLTLAAFAMEYGEFWLLAQNQPTDPIAKSVAALKGVPVLTRPAALQKHRQAITELNNLVKTTLLVIELIFELEKLTTFDTKDVPALLPAIEQIPVDVYWAIITIAAIVTQTDYLTTELGNKQDLSHYGQKMNIILSKLRKQIMLCRQQIEEAEYHQRLRKFFQTPTEIMEVFKFLVYSKDAPQLLFHGATKTTVEITELKKKHVYLLISTLDITEEEISVLQPVYDSIKTGDQYKIVWIPIVEEWNEMLHKRFEFLKSKMPWYVVQHFGAIAGYKYIKEEWHFKKMPMVVVLNPQGKVQHANAFHLIHVYGMKAFPFTIADQERIDREIHWIGSVVGDNHPHISTWIREQKYILIYGGSDKEWIHQFTKYATAFANDAALKDAKIHIELFCVEKEDKSFLRRFWSGIESLFVTKAHNTVDAVTQEVQKMLSYKNETGWAVLCKGSSVVMSGHGTTILKTLAEFEKWKEDVVKKGFEPSFKEHHERIRRTHHRCIHLEIPNAAGKLPETIRCPECGRIMEIFISYKCNHRDNTSIAIN

**26345637_peptide|Gmax_v1.1|Glyma10g00260|Glyma10g00260.1**

MALVSSLSPATPSSVQTNTLNPLGWTDDQILEKVYITHVHTAERYDVESLFNVTANIIKRSTALADSVAVKTGTPVGLIEDKVPLSTFDPPFLKLKHIASQMMNTPHGEHHAHSTAMSILDQLRTYTWDGKAILVLAALALEYGNFWHLVQTPSGDHLGRSLAQMSRVHIVERNRQAVADYNSLVKNLLIAVECITELERLSTKGYDLKDVPALAEAMQEIPVAVYWAIVTTVVCANHFDFLLGESDSRYEIANFDDKLAAVISKLKANLTRSRKKIGDLEDYWRRKKLLQTPTEIVEVLKVLIYHNEVHDPHVYDGLTRQMVSIEVFRKKHVLLFISGLDSIRDEVRLLQSIYEGLQEDPREVKGYRKEDFRILWVPVVDEWNLLHRAEYDNLKLEMPWYVTEYFYPLAGIRLIREDLNYKNKPIIPVLNPQGRVVNYNAMHMIFVWGIDAFPFRPSDDDVLTQKWNWFWAEMKKVNPKLQDLIKADSFIFIYGGSDKKWLQDFAQAVERIKRHEIIKRADAVIEHYPFGREDHRIVPRFWIGIESLFANMIQKTHKDPTIEEIKSLLCLKQQQPGWVLLSKGSNVKLLGSGDPMLATAADFEIWKEKVLEKAGFDVAFKEYYEQKRRNYPQECSNMQLANYPADILHPINCPDAACGRSMEIASVSYKCCHGQAAHHKAEVPESGDVMIEKKLYAS

**26346195_peptide|Gmax_v1.1|Glyma10g32930|Glyma10g32930.1**

MSLSNGADSTATSKQQKPQLPNPFDLTDSEILEKVYLTHLHDEDKCDVEVLLDIVSSIVLKTRLAEGKASQTIFQPEFRTMKLISCQMITTPHGERYVHQTTMCILQHLRSYSWEAKALVTLAAFALEYGNLLHLSDVETPENQLTNSLKQLNQVQARKNPGTTLVELVMEVLHGIQEWSRLSGLDYDIVEVPSLTDAQQEVPVVVYWMIASLVAATANLVALSEYKLADFLDRLSSAADKFKEHLKSSVVQKGYADENYKRRKAFSNPKDIVEFLKLLIQHNGSKVQIYDGSIKTKTDIEVFNQKYVLLFISSLDKIEDEISLLNTIHDRLQENPNEVVKNYKKGDFKILWIPIVDTWDDKQKHKFNILKNTIKWYAVEFFTELPGTDLIKEKFNYLGKPIAPVLTPLGDRMNEDAMDLIFQWGIDAFPFRKIDGIDLTLKWKWFWDATKKANLGIQQVTGDRYIFISGGADKKWIQDFAVAVEKTRGHAIILNTDTIIDHYQLGKDDPTDVRRFWIEIERKRLKKHKDAVDCEIQKVVKTLLCLKQDQQGWAILTKGSNVRILGHGEPMRQTLAEFDTWKDKVFQKEGFDVAFDEYYKTKLDELYARQQCAFVKNNADVLVTITCPNPTCGRVMEVTSVNYKCCHRDASNNGNI

**26347074_peptide|Gmax_v1.1|Glyma10g32940|Glyma10g32940.1**

MAWVLSNTATSATTPEHKDQLPNPFELQDSQIRHKVYLTHVNDDKEFDRDILFTLVSNTVNSTSAQLSAATTSVTSLKPDFPTLKRLSCQMITTRGTPECAHQTALRILQQLSGFSWDAKALIAVAGFSLEYGEFWRLDRVQAADQFGNSLKQLNQVQISRRVPADMIDLVTVLGEVLSYINLWAKWSAMDYDTEAVHSLQAAMQEIPLVVYWTIASTVASIGNLVGISEHKLSAYKERLEFIFKKLQFHLENCRVEIGRIQDYHIRFNIRYPKIKDVVELLDILIIPGSDNGTSIPKIFEGGVLIKNGIEVFKQKYVMLFFSSLDSIGDEILLLNSINNGLQENPGEEIKGFKKGDFKILWIPIVDDWKSKREQFTNLKEKIKFYLVEYFEELPGYDIIMDKFKYEGLPIVSVVNPQGQIMNENALQIIFEWGIDAFPFRRSDVYDLNKKWKWFWNLLEQTDDNAKRLGKDNTSYAFIYGGNDSSWVQNFKIAIGKIEKHVINNVDINIEPYQLGESNPDNVPSFWIGLDGKKKNKGCKDKVDCEIQEVVRTLLCLKQDPSGWVVLSRGRNLKILGHAEPMYQTVLDFEKWKNKVLEKETFDVAFKEYYDVVKEKYASLPYDHTSSVLATITCPNPLCGRVMEVTSINYRCCHGSANSCNL

**26347097_peptide|Gmax_v1.1|Glyma10g00250|Glyma10g00250.1**

MSISNTQLKPLLPNPFNLSNTEIVEKVYVSHTYDDEMFDNEPLFNVVSNIIKLSTRIVGALLKIDEPNGFLGNPITISSFKPEFSTLKLMSCQMMTLPWGPENAHQTTLRILQQLRKYSWDAKALIALAAFALEYGNFWNLQQASDPLGNSLRLLNQIQHRQLPVTDINATVKLVMEAVEKIRRWGTLSSDETYETEDVPALSDALQLIPLLVYWVVASLVACNTNIQGVSNYALSDFRGKLSTALDEFKHHLEICEQQKASIEDYRRRKKAFKKPKDIVDFLKLLINQNGYKSQIYDGNANRNVNVEVFKEKYVLLFISGLDRIEDEIRLLNSIYERLVEDPNDKSGFKKEEFKILWIPIENKWGDARRELFNTLKSDIKWYVVEYAQVPLPGIRLIEEDLRFHGKPILPVVKPQGVLLNDDALDIIFEWGIHAFPFRKSDAYLLAQKWKWFWDEVKKTNLHGIQVKGDRYIFIYGGSDKWTREFTVAVDKIKRHDTIRRADAIIDYYHLGKDDPKIVPRFWIGIEGKRQKKHSENLDCEIQEIIRSLLCLKQDTQGWAILSKGSNVRILGHGQPMYQTVADFEKWKERVLVKEGFDIAFQEYYDTQRDLPAPQPCEFNTLDVLATITCPNASCGRVMEVTSVNYKCCHGGNVADHAIKSRSTTAT

**26347166_peptide|Gmax_v1.1|Glyma10g32980|Glyma10g32980.2**

MSIIMSSTKCLLQDAENEQNPLTMTDDQILEEIYSNHVGSDTKFDVDSLFTLVENTLRRSTHIVDNFVQGSHASLEHTDDKIPQFDSPLCTLKQISFEMSCKPPSDVIAHKTTLAILNNLKNYEWNAKAVLTLAAFALEYSEFWLLAQYQQSDPLAKSVAILKRVPVLTRQAALQKYRQAIVEVNDLVKATLQVIEQISVDVYWAIITIVSLTTRIDCLTTESEQKQELSHYGQKINIILSKLKKQITLCRQQIDAAEYYRKLRKLFQTPTEIMEVFKILIFNKDVPQPLYCGATKTMVDITVLKRKHVYLLISSLDITEEEISVFQTVYDSIKTSDQYEIVWIPIVEEWTVEYDNKFEDFKCKMPWYAVQHSGPIAGYQYIKEEWHYKSKPMVVVLSPQGKVQHSNAFHLIQAHGTRAFPFTTVKQEQINNETDWVGSVIGNIYPIINTWIKEKKYIFLYGGKDKEWIQQFTKNVSALASDAAITEANISIEWLCVEKEDRSVMRRFWGGIESLFVTKVHKAVDAVTLEVQKMLSYKNEAGWSLLISEGSSVVVCGHGKTITDTVEGFQNWKGSLTKKGFGLSFQGYHQKIVDITHRCSYLEISNVSGRLPETIKCPDCPRIMEIFVSYKCCHNNTIHY

**26351983_peptide|Gmax_v1.1|Glyma16g07300|Glyma16g07300.2**

MNSIGKLGAMQKLIKGGRAMPAAAISDDSVLVKKIVAEHSPEGIEYDVRPLLHIVEDVLIYSTLSSDSATTAALTRADHVEDRSHRPGHTNMLEALSAKIDRISCEISYKTLNGVDAHSTTIAIFDMLTIYKWDVKIVLALAAFALTYGEFWLLAQIHDTNQLAKSMAILKLLPSIMEHGSSLKPRFDTLNDLVNNILEVTKCVIEFHDLPAQYITQDISAYTTAYNYIPVASYWATRSIVACAAQITSLTTLGYEIFTSTDAWELSTLIFKLKNIVDHLRQLLNSCHEHIGKKMDAEAYQMLRELFSKPHTDNMKVLKALIYAQDDILPLYDGVTKKRVSLEPLRRKNVLLLFSGMEISTDELLILEQIYNESKAHAPRMESRYELVWIPIVDPNSEWIEPKQKQFEILQESMSWYSVYHPSLIGKPVIWFIQREWKYKNKPILVVLDPQGRVSCPNAIHMMWIWGSAAYPFTSSREEALWKEETWRLELLVDGIDQEILNWVKDGKYIFLFGGDDPEWVRRFVKEARRVATATQIPLEMVYVGKSNKREQVQKIIDTIIRDKLNTQYWSEQSMIWFFWTRLQSMLFSKLQLKQTDDDDHVMQEIKKLLSYDKQGGWIVLARGSHIVVNGHATTGLQTLVEYDAVWKELADRDGFEPAFKNHYDKVHSIVSPCCRFEFSHSMGRIPERLTCPECRRNMHVLTTFQCCHDEKIDEDFFVSTVTPPTI

**26775330_peptide|Graimondii|Gorai.004G261600|Gorai.004G261600.1**

MAATAASSLSYGIGGTQILWVPQGLLEQIVSTHSSYQTHHVDLQHLVNLISCIFQNATVSSSKGVEQYAQLTDMINKLETVDGMRQALENIRNLSCEMSCCVSTMASSVDETTMQMLRRLKDYSWNAKVVLAMAAFACSVAESSMLVKHRNTDPIAMYVETLKGHRYTTTDFTVLEHIGLFKAMIDVANTNLAFLAPSISRIPKEVHSIKDAIACFPTAAYKILRIVLQITSILSKKKDHFESTIQELNVLANEVSHINNILQEKLTLCLRDAEKYTYEDITIRITKISISEFIDKIMQYVRTQGFENLRNKHLLFLISDLDISIDEIKALNWLYQRNDQMYEIVWLPIIDLSMSYDVKRFWELKQLMKWSVAVEPTRVEADVVEFVKKEWHFIRQAIAVSMTSAGEVVCQNALPMLWTWGNTAFPFSDKTEEILWNSIDERHGWKLELVLDDFIVPELRSWIENRTTFVCLFGGGDISWIQEFTEKVKYAAYAAGVTLKLVYVGKNKAKLGLSKTDLSRDINVIESEFRWRFWTKLESILHAKIRRGKTTTAYKTDVVIHEALKVVGHGGKGESWAVFSMGPDPMVTTDGETGLTIMSNYLNWRQDTTGLRFLEGVKHYKEVISRDVHGCLKVHLPVLGRVPGIMVCPDCSKVMDMFYTYRCCDE

**26781599_peptide|Graimondii|Gorai.007G041500|Gorai.007G041500.1**

MATQSSHSQQLMRSERRMFSASDDSAMMKQIQSTHSPDGRFLDVKPILQVIDNVLRHLTPTIDHHALNGGQGHMDAIDDSSATMDSNGMLEALAFLVHKISCEISCKCSGGGDAHATTMVLLNTLSSYSWDAKVVLTLAAFAVNFGECWLVLQLCTTNSLAKSVALLKQLPDILEYSHTLKPHFDALHKLIKAMMEVTKCIVEFTELPSQYISSDVPPLSIAVAHIPTAAYWTIRSVVACAAQITSLVGMRYEFVTSTSEAWELSSLAHKVCSIHEHLQKILHLCHQHIDEKKQTEAYEGLKHSFGTPQLDNSKILLKIFSLSKEDPHSLLGPDKTKVHIEVLRRKHVLLLISDLDITHEEIQVLESLYKYDRVASEVNYEIVWLPIVDRSAWNDSYQQKFLSLQSMMPWYTVNHPSIIEPAVVKYTREVWNFVKKPIVVTLDPLGKMTCPNALNMLWIWGNTAFPFTTDREASLWKAEAWTVELLVDGLEPNLSNWVRQEKVICLYGGEEMEWIESFTSATKNVAQFLGIGLEMVYVGKNNAKERVKKITGLINERQLSHAWQDANVWFFWKRLESMLFSKTQQGKTNEIDVIKQEVMTLLGYDGSEQGWAVFFSGTNMVRAKGDKVLNAIQSFEQWEDMARVSGFISAIRGHLEGIADEHHCTRLILPGISGGIAETVVCAECGRTMEMYFMYRCCDE

**26797563_peptide|Graimondii|Gorai.003G177900|Gorai.003G177900.1**

MAAPISLSLSSLASKSQQLVRNERRMFVTSDDGAMMKQIQSSHAPDGRVVDVKPILQVIENVLHHIIPNVDHAMNGGTGHIDALDDRKNSSAVGGALDALAYIVHKICCEVSCKCSGGGDVHATTMGILNMLSSYSWDAKVVLTLAAFAVNFGEFWLIVQLCTSNSLAKSVALLKQLPDLLEHSPTLKPHFDALHKLINAMIDVTKCIVEFTELPSEFISIDVPPLSTAMAHIPTATYWIIWSVVACAAQITGLAWELSSLAHKVSSIHEHLQSLLRLCYQRIDEKKLMEAFEDFKRTIETPQMDNLKILLKIFRKEETCYLMNPDKTEVLIDVLRRKHVLLLISDLDISHEEIRVLEALYKGERVSSELNYEILWLPIVDRSTWNDGFEQKFLSLKSIMSWYTVKHPFAIEPAVIKYIMEVWGFVKKPIAVTLDPQGKVLCPNALNMMWIWGNSAFPFSSEKEESFWKAEAWTLELLVDRLEPNLPTWVSQQKVVCFYGGVQMEWIESFTTATKEVAKALDIGLEMVYVGKNNAKERVKKITGLIKEKQLSHAWEDDNVWFFWNRSMLYSKTQHGKTIENDVIKQEVMTMLAYDGSENGWAVFFTGSYEMVRANGDKVLSSMKSFDEWEKLAKQMGFIPAFRKHLDGITDDHHCTRLILPGNGGRIPERVQCAECGRPMEMYFMYRCCVE

**26797654_peptide|Graimondii|Gorai.003G107900|Gorai.003G107900.1**

MAQASRSQQLVRSERRMFAASDDNAMMKQILSTHAPDGRLVDVKPILLIIDNVLRHITPEIDQALTAGSGRIDSFDDQTNLSAIDDVLDALAYIVHKISCEISCKCAGGGDAHATTMVILNMLSSYSWDAKVVLTLAAFAVNFGEFWLIVQLCTTNTLAKSVALLKQLPDVFEHSQTLKPHFDALNKLIKAMIDVTKCIVEFIELPCEYISSEVPPLSTAMASIPTAAYWTIRSVVACAAQITSLVGLRQEFVTSTSEAWELSSLAHKVSSIHEHLQNLLRLCYQRIEEKKQEETYKEFIRIIETPQMEISKILRVIFRKEDPHPLFSPADKTRVDIDVLRRKHVLLLISDLDISLDEIQVLEVLYKYERASSSELNYEIVWLPIVDRSAWNDSYQQKFLNLQSIMPWYTVNHPSVIEPAVIKYTKEKWRFVKKPIVVTLDPLGKVTCTNALNMMWIWGNAAFPFSTDKEESLWKSESWTIELLVDGLEPNLPNWMREEKVICFYGGEKMEWIESFTSATKKAAQTLEIGLEMVYVGKNNAKERVKKISGLITEKQLSHSWQDASVWFFWNRLESMLYSKTQHGKTNDPDIIKQEVMTILGYDGSEHGWAIFFLGTTEMVRANGERVLSSMQSFEEWEEMARQMGFIPALRKHLEGITDDHHCTRLILPGISGGIAERVVCAECGRPMEMYFMYRCCVE

**26797831_peptide|Graimondii|Gorai.003G177000|Gorai.003G177000.1**

MARPTPISSQQSMRNEHWMFDDAMNERIRSTHAPDGRVVDVTQVLQVTRNVLRHIIPNINLSLNGHIDASDDQTNLSAADGALDALHKICCELSCKCSRGGDAHATTMAIFNMLSSYPWVAKVVLTLAAFAVNFGEFWLIAQLCTSNPLAKSVALLKQPDISEHSQTLKSHFDALSKLINAMFDVTKCIVKLTELRSSKYISIGEPPLSTAMAHIHTATYWIIASVVACTRQITGLIEFTTVTSEAWELSSLAHKVSSIHEHLQSQLSLCYERIDEKKLIEAFEHFKRTIETPQVDNLKILQNIFGKEENLLNPDRAEVCINVLGRKHVILLISDLSISQEEIRVLEDVYKERVSSGLNYEIIWLPIVDRTTWNDYYREKFSKLQSIMSWYTVSQHVAIEPAVIKYIREEWGFFKKPIAVTLNPQGKVLCPNALNMMWIWGNSAFPFSSEKEESFWKAKPWTLDLLFGRLEADLPTWVSQQKVVCFYGGVKMEWIQSFTTATTAVAEALGIGIEMVYVGKKNKRERVKKITGLIKEKELSRAWEDDNVWFFWNLLESMLYSKNQHGKTIENDVIKQEVMTMLEYDSSKNGWAVFYTGSGEMVKANGEKVLSTMDKFDEWKNLAKQMGFVPALREKLEGVIPRHHCTRLILPSNGGRIPERVQCAECGRPMELNFLYRCCAE

**26799210_peptide|Graimondii|Gorai.003G177800|Gorai.003G177800.1**

MATPISLSISSLASKSQQMVRNERRMLAASDDGAMMKQIQSTHAPDGRVVDVKPILQVIDNVLRNIIPNIDHAMNGGTGHIDALDDRTNSSAVDGALDALAYIVHKICCEVSCKCSGGGDAHATTMGILNMLSSYSWDAKVVLTLAAFAVNFGEFWLIVQLCTSNSLAKSVALLKQLPDILEHSQTLKPHFDALNKLINAMIDVTKCIVEFTELPSEFISIDVPPLSTAMAHIPTATYWIIWSVVACAAQITGLVGMRHEFITSTSEAWELSSLAHKVSSIHEHLQSLLRLCYQRIDEKKLLEAFEDFKRTIETPQMDNLRILLKIFRKEETYYLLNPDNTKQVLIDVLRRKHVLLLISDLDISHEEIRVLEALYKGERVSSELNYEILWLPIVERSTWNDDYEQKFLSLKSIMSWYTVNHPFAIEPAVIKYIMEVWGFVKKPIAVTLDPQGKVLCPNALNMMWIWGNSAFPFSSEKEESFWKAEAWTLELLVDRLEPNLSTWVSQQKVVCFYGGVQMEWIESFATATKGVAKALDIGIEMVYVGKNNARERVKKITGLIKEKKLSHAWEDGNVWFFWNRLESMLYSKTQHGKAIENDVIKQEVMTLLAYDGSENGWAVFFTGSDEMVRANGDKVLSSMKSFEEWEKLAKQMGFIPALRKHLEGITDEHHCTRLILPGNSGGIPERVQCAECGRPMEMYFMYRCCVE

**26800011_peptide|Graimondii|Gorai.003G178000|Gorai.003G178000.1**

MATPISLLLSSLASKSQQLVRNERQMLAASDDGAMMKLIQSTHAPDGRFVDVKPILQVIVNVLHHTIPNIDHAMNGGTGHIDALDDRTNSSAIDGALDALAYIVHKICCEVSCRCSGGADAHAATMGILNMLSSYSWDAKVVLTLAAFAVNFGEFWLIVQLCTSNSLAKSVALLKQLPDILEHSQTLKPRFDALNKLINAMIDVTKCIVEFTELPSEFISIGVPPLSIAMAHIPTATYWIIWSVVACAAQITGLVGMRHEFITSTSEAWELSSLAHKVSGIHEYLQSLLRLCYQRIDEEKLMEAFEDFKRTIETPQTDNLKILLKIFRKEETYYLLNPDKTKVLNDVLRRKHVLLLISDLDISQEEIRVLEALYKGERVSSELNYEILWLPIVDRSTWNDGYEQKFLSLQSIMPWYTVNHPFAFEPAVIKYIMEVWGFVKKPIAVTLDPKGKVLCPNALNMMWIWGNSAFPFSSEKEESFWKAEAWTLELLVDRLEPNLPTWVSQQKVVCFYGGVQMEWIESFTTATKGVAKALDIGLEMVYIGKNNAKERVKKITGLIKEKQLSHAWEDDNVWFFWNRLESMLYSKIQHGKTIENDAIKQEVMTMLAYDGSENGWAVFFTGSDEMVRANGDKVLSSMESFDKWEKLAKQMGFIPALRKQLEGITDDHHCTRLILPENSGGIPERVQCAECGRPMEMYFMYRCCVE

**26802772_peptide|Graimondii|Gorai.005G175200|Gorai.005G175200.1**

MELSSGFYGRSNPHMLSTSGGKAMGKLIEATHEPAGVEINMKPVLDIVEDIFERATPAAPGAIKQVDASEPDERALHYSSLEQMMDVLSATINRISCEITYRLSIGEDAHSTTLAVAQIVKGYSWDAKIVLALAAFAMTYGEFLLVVQLYATNPLAKDVALLKQLPEILTRADLLKPKFDTLANLINAMAVVAKCTVEFKGLPSKYIRPEDPEMSSANADIPSAVYWTIRSTVICSSQIMGLINMGHEFLSSTTDAWELSSLTHNINSIYNDLVEKLNRCRQRINERKDIEAFETLVRLFDAVHIDNMKILKALIYAKDDQPPLWDGTTKQRVSIELLRRKSVLLLISDLQIPEKELIILEQIYNESRVQPTRIESQYEVIWIPIVDRSSTFDDTMRKQFESLQAMMPWYSVGHPSMIQPAVMRYIKEVWGFNKKPLLVVLDPQGRVVNPNAMPMMFIWGSMAFPFTKLREEALWKEETWRIELLADSIDPSIINWLTDRKFICLYGGEDMNWIRKFTTTAKAVAKTANIELEMLYVGKSNPRERVRRNMTNIELENLSHTLSDISLIWFFWVRLESMWHSRAQHGVTVRNDLIMQEILTMLGFDGSDQGWAVISRGADEMARAKAETFLKSLEEYTAWEAAAAEKGFIPALNDHFRSLRTEHHCNRLTLPGISVAEIGSIKDTVVCVDCGKPMEALLMFRCCTD

**26806791_peptide|Graimondii|Gorai.011G093700|Gorai.011G093700.1**

MAHLTNPGPFHPSIPRPLHNPASAPAPAAAQPQVHDHGAAPANHNHDHLTSTTQLRTATPGASQPLMKSPSATHALIRGDRTAGMLTMSDDHVMMNQILATHAPDGRDVDVRPLLYLVEDILNRATEHVDFIIKGTQAQIEMEEKAQQANYIAMLEALTFTIDRIACELSYKALRGSDPHATTTAIFSFLSSYAWDAKLVLSLSAFALNYGEFWLLAQIYSTNQLAKSMAILKQLPSLLEHTAPLKPRFNALNTLIRTMMDVTRCVVEFKDLPSMYISPDAPALASAMTLIPTAVYWTIRSMVACATQISSLTSMGHEYGVSATESWELSTLAHKLQNIHEHLRKQLNLCYQHIEEKKDVDTFHMLLKLFDPNVAHIDNMKHLKALIYAGDDKLPLLDGATRRKAGLEVLRRRNVLLLISSLEFSGDELAILDQIYSDTRMHEAGMQSHYEVVWIPVVERSGVPLSEETPTRFEALRSSMPWYSVEDPFLIEKPVMRFIKEVWHFRTKPILVVLDPRGKVLSQNAIHMMWIWGSAAFPFTSYEEEKLWSKETWQLNLLIDGIDPLILDWIKDGKYIFLYGGDDMEWISKFVASARTVASAFGIPLEMVYVGKSNKREQVKKVAAIIKERKLSHCWEDPAMVWFFWTRLESMMFSKIQLGRADDLDPIVQGIKKLLSYGREGGWAVLSEGSNILVNGHSTTVLPTLVDYVKWRGNVAEKGFNLSFIEYHDQLHNVAHPCCRFEFLATTKAPESIRCPECHRVMEKHTAFVCCHDEQAIPDSLFAS

**26826991_peptide|Graimondii|Gorai.012G066200|Gorai.012G066200.1**

MQQSDRGLRLSFSTSYDGLMLKQIQAVHVPDGRAIDVRPLLHIVEDILSFAAPSGDAIVETGKQATGTEAFQHQTNYQTNITDMLETLSFLIDRISIEMARKCSETREEHATTMSILSMVSNYPWDAKLVIALSAFAVNYGEFWLLAQCYTSNQLAKNLAILKQVPGILQHSTMLKSRFDTIKDLITAMLDIAKCLVEFKEIPSNYVTEDVAAAVSAAMDHIPVAIYWTIRSMLATASQITGLSGSENEFLSSLESWELSSLVHKLSTMHSHLVGLLAICHKQIDERKFIEAYQNLQYLFNAAQIDNIKVLKALINPKDDPLPLVDGANKKRVSVDVLRKRNVLLLISDLDILQDEVVILQQIYEESRRQSNSLDQNPYELVWLPVLDSSVSLSKIKQRIFENLTATMTWFTLRHPALLNRAVFKFIKEEWGFEQKPIVVVLDPQGRVTCSNAIHMMWIWGNLAFPFTIAKEDALWKAETLTLDFLVDGIDPVILKWISEERFIFLYGGEDIEWIRNFTHTVKTAARACGIAMEMVYVGKRNPKENIRRNMAIINEEKLSHCLPDITAIWYFWIRIESMWNSKHQLGKADENDPTTQEIMTLLSYDGGEGYSQQRCLTSDLLSNSLFFTTGTSA

**26827035_peptide|Graimondii|Gorai.012G028700|Gorai.012G028700.1**

MAAETKTPPASRYYSFDKSSQQIQMTNKTNSFQTLEPDVGQHLLDLIQSIFQVVVASKDIDQSAELNKMISSLETFQGMRQVLNDIRDISCEMSCNLLAAGSGNVEKTRTAVLERLRSYSWSAKVVIAIAAFASSIGELSMLVKHRDDEPMAKFLVNILKGYSPKLDLNALAEAKLIEGMLKVVRTNLDFSNRLKQESMKEAMLELYLNATKNIFDIVLQISTVLSRREDVHVELESLSKQLTEINIQLQDNMKYFGAERISPNWYEEVSEISTSELIAKIKKYIEVRDSEKLRNKHVLFLISDLNISIEEIKVLERLYEKNEGKYEIVWLPIVGSLAYDKKAAARFWELRQMTKWIGVEPSRIEEEVIQYIKRDWHFIKEAIAVSVNEVGEITCLNALPMLWAWGNRAFPFTDKITLWNELDERNGWTWRLDLLFDQLILPGDSGIDIQSWTKSEGTLVCLFGGGDISWNQEFIQKVKYAARSAGVTLKLVYLGVGKNKGKGLTRNQLGRDVLVIQSESQWQFWSRLESILYAKIRLGKKDEVMQEALKVVGYGGNGEEWAMFSMGEGAMVTTSGKTALTIMEDYQMWRSDMIGVRFLEGIKKYKELITRDVHSCINLHLPVMGQIPGIMICPECSKVMDVFYTYRCCPE

**26827339_peptide|Graimondii|Gorai.012G066300|Gorai.012G066300.1**

MDQQKKTTSGALQAPLTAPSNNPTAAAQALLHNPGAAQALANNPGAAAAAEAILGNRGATYQPLLNNTHVGQQHMGVPGASISKPSTGEALMKGHTGSHALIRSDRGSMLSLSDDNVMMKQILATHAPDGREFDVRPLLNLVEDILNRASQHVDFLVKGTTQAQIEMEEKALQANHIVMLEALTYTIDRIASELSYKALGGSDAHATTTAIFNLLSSYTWDAKLVLSLSAFALNYGEFWLLAQIYSTNQLAKSMAILRQLPSLLEHTAPLKPRFDALNNLIRAMIDLTKCVVQFTQLPSTYISQDVPVLETAINLFPTAVYWTIRSMVACATQISNLSSMGHEFGISTTESWELSTLAHKLRNIYELLKQQLNLCYQYIEEKTDVAFYQLLLTLFDPNALHIDNMKVLKALIYDKDDKLPLLDGATKRRVSLDVLRRKNVLLLISSLEFANDELAILEQIYNESRIHATRLESQYEVVWIPIVDHSIIPLPEEMQTKFENLQSTMPWYSVQDPLVIKKPVIRFIKEVWHFRTKPILVVLDPQGKVVSPNAIHMMWIWGSTAFPFTSLREEALWREETWRLDLLVDGIDPTVLNWIKEEKYIFLYGGDDVEWVRRFANSARSVASASRIPLEMVYVGKSRKREHVKKVVGIINAEKLSYAWQDPTMVWFFWTRLESMLFSKIQLGRADDQDPMMQQIKKLLSYGREGGWAVLSRGSNIVVNGHSTTVLPTLGGYDEWKVNIAELGFDMAFKEYHDKLHDVAHPCCRFQFPTIIRTPENMRCPECHRVMERYTSFICCHDDKGIPGSLF

**26828315_peptide|Graimondii|Gorai.012G066400|Gorai.012G066400.1**

MGINTQAFVFPYIYIYCLCFSVTYSMDQHKTTAGALQTPMTGPINNPAALAQALLHNPGATQALNNNPTAAGNHGVTHKPHLNNPGVGQQLISYPGVGQQHINNPGVGQQLISYPGVGQQHISNPGVGQLMGVPGSGQGSINKPSTAQALMRSPSDSRALIRGDRGSMLSMSDDNVMMNQILTTHAPDGREFDVRPLLYLVEDILNRATQHVDFLVKGTLDQVELEERAQAANHIAMLEALTYTIDRIACELSYKALGGCDAHATTTAIFNLLSSYTWDAKLVISLSAFALNYGEFWLLAQIYSTNQLAKSMAVLRQLPSLLEHTAPLKPRFDALNSLIRTMMDVTRCVVQFRELPLKYISPDAPALATAMTHIPTAVYWTIRSMVACATQISSLTSAGHEFGISTTESWELSTLAHKLRNIYEHLKQQLNYCYKHIEERMDIEIYQMLLAIFDPNVLHIDNMKALKALIYDKDDKLPLLDGATKRQVSLDVLRRKNVLLLISSLEFSNDELAILEQIYNESRIHATRLESQYEVVWIPIVDHSIIPLPEEMQTKFENLQSTMPWYSVQDPLVIKKPVIRFIKEVWHFRTKPILVVLDPQGKVVSPNAIHMMWIWGSTAFPFTSLREEALWREETWRLDLLVDGIDPTVLTWVKEEKYIFLYGGDDIEWIRRFVNSARGVASASRIPLEMVYVGKSNKREQVKKVTGIINAEKLSNAWQDQAMVWFFWSRLESMLFSKIQLGRGDDQDPMLQQIKKLLSYGREGGWAVLSRGSNIAVNGHSSTVLPALGGYDEWKINVAEKGFDGAFKDYHDKLHDAAHPCCRFEFPNTIRIPENMRCPECPRLMEKYTAFLCCHDEQGIPGSLF

**26979242_peptide|Ptrichocarpa|Potri.010G050600|Potri.010G050600.1**

MAVVPQKMRRERSMFSSSDDTAMMKQIQATHAPDGRGREFSVKPLLHIVEDIFLRSTPALGMTSIVQQQGAHQAQLDELEEKALQNGFHETIEMLSYTINKISCEMSCKCSGGGDAHATTLAIFNLVSNYSWDEKVVLALAGFAVNYGEFWLVAQLYLTNPLAKAVALLKQLPDIIERADNLNPKFEELTTLIKAVMDVARCIFEFKELPSQYITPDTPEMLTATAHIPTAVYWTIRSVVACASQVMGLIGMGHEYIASTTEAWELSSLAHKVNNIHSHLMKQLTLCFQHIDEKRHIEAFQTLVSLFEAFHIDNMKILKALIYAKDDQLPLFDGSTKKRASLDVLRRRSVLLLISDLEISHEELSMLQQMYSEAREQPGRPESQYEVVWLPVVDRSSPWSETKQKLFEDFQRIMPWYSVHHPSLLDVAVIRYIKEVWHFNKRPLLVVLDPQGRVVNPNAIHMMWIWGSLAFPFTSLKEEALWKEETWQIELLADSIDPLILYWIDQGKYICLYGGEDIEWIRKFTATAKEVASKAAITLEMLYVGKCNPREKVRENNVIIKNEKLSHVLPDLTLIWFFWVRLESMWHSKVQLKRTVENDAIMQEIMTMLSFDGSDQGWAVISRGPADMAKAKGETILKSFVDFETWRDGAQEKGFLPALIDNLLALHSPLHCNRLILPGATGSIPEKVVCAECGRPMKKFIMYRCCTD

**26981034_peptide|Ptrichocarpa|Potri.010G050300|Potri.010G050300.1**

MAVVPQKMRRERSMFSSSDDTAMMKQIQATHAPDGREFPVKPLLHIVEDIFLRATPALGMTSIVQQQGAHQAQLDELEEKALQNGFHETIEMLSYNINKISCEMSCKCSGGGDAHATTLAIFNLVSNYSWDEKVVLALAGFAVNYGEFWLVAQLYLTNPLAKAVALLKQLPDIIERADNLKPKFEALTSLIKAMMDVAKCIVEFKELPSQYITPDTPEMLTATAHIPTAVYWTIRSIVACASQIMGLIGMGHEYIASTTEAWELSSLAHKVNNIHSHLMKQLTLCLQHIDEKRHIEAFQTLVSLFEAFHIDNMKILKALIYAKDDQLPLFDGSTKKRASLDVLRRRSVLLLISDLEISHEELSMLQQMYSEAREQPGRPESQYEVVWLPVVDRSSPWSETKHKLFEDFQRMMPWYSVYHPSLLDVAVIRYIKEVWHFNKRPLLVVLDPQGRVVNPNAIHMMWIWGSLAFPFTSLKEEALWKEETWKIELLADSIDPMILSWIDQGKYICLYGGEDIEWIRKFTVTAKDVASRAGITLEMLYVGKSNPREKVRKNNSIITTEKLSHVLPDLTLIWFFWVRLESMWHSKVQHKRTVENDAIMQEIMTMLSFDGSDQGWAVISRGPADMAKAKGETILKSFVDFEIWKEGAQEKGFLPALIDYLHELHTPFHCNRLILPGATGSIPERVVCAECGRPMEKFIMYRCCTD

**26982218_peptide|Ptrichocarpa|Potri.010G050500|Potri.010G050500.1**

MLSYNINKISCEMSCKCSGGGDAHATTLAIFNLVSNYTWDEKLVLALAGFAVNYGEFWLVAQLYLTNPLAKAVALLKQLPDIIERADNLNPKFEELTTLIKAVMDVARCIFEFKELPSRYITPDTPEMLTATAHIPTAVYWTIRSVVACASQIMGLIGMGHEYIASTTEAWELSSLAHKVNNIHSHLMKQLTLCFQHIDEKRHTEAFQTLVSLFEAFHIDNKMILKALIYAKDDQLPLFHGSTKKRASLDVLRRRSVLLLISDLEISHEELSMLQQMYSEAREQPGRPESQYEVVWLPVVDRSSPWSETKQKLFEDFQRIMPWYSVHHPSLLDVAVIRYIKEVWHINKRPLLVVLDPQGRVVNPNAIHMMWIWGSLAFPFTSLKEEALWKEETWKIELLADSIDPLILSWIDQGKYICLYGGEDIEWIRKFTVTAKDVASRAGITLEMLYVGKSNPRETVRKNNTIITTEKLSHVLPDLTLIWFFWVRLESMWHSKVQHKRTVKNDAIMQEIMTMLSFDGSDQGWAVIGRGPADMAKAKGETILKSFVDFEIWKEGAQEKGFLPALIDYLHELHTPFHCNRLILPGATGSIPERVVCAECGRPLEKFIM

**26982854_peptide|Ptrichocarpa|Potri.010G050800|Potri.010G050800.1**

MATHAPDGRERSMFSSSDDTAMMKQIQATHAPDGREFSVKPLLHIVEDIFLRAAPALGMTNIVQQQGAHQAQLDELEEKALQNGFHETIEMLSYTINKISCEMSCKCSGGGDAHATTLAIFNLVSNYTWDEKLVLALAGFAVNYGEFWLVAQLYLTNPLAKAVALLKQLPDIIERADNMKPKFEELTSLIKVMMDVAQCIFEFKELPSQYITPDTPEMLTATAHVPTAVYWTIRSIVACASQIMGLSGKGHEYIASTTEASELSSVAHEVNNIHSHLMKQLTLCFQHIDEKRHIEAFQTLVSLFEAFHIDNMKILKALIYAEDDQLPLFDGSTKKRASLDVLRRRSVLLLISDLLISHEELSMLQQMYSEAREEPGRPESQYEVVWLPVVDRSSPWSETKEKLFEDFQRIMPWYSVHHPSLLDVAVIRYIKEVWHFNKRPLLVVLDPQGRVVNPNAIHMMWIWGSLAFPFTSLKEEALWKEETWKIELLADSIDPMILSWIDQGKYICLYGGEDIEWIRKFTVTAKDVASRAGITLEMLYVGTSNPREKVRKNNSIITTEKLSHSMWHSKVQHKRTVENDAIMQEIMTMLSFDGSDQGWAVISRGPADMAKAKGETILKSFVDFEIWRDGAQEKGFLPALIDHLHELHTPFHGNRLILPGATGSIPERFVCAGCGRPMQKFIMYRCCTD

**26984048_peptide|Ptrichocarpa|Potri.017G071000|Potri.017G071000.1**

MEARKLIQGSFIPFAMTHKPIFSTSEPLPLAYYQLNMASGLPFGLPAPSASTMNQQLIRSDRSMITMSDDNVMMKRIVETHAPDGREVDVKPLLHLVEDILKRATLQTDTSLTTSQAHAESEDKTNHASFAVMLDSLSYTIDRISCEIAYKGGADGHATTVELFNMLASYSWDAKLVLTLAAFALNYGEFWLLAQIYSSNQLAKSMAILKQLPNILEHSGPLKPRFDALNNLIKVMMDVTRCVVEFKDLPPTYISHEVSALSAAMAHVPTAVYWTVRSVLACAAQITSLTTMGYEFSISTTKAWELSTLAHKLSNILEHLRRQLATCYQYIDEKRNVEAYQMLLNLFEMIHIDNMKVLKALIYAKDDIQPLIDGSNKRRVHLDVLRRKNVLLLISGLDISNDELAILEQIYNESSHHGTRLDSQYDLVWIPITDHSVQWTDPLKEKFESLQNSMPWYTVYHPSLIDKAAIRFIREVWHFRNKPILVVLDPQGKVVSPNALHMMWIWGSNAFPFTSLREESLWREETWRLELLVDGIDPVILNWIKEEKYIFMYGGDDVEWVRKFTNTARAVAQAARIPLEMVYVGKSRKREQIRRVMGTINVEKLSYAWQDLTMIWFFWTRLESMLFSKIQLGKVDDHDPMMQAIKKLLSYDREGGWAVLSKGSSVVVNGHGTTVLPTLVEYDLWKEQVPVKGFDLAFQEHHGNLHDIVHPCSRFEFPMTAGRIPETLKCPECNRSMEKFTTFLCCHDVLLQ

**26993278_peptide|Ptrichocarpa|Potri.T123700|Potri.T123700.1**

MTSMAVVPQKMRRERSMFSSSDDTAMMKQIQATHAPDGREFSVKPLLHIVEDIFLRATPALGMTSIVQQQGAHQAQLDELEEKALQNGFHETIEMLSYNINKISCEMSCKCSGGGDAHATTLAIFNLVSNYSWDEKVVLALAGFAVNYGEFWLVAQLYLTNPLAKAVALLKQLPDIIERADNMKPKFEALTSLIKAMMDVATCIVEFKELPSQYITPDTPEMLTATAHIPTAVYWTIRSIVACASQIMGLIGMGHEYIASTTEAWELSSLAHKVNNIHSHLMKQLTLCFHHIDEKRHIEAFQTLVSLFEAFHIDNMKILKALIYAKDDQLPLFDGSTKKRASLDVLRRRSVLLLISDLEISHEELSVLQQMYSEARELPGRPESQYEVVWLPVVDRSSPWSETKQKLFEDFQRIMPWYSVYQPSLLDVAVIRYIKEVWHFNKRPLLVVLDPQGRVVNPNAIHMMWIWGSLAFPFTSLREEALWKEETWKIELLADSIDPMIVSWIDQGKYICLYGGEDIEWIRKFTVTAKDVASRAGITLEMLYVGKSNPREKVRKNNSIITTEKLSHVLPDLTLIWFFWVRLESMWHSKVQHKRTVENDAIMQEIMTMLSFDGSDQGWAVISRGPADMAKAKGETILKSFVDFEIWRDGAQEKGFLPALIDNLLALHSPLHCNRLILPGATGSIPEKVVCAECGRPMEKFIMYRCCTD

**26993282_peptide|Ptrichocarpa|Potri.T123900|Potri.T123900.1**

MATHAPDGRERSMFSSSDDTAMMKQIQATHAPDGREFSVKPLLHIVEDIFLRAAPALGMTNIVQQQGAHQAQLDELEEKALQNGFHETIEMLSYTINKISCEMSCKCSGGGDAHATTLAIFNLVSNYTWDEKLVLALAGFAVNYGEFWLVAQLYLTNPLAKAVALLKQLPDIIERADNMKPKFEELTSLIKVMMDVAQCIFEFKELPSQYITPDTPEMLTATAHIPTAVYWTIRSIVACASQIMGLSGKGHEYIASTTEASELSSVAQEVNNIHSHLMKQLTLCFQHIDEKRHIEAFQTLVSLFEAFHIDNMKILKALIYAEDDQLPLFDGSTKKRASLDVLRRRSVLLLISDLEISHEELSVLQQMYSEAREQPGRPESQYEVVWLPVVDRSSPWSETKEKLFEDFQRIMPWYSVYHPSLLDVAVIRYIKEVWHFNKRPFLVVLDPQGRVVNPNAIHMMWIWGSLAFPFTSLKEEALWKEETWNIELLADSIDPMILSWIDQRKYICLYGGEDMEWIRKFTVTAKDVASRAGITLEMLYVGKSNPREKVRKNNSIITTEKLSHVLPDLTLIWFFWVRLESMWHSKVQHKRTVENDVIMQEIITMLIFDGSDQGWAVISRGSADMAKAKGETILKSFVDFERWKDGAKENGFLPALIDYLHELRIGATGSIPERVVCAECGRPKENLSYILYS

**27003362_peptide|Ptrichocarpa|Potri.T067300|Potri.T067300.1**

MASGLPFRHPAQGFNASQQLIKSDRGSMLTMSDDNVMMKQIVGTHAPDGREVDVKPLLHLVEDILKRATQQIDTSLTTSQAHVELEDKTHQVNFVSMLDALSYTIDRISCEIAYKSLDGTDAHATTVSLFNMLPSYSWDAKLVLTLAAFALNYGEFWLLAQIYSSNQLAKSMAILRQLPSIMEHSGPLKPRFDAINNLIKVMMDVARCVVEFKDLPPAYISNEVPALSTAMAHIPTAVYWTMRSVVACAAQITSLTTMGHEFSISTTVAWELSSLAHKLSNILDHLKTQLATCYQHIDEKRNVESFRMLKNLFEMVHIDNMKILKALIYAKDDIQPLIDGSSKKRVHLDVLRRKNVLLLISGLDMSNDELSILEQIYNESRPHEARLESQYEVVWVPIVDRSVQSDAMKEKFESMQSSMPWYTVYHPSLIEKAVIRFIKEVWHFRNKPILVVLDPQGKVVSPNALHMMWIWGSSAFPFTSLREESLWRDETWRLELLVDGIDPVILNWIKEGKYIFLYGGDDDEWVRKFTNTARAVAQAARIPLEMVYVGKSSKREKIRRVIATITVEKLSYVWQDLTMIWFFWTRLESMLYSKIQLGRLDDHDPMMQEIKKLLSYDREGGWAVLSKGSNVVANGHRTTVLQTLLEYDMWKDQVPVKGFDLAFQDHQGRIHDISRPCCRFDFPMTTGRIPETMKCPECNRTMEKFSTFLCCHDEVIPDELFK

**27038708_peptide|Ptrichocarpa|Potri.008G183200|Potri.008G183200.1**

MAGVPQKTRRERTVFAASDENVMMKQIQATHAPDGREFSVKLLLQIVEDIFHRATPAPGITDFVQHQGSHQAQLYELEEKVLQNGFNEMIDMLSHTISKISCEMSCKCSGGGDAHATTLAIFNLVSNYSWDAKVVVALAAFALNYGEFWLVSQLYLTNPLAKAVALLKQLPEIIERAEALKPKFEALTNLIRAMTDVAKCIVEFKELPSQYITPDTPEMLTATAHIPTAVYWTIRSIVACTSQIVGLTGMGHEYIASTTEAWELSGLAYKVSNIHSHLVKQLTLCFQHIDEKRHHEAYLTLVRLLESVHIDNMKILKALIYAKDDQLPLFDGSTKKRASLDLLRRKSVLLLISDLEPSQEELLMLQQMYSEAREQPGRAESQYEIVWLPVMDRSTPWNETKKKQYEDFQSSMPWYSVYQPSLLDVAVIRYIKEVWHFNKKALLVVLDPQGKVVNPNAIHMMWIWGSLAFPFTSLREEGLWKEETWKIDLLADNIDPALSSWIQQGKFICLYGGEDIEWIRKFTATAKAVAKDARIQLEMLYVGKSNPKEKARKINGVIVNENLSHVLPDLTLIWFFWVRLESMWHSKVQHQRTADNDPIMQEIMTMLSFDGSDQGWAVISKGSDEMAKAKGDTILKSFVDFESWKQSAEVKGFLPALNDHLHELHSPSHCNRLILPGATGSIPERIVCAECGRPMEKFIMYRCCTD

**27042236_peptide|Ptrichocarpa|Potri.001G340200|Potri.001G340200.1**

MAGLPLRHPAQGFNASQQLIKSDRGSMLTMSDDNVMMKQIVGTHAPDGREVDVKPLLLLVEDILKRATLQIDSSLTTSQAHAEMEDKTSHVNFVSMLDALSYTIDRISSEIAYKALGGTDAHATTVSLFNMLTSYSWDAKLVLTLSAFALNYGEFWLLAQIYSSNDLAKSMAILRQLPSIMEHSGPLKPRFDAINNLIKVMMDVARCMVEFKDLPPAYISNEVPALSTAMAHIPTAVYWTMRSVVACAAQITSLTTMGHEFSISTTDAWELSTLAHKLSNILDHLRKQLDTCYQYIDEKRNVESFQMLKNLFEMIHIDNMKVLKALIYAKDDIQPLIDGSSKKRVHLDVLRRKNVLLLISGLDMPTDELSILEQIYNESRHHGPRLDNQYEVVWVPIVDRSVQWSDPMKGKFESMQSSMPWFTVYHPSLIEKAVIRFIKEVWHFRNKPILVVLDPQGKVVCPNALHMMWIWGSNAFPFTSLREESLWKDETWRLELLVDGIDPVILNWINEGKYIFLYGGDDDEWVRKFTNTARAVAQAARIPLEMVYVGKSSKREKIRRVIATITVEKLSYVWQDLTMMWFFWTRLESMLYSKIQLGKLDDHDPMMQEIKKLLSYGREGGWAVLSNGSNVVVNGHKTTVLQTLLEYDLWKEQVPVKGFDMAFQDHHFQLRGIARPCCRFDFPMTTGRIPETMKCPECNSTMEKFSTFLCCHDEVIPDELSK

**27043553_peptide|Ptrichocarpa|Potri.001G340500|Potri.001G340500.1**

MASGLPFRHPAQGFNASQQLIKSDRGSMLTMSDDNVMMKQIVGTHAPDGREVDVKPLLHLVEDILKRATQQIDTSLTTSQAHAELEDKTHQVNFVSMLDALSYTIDRISCEIAYKSLDGTDAHATTVSLFNMLPSYSWDAKLVLTLAAFALNYGEFWLLAQIYSSNQLAKSMAILRQLPSIMEHSGPLKPRFDAINNLIKVMMDVARCVVEFKDLPPAYISNEVPALSTAMAHIPTAVYWTMRSVVACAAQITSLTTMGHEFSISTTVAWELSSLAHKLSNILDHLKTQLATCYQHIDEKRNVESFRMLKNLFEMVHIDNMKILKALIYAKDDIQPLIDGSSKKRVHLDVLRRKNVLLLISGLDMSNDELSILEQIYNESRPHEARLESQYEVVWVPIVDRSVQSDAMKEKFESMQSSMPWYTVYHPSLIEKAVIRFIKEVWHFRNKPILVVLDPQGKVVSPNALHMMWIWGSSAFPFTSLREESLWRDETWRLELLVDGIDPVILNWIKEGKYIFLYGGDDDEWARKFANTARAVAQAARIPLEMVYVGKSSKREKIRRVIATITVEKLSYVWQDLTMIWFFWTRLESMVYSKIQLGRLDDHDPMMQEIKKLLSYDREGGWAVLSNGSNVVANGHRTTVLQTLLEYDLWKAQVPVKGFDLAFRDHQGSIHDISRPCCRFDFPMTTGRIPETMKCPECNRTMEKFSTFLCCHDEVIPDELFK

**27047070_peptide|Ptrichocarpa|Potri.001G340300|Potri.001G340300.1**

MASGLPLRHPAQGFNASQQLIKSDRGSMLTMSDDNVMMNQIVGTHAPDGREVDVKPLLLLVEDILKRATLQIDSSLTTSKAHAEMEDKTYHVNFVSMLDALSYTIDRISSEIAYKALGGTDAHATTVSLFNMLTSYSWDAKLVLTLSAFALNYGEFWLLAQISSSNQLAKSMAILRQLPSIMEHSGPLKPRFDAINNLIKVMMDVARCVVEFKDLPPAYISNEVPALSTAMAHIPTAVYWTMRSVVACAAQITSLTTKGHEFSTSTTDAWELSTLAHKLSNILDHLRKQLDTCYQYIDEKRNVESFQMLKNLFEMIHIDNMKVLKALIYAKDDIQPLIDGSSKKRVHLDVLRRKNVLLLISGLDMSTDELSILEQIYNESRHHGARLDSQYEVVWVPIVDRSVQWSDPMKQKFESMQSSMPWFTVYHPSLIEKAVMRFIKEVWHFRNKPILVVLDPQGKVVCPNALHMMWIWGSSAFPFTSLREESLWKDETWRLELLVDGIDPVILNWIKEGKYIFLYGGDDDEWVRKFTNTARAVAQAARIPLEMVYVGKSSKREKIRRVIATITVEKLSYVWQDLTMIWFFWTRLESMLYSRIQLGKLDDHDPMMQEIKKLLSYDREGGWAVLSNGSNVVVNGHKTTALQTLLEYDLWKEQVPVKGFDLAYRDHQGRIHDISRPCCRFDFPMTMGRIPETMKCPECNRTMEKFSSFLCCHDEVIPDELFK

**27047832_peptide|Ptrichocarpa|Potri.001G340600|Potri.001G340600.1**

MASGLPFRHPAQGFNASQQLIKSDRGSMLTMSDDNVMMKQIVGTHAPDGREVDVKPLLHLVEDILKRATQQIDTSLTTSQAHAELEDKTHQVNFVSMLDALSYTIDRISCEIAYKSLDGTDAHATTVSLFNMLPSYSWDAKLVLTLAAFALNYGEFWLLAQIYSSNQLAKSMAILRQLPSIMEHSGPLKPRFDAINNLIKVMMDVARCVVEFKDLPPAYISNEVPALSTAMAHIPTAVYWTMRSVVACAAQITSLTTMGHEFSISTTVAWELSSLAHKLSNILDHLRKQLATCYQHIDEKRNVESFRMLKNLFEMVHIDNMKILKALIYAKDDIQPLIDGSSKKRVHLDVLRRKNVLLLISGLDMSNDELSILEQIYNESRPHEARLDSQYEVVWVPIVDRSVQSDAMKEKFESMQSSMPWYTVYHPSLIEKAVIRFIKEVWHFRNKPILVVLDPQGKVVSPNALHMMWIWGSSAFPFTSLREESLWRDETWRLELLVDGIDPVILNWIKEGKYIFLYGGDDDEWARKFTNTARAVAQAARIPLEMVYVGKSSKREKIRRVIATITVEKLSYVWQDLTMIWFFWTRLESMLYSKIQLGRLDDHDPMMQEIKKLLSYDREGGWAVLSKGSNVVANGHRTTVLQTLLEYDMWKDQVPVKGFDLAFQDHQGRIHDISRPCCRFDFPMTTGRIPETMKCPECNRTMEKFSTFLCCHDEVIPDELFK

**27050807_peptide|Ptrichocarpa|Potri.012G090400|Potri.012G090400.1**

MASNSMQITPAMNQVIPNELWVSLESNGSTMMKAVRASHAPDGRKVDVKPMLRIIDNILLRAAPAIVEGTYEDKGLLEEEIFSRSDVDAKLKTLASIIKKVSSELFSHNNLILSGTFKANSNVNQTIIPYGMDCKCTCSGGRDVHATIMMLLDTLSSYSWDAKVVLTVAAFIVNYGHEFLLFTSSEAKNSALAQSLAFLKQLRDITDHAKFSKAKIDAITKLIKGIMAVTKYIVELHNLPPESIATDEAPMSNAMAYIPKAAYWTMLSVVICASYIASLSGSEETTELLDLSRLASEGSKMLVILKSYKTIYKEYEDEKCKTAYWDLVKTFSSTHSDNMTVLKTLLLAKDGKQHLVGHTSKRIHIEELREKHVFLLASGLDISKEEIDILGKLYQEAKSKGEVQYEVVWIPVVEKTISWDQEKQQKFEQILAMMPWYAMPEPSKIKAEVIKYIKNEWRFENKMILMPLDRQGKVGSLNALDMLLVRGNKAFSSIYNEKEESIWEAQQTWTLELLLGGIDNNFSLVLDWTKQGTIVCLYGGEDIEWIREFTKATKDAVKNISSGTSFELVYVGKNNAIKEVENIIKTIDDENLSRYWNDLSSIWFFWTRLESMLYLKMQQCKNLKNDRIMQEVTTLLSYDDSDRGWALFCKGSEMALAQGDVALKSMKEFSTERKDADLFQAVSNYIKKSQKIQRCSRIILSGDRQEIPTSFSRALKHRPPSSVLDYENNNVCFVTFFILFL

**27147029_peptide|Pvulgaris|Phvul.009G171300|Phvul.009G171300.1**

MFNKALFHFILLYIYGINTTTNTQAKTYFPPTEFSDICSHFSNMSNHTRQLTATTPKINQRKERRMFSTSDDSAMTKQVQATHAPDGREIDVKPILQIVDEILVRFIARTVEGYEVKRDQDALEMTAVLAEFDMLDSLAYIINKISCELSCKCSGGGDAHSSTMVLLGYMSSYAWHAKVVLTLAAFAVIFGEFWLVAQLSAENTLAKSVALLKQLPDLAENFMSLKPHFEALIRLVKAAMEVTTCIVQFKELPSEYISEDTPPMSVASTHIPIASYWVIRSIVACASQIASLIGTRNESISSTTEAWELSSLAHKVTSIHEHLKNQLILCYQYIDDKRHIEAFHNLTRLFETVHVDNMKILRALIYAKDDILPLVDGTTKSRASLEVLRRKHVLLLISDLDLSQEEILVLDNLYKDARSRGDTHYEMVWIPVVDKAVWNDVNKQKFEYLQSLMAWYSVCDPFIIEPSAVKYIKEVWNFSKTAILVALDPQGRLSSPNAVHMIWIWGNLAFPFTSEKEESLWKQEIWSLELLVDGIDPTVLEWMADGKLVCLYGGEDLEWIEKFTATALSVAKAGKFQLEMVYVGKSNAKERMQKMIKTFNTRKFSYFWPNVTSIWFFWTRLESMLYSKLQHGRTVENDEVMSEVMTVLSFDGSDRGWAIFCNGATEMARAKGDSALVCLQDFDKWKDRVEEEGVVQAMKDYLDKNKPPHHCNRLILPGSTGGIPQKVVCAECGRQMEKYFMYRCCVE

**27155792_peptide|Pvulgaris|Phvul.008G147900|Phvul.008G147900.1**

MNSIGKLGAMQQLINGGRMMPAAAISDDSVLVKKVLTEHNPEGIEYDVRPLLRIVEDVLICSTLTSEGPTTGALARVDNVEDKSYHPGQTNMLEALSAKIDRISCEIPYKTLCGVDVHSTTVAIFDMLTIFKWDVKIVLALAAFALTYGEFWLLAQIHDKNQLAKSMAILKQLPSIMARASSLKPRFDTLNDLVSIIVEVTKRVIEFHDLRSQYITQDISAYTTAYNYIPVASYWTVRSIVACAAQITSLTTLSYEIFASTDAWELSTLIFKLRNINDHLRQLLNSCHEHIGKKMDAEAYQMLRDLFSKPHTDNMKVLKALIYAQDDILPLYDGVTKKRVSLEPLRRKNVLLLFSGMEISTDELMILEQIYNESKTHTSRMESRYELVWIPIVDPNYEWIEPKKKQFESLQENMSWYSVYDPSLIGKPVIWFIQSEWKYKNKPILVVLDPQGRVSCPNAIHMMWIWGSAAYPFTSSREEVLWKEETWRLELLVDGIDQDILNWIKDGKYIFLFGGDDPEWVRRFVKEGRRVAMATQIPLEMVYVGKSNKREQVQKIIDTIIRDKLNTQYWSEQSMIWFFWTRLQSMLFSKLQLKQSDDDDHVMQEIKKLLSYDKQGGWIVLARGSHIVVNGHATTGFQTLVEYDAVWKDHADREGFESAFKNHYDKVHSVVSPCSRFEFSHAMGRIPERLTCPECRRNMHVLTTFQCCHDEKIDEDFFVSTVTPPTN

**27158634_peptide|Pvulgaris|Phvul.007G126400|Phvul.007G126400.1**

MALALLNSSASAITPQQKDKFLNPFELQDSQIRFNVYLTHVNDDKEYDGDVLFNLVSNILNSASAQVSATAVSATSFKPDFPTLKRLSCQMISTRGTPECAHQTTLKILQQLSGFSWDAKAVIALAAFSLEYGEFWRLDRVQIADQFGNSLKQLNQVQISRKVLSDMTDLVTVLGEVLNYINLWAKWSSMDYDTEEVHSLLAAMQEIPLVVYWTIASIVASVGNLVGISEHKLSNFKDRLSKVVIKLKSHMENGKWEIGRIQDFKIRIDIRYPKIKDVVDLLDILIIPASDGGTSIPKIFEDGVQIKNGIEVFKQKYVMVFISGLDSIGDEILLLSSIYRRLQEHAGEEIKGFKKGDFRILWIPMVDDWNTDKIKCEKFYNLKKSMKWHVLEYFEKLPGYEIIVEKLKYDGRPIVTVINPQGQIMNENALQIIFEWEIEAFPFRQIDVDDLNKKWKWFWNLLEKTDDNVKLLGKNNTSCVFIYGGNESWVQNMKNAIGKIEKHEINNVDVNIERYQLGEHNPDHVPSFWIGLDGKKKNKECKGRVDCEIQEIVRTLLCLKQDPSGWVVLSRGRNLKLLGHAEPMYQTVVEFEKWKNTVLEKETFDVAFKEHYDVVKERYASRPYDHTSSVLATITCPNTTCGRVMEVTSINYRCCHGSANSCNF

**27159514_peptide|Pvulgaris|Phvul.007G127000|Phvul.007G127000.1**

MAAKHSLKSLFHRSSESEQNPLNMSDEQILEEIYSTHVHSHTKFDVDSLFILVENTLRRSTLIVDNVVQGSKSSLEQLEDKIPQANFNSPLCTLRQISSEMTCKPAGEEIAHKTTMTILVKLSNYEWDAKAVLTLAAFAMEYGEFWLLAQHQPTDPIAKSVAFLKRVPVLTRPAALQKHRQAIIELNNLVKTTLLVIELIFELEKLTTFDTKDVPALLPAIEQIPVDVYWAIITIVTIETQIDYLTTESGNKQDLSHYGQKINIILSKLRKQIMLCRQQIEEAEYHHRLRKFFQTPTEIMEVFKFLVFSKDAPQLLFDGATKTTVEITELKKKNVFLLISTLDITDEEITVLRPVYEYIKTNDQYKIVWIPIVEEWTEKLHKRFEVLKSKMPWCVVQHFGPIAGYKYIKEEWHFKKKPMVVVLNPQGKVQHANAFHLIHVYGMKAFPFTIADQERIDREIHWIGSVVGDSHPHISTWIREQKYILIYGGSDKEWIQQFTKYATTFANDSTLKEANIQIELFCVEKEDRNFLRRFWSGIESLFVTKANNTVDAVTQEVQRMLSYKNETGWAVLCKGSYVLLSGHGTTILKTVSEFEKWKEGVMKKGFEPSFKEHHERIVRTTHRCVHLEIPNIAGKLPETIKCPECGRTMEIFISYKCCHSDSTSIAIH

**27159767_peptide|Pvulgaris|Phvul.007G126500|Phvul.007G126500.1**

MALSNGTSTTALIPQSGTVSIQHRASLPNPFDLTDDQILDIVYLAHLNDDEICDTDNLYNLVSNIVLRSQSMISASSFKPEFVTLKLISCQMISTRGAAHCVHQTTMWILQHLKCYSWDAKALITIAALSLEYGSFVHLSQFQPNDVLGNSLKQLNQVQSRNVSAVAELVTYTVTVFEQIKEWARYAADGYDPEDVPDLTEAFQAILVVVYWTIAATVASTGNLVGVSSYKLSEYRFRLSAAVDKLTTHLGNCSVQIGNVRDYVIIRNIFDRPKDIVDLLKALIYSQQKGPENPKIFQGSNLVTKGIEVLRLKHVLLFISGLDSVEDEISLLNSMYDRLQEDPKEAKGFKKEDFKILWIPIVEEWSEGSREQFKALKSGIKFYVVEYFYELPGLKIIKDRERLNFESQPIAPLLSPKGTIMNENALDVIFEWGIEAFPFRKTDGEELTLKWKWLWDLILKATPGLQVKENRYIFIYGGSNSAWIQNFTHELSKIKMNESIQRADIILEYYQLGKGKSEPNNSVPSFWIGVERKKQNKKHQEAVDCEIQKIVKCLFCLKRDPQGWAILSKGHNIKHLYHGQAVYQTVAEFQNWKGKVFEREGFDIAFKEYYDAKEKEISAIQPCEAYSSTSSVIGTITCPNPTCGRVMEVSSVNYKCCHRDDALNC

**27160140_peptide|Pvulgaris|Phvul.007G280600|Phvul.007G280600.1**

MSIAKPEQKPQLPNPFHLSDSEIIDKVYASHTYDDEVFDSEPLFNVVSSVIKLSTRVVGAILKIDEQNGFAGNAISISTFKPEFAKLKLMSSQMITTPSGLENAHQTTLRILQQLRNFSWDAKVLTALAGFALEYGNFWNIYQASDPLGNSLKVLNQVQHRQLPLVDLNSTVKLVLEAVEKINRWGTLSADESYDTEDVPALSDALELIPLVVYWVVASLVACNSNIQGVSNYQLLDFRAKLSPALDEFTRQLVICEWQKDRVEEYRRRKRNFNKPKDIVDFLKLLITQNGSQNAQIYDGNARRIVNVEVFKEKHVLLFISGLDRIEDEIRLLNSIYEGLVEDPNERSGFKKEEFKILWIPMESGWGDERREVFNTLKSNIKWYVVEYVEALPGKRLIEEDLKFQGKPILPVVKPQGVLLNDDALDIIFEWGIDAFPFRKTDAYLLAQKWKWFWDEVKKTNLHGIQVKGDRYVFIYGGSDKWSREFSVAVDKIKRHETIKRADAIIEHYHLGKDDPKIVPRFWMGVEGKRQKKHSEKLDCQIQEIVKSLLCLKQDTQGWAILSKGSNVKILGHAQPMYQTVADFEKWKDRVLVKEGFDIAFQEYYETKRHLPAPQPCEFNTLDVLATITCPNASCGRVMEVTSVNYKCCHGGNVADHAFNSPTTQA

**27160894_peptide|Pvulgaris|Phvul.007G126300|Phvul.007G126300.1**

MSLSNGADATSNQQKPQLPNPFDLTDSEILEKVYVTHLHDDDKCDVDVLFDVVSSVVLKTRLAESKAYQTVFKPEFSTMKLISCQMITTPRGDRYVHQTTMCILHHLRSYSWDSKALVTLAAFALEYGNLLHLSDVVTPDNLLTNSLKQLNQIQTRKDPKTILVALVMKVLQDIQEWTRLSSFGYDIVDVPSLSDALQEVPVVVYWIIASLIAATGNLVGVSDYVLSHFVERLSSAASRFKEHLKLSGEQKGYADEYYKRKKAFSNPKDVVELLKLLIGSKVQIYDGSIKSNRDIDVFDQKYVLLFISSLDKIEDEISLLNSIYDRLQENPSEIIKNYKKEDFKILWIPIVTLWDDKQRLKFNALKDTIKWYAVEFFTELPGTDLIKEKFNYRGKPIIPVLTPLADTLNEDAMDLIFQWGIDAFPFRKIDGIDLTLKWKWFWDATKKANLGIQQVTGDRYIFIYGGADKKWVQDFAVAVEKTKGHAIILNTDTIIDQYELGKDDPRVVPRFWIEIERKRLKKHKDAVDCEIQKIVRTLLCLKQDQQGWAILTKGSNVRVLGHGEPMRQTLVEFDAWKEKVFQKEGFDVAFDEYYKIKLNQIYARQQCAIVKNNADVLVTITCPNPTCARVMEVTSVNYKCCHRDAPNTGNI

**27161687_peptide|Pvulgaris|Phvul.007G280500|Phvul.007G280500.1**

MALAASLSPANPLLQTNTNPLGWTDDQILEKVYITHVHTAERHDLESLFNVTSNIIKRATAVADSVAVKTGTPVGLIEDKVALSTFDPPFLKLKHIASQMMNTPHGEDKAHDTAMLILNQLRSYSWDGKAILVLAALALEYGTFWHLVQVLTGDNLGRSLAQMHRVHIVERNRQAVADYNSLVKNLLMAVECISELERLSTKGYDKKDVPALDEAMQEIPVAVYWAIVTTVVCANHFDFFLGESDDRYEIANFDDKLSAVISKLKANLTRSRKRIGDLEDYWRRKKLLQTPTEIVEVIKVLIYHNEVHEPHVYDGLTRQMVSIEAFRKKHVLLFISGLDSIRDEIRLLQSIYEGLQEDPKEVKGYRKEDFRILWVPVVEDWNLLHKAEYDNLKLEMPWYVVEYFYPLAGIRLIREDLNYKNKPIIPVLNPQGRVVNYNAMHMIFVWGIDAFPFRPSDDDVLTQKWNWFWAEMKKVNPKLRDLIKADSFIFIYGGSDRKWMQEFTQAVEKIKRHEIIKRADAVIEHYAFGREDLGIVPRFWIGIESLFANMIQKTHKDPTIDEIKSLLCLKQQQPGWVLLSKGSNVKLLGSGEPMLSTAGDFEIWKEKVLEKAGFDVAFKEFYDQKRRSYPQECSHMQLANYPADILHPINCPDAGCGRSMEIASVSYKCCHGHTHNQAEVPESGNVMIEKKYAS

**27164961_peptide|Pvulgaris|Phvul.006G008400|Phvul.006G008400.1**

MSESGSNSSNVQQGMQKQKQNPFEFSHDKILDDVYRTHFHCLEKCDVASLHTVASNVLNQSMDITHKVIAKGEQLMDRFREDTTTITSQQLAAKLKRIAYLMICTPRGEYPVHCTTMLILEQLKHYSWDAKVLIVEAAFALEYGKFLYLLPLIAPCQQFESSFADLHGLLMVPQNTKQLIDFNSVVKKVMKVVECITQWKKLNSEGHDLRDVPTLPETLQEIPVLVYWTIFTFVTCTGEINHFIDNKYQRHELWTDLENKLDTILGKLNELLKKCINEIERIEDFTRRKNIVTHGRDIVKVLKALIICGDNRDLRQSVYYVGHTEEQVKVEEFKKKYVLLFISGLENIKEDIKVLNSINEKLKESREVENHRSEDFKILWIPIVDEWNEERRKKLEINLRDTKFGWYVVKYFNCETSLKLIKEVFNYEGKHIIPLINPQGKVENIDAKQIISQWGIDGFPFRTSDQSRLSQQWKWFWSEITKFNPAIEHLSEEEDCFLFIYGGSNTKWVEEFTTAMESLRKEVENTNLDSNIVIESYQLDSKVVTRFWIAIDSLLTRRKQVKGGERVKEFATNETIKRLLFLKQDPKGWGILSKGKNVRLLGHGEAMCRTVKEFETWRDKLHELSFDVAFTDHYKGIKDKDGSKKCEENVICYGHPTDIVERITCPKKDCRRPMKVTSVNYKCCHQQGQ

**27165237_peptide|Pvulgaris|Phvul.006G008500|Phvul.006G008500.1**

MMSKSCPSSNMQQGKTLLHNPFEFSDEQILDNVYRTHFHCIEKCDVKSLHAAASTVINHSIQITDTVIEQASQLSERFREETTTSRELTAKLRRIACQMVCTSRGDHYAHHTTMLILEQLKAYSWDAKALIVEAAFALEYGKLLYHPLTKQYQHSEKSLADLNGLLVIQQNTQHLTFFNSVVKKVMQVIECITEWKRLTSAGYDIKDVPTLAETLHEIPVVVYWAIFTFVTCTGQIDDFTTDHKVHRHELSKNFENKLDLILRTFREHLEMCSTEIGRIDDYSRRKNIINITGKDIVKVLKALIISSENRDSRQNVINGLTLEQVKIEEFKKKHVLLFISGLEHIDEESQLLKSIYEKLKEKPREVEGYRKEDFRILWIPIVDEWNEERRKTLETKLQRTKFGWYVVKHFNFETGMKLIKEVFNYREKPVIPLISPEGKVENIDTKQIISMWGIDGFPFRTSDHTRLTQQWNWFWSEMTKLNPRIGDLIEEDRYLFIYGGTDSMWIQEFTIAIEKLKRDVETLSLQIDIAVDSYQLGREDPKIVPRFWIAIDSLLGSRKQQIMKGGDQGVQDFATREIKRLLFLKQDPKGWVILSKGYNVKLLGHGEAMSLTVKDLSLWHVKLHEEVSFDVAFKDYYESIKLKTSPKKCEHSEISNYPSDILAHIPCPNMECGRSMEVSSVNYRCCHGLDP

**27165442_peptide|Pvulgaris|Phvul.006G021400|Phvul.006G021400.1**

MSSKQVSSNTVHQEKLLLNPFEVNDDQMLEKIYMTHFHCVEKYDVGSLYSIASNVINHSIEIADLIKENGQQIEQVREEKDPLNTFPRLPTLKRISCQMICTARGEQYAHQTTMLILKQLRDYSWDAKAVIALAAFALEYGKFWQLAPIHRDKLGKSLAELNGLRSLMENLQQLANFNNLVKKVMQVVKCITDWKKLITGEYNIKDVPSITDTLHEIPVLAYWTISTLVTCTSHIDFLGDKGYRYDLSKFDYKLDYILKNFKEHQEKCNTQIGRIEDYSKRKDIIYNSTESDIVKHLEALIIPTDPQDPRPVVYNVLTAKEVGIGVFKNKHVLLFISGIDNIEYETQLLISIDGKLKEEPREVEGYMKDDFSILWIPVVSVWDEEQRKKVENITEVGWYVVKEFNFQTGIDLIKEVFNYKGNPIILLINPRGKVENFDAKQIISMWGIEGFPFRTSDLIRLTLQWNWFWTEMKSLSPTIRELSNRDCYIFIYGGSNTKWVEDFTTVVEKLKNNGTFNLVETTIESYKLGSESPKIVPRFWITIDNLLASRKRIKKGGEEVKDSTTREIQKLLFLKQDPNGWAILTKGSHVKLLGHGEAMLITVRDFEGWKAKLDHEVSFDVAFKDYYEKCKVKTVPKKCEHREFANYPTDILAHLPCPNKCGHEMEVASVKYQCCHGHETSDIA

**27167398_peptide|Pvulgaris|Phvul.006G021500|Phvul.006G021500.1**

MSSKQVSSNTVHQEKLLLNPFEVNDDQMLEKIYMTHFHCVEKYDVGSLYSIASNVINHSIEIADLIKENGQQIEQVREEKDPLNSFPRLPTLKRISCQMICTARGEQYAHQTTMLILEQLRDYSWDAKAVIALAAFALEYGKFWQLAPIHRDKLGKSLAELNGLHSLMENLQQLANFNNLVKKVMQVVKCITDWKKLITGEYNIKDVPSITDTLHEIPVLAYWTISTLVTCTSHIDFLGDKGYRYDLSKFDYKLDYILKNFTEHQEKCNTQIGRIEDYSKRKDIINNSTECDIVKYLEALIIPTDPQDPRPIVYNVLTAKEVGVGVFKNKHVLLFISGIDNIEYETQLLISIDGKLKEEPREVEGYMKDDFSILWIPIVSVWDEEQRKKVENITEVGWYVVKEFNFQTGIDLIKEVFNYKDNPIILLISPQGKVENPDAKQIISMWGIEGFPFRTSDHTRLTQQWNWFWTEMKSLSPTIRELSNRDCYIFIYGGSNTKWIEDFTAAVEKLKNNGTFNLVETTIESYKLGSESPKIVPRFWITIDNLLASRKRIKKGGEEVKDSTTREIQKLLFLKQDPNGWAILTKGSHVKLLGHGEAMLRTVIDFEGWKEKLDHEVSFDVAFKDYYEKCKVGIIE

**27169636_peptide|Pvulgaris|Phvul.002G258300|Phvul.002G258300.1**

MAMVPRKMQSRATRNIFSASDDTTMTKNVRATHAPDDRHVEVRPLLNVVQDILHRVASLIPDIVQGKPVQTGAVEDSNHQSDLAEVLDISYYTINQISCEICCKCSSGDGHATTMGILSMLSGYSWDAKVVIALAAFAANFGEFWLVAQLYATNRLAKSVAKLKHIHETLEQVDDLGLKFETINNLFKAMLDVTNYIVQFHELPSRYIHPEAPEMLTASILFPGAVYWTIRSILFCASHLLDITGLAHGYMTTITESWELSSLAHKLDNLNGHLRKQITLCHLHLDDNKQREAFETLQLLFETSHQDNLKALKAMFCSNNDPLPLFDGSSKQRVSIEVLRRKIVLLYISDLQNVSDQELVIFEQMYIESRQDSTRLESQYELVWIPVVDKGIPWTELKPKFDMVQSTMSWYSVYDPTALEPATIRYIKEVWLFKAKPILVVLDPLGKVVNLNALPMMWIWGSLAYPFSISKEEALWNNEKWGLVLLADSIDPSLLYWISEGKYICLYGGDDMDWIRKFTRTAYALAAALQLPLEMIYVGKSNPGKKVQETINAIQAEKLSNVLPDLAIIWFFWVRLESMWHSKSQHSKTVENDNIMHEVMRILTYDSGDSGWAVISEGTGSMAQGKGDAFLRCLNEREQWKDTAKDIGILPAMADYIQGLQAPHHCNRLILPGSSGGVPDSVVCAECGKTMEKFYMYRCCNE

**27251919_peptide|Fvesca|gene10000-v1.0-hybrid|mrna10000.1-v1.0-hybrid**

MASTPLKLGPKMRRENRGSLQSDDTALVRQIRETHVPGRSHIVDVKPILHVTDEIFHRSSVVTDGGILLGAHVDSLEDRTSSLGYDVLLQGLSYLIQKIYIEIGCQSCADVHASTLELLRSLSNYSWEAKVVLTLAAFSIYYGEFWLVAQLCATNSLAKPVAILKQLSDLIEHAATVKPQLEAIDNLIKAITKVTKRIVECADMVKLQSQYISEDQPPLMTAMAHIPAAAYWAIRGILACASHISVLTGGRYEYVASTTEVWELSSLAHKLNNIDEHLNSELENCRRHIVEKRYDEDYKNLIHLFQSLHLDNMKNLRALISHKDDVQPLVIGRSQVRCSLEVLRRKHVLLLITDLSLSYEEVLILEHIHKDQQSRGEIEYEFVWLPIVDAALWNEAKKERFEDLKSRMPWYAVHDPLIIEPPVIKFIRDYWHFDKKMIIVSLDPQGRVSSANAFHMLWIWGNLAFPFSDEKENALWNAESWRLELVTDGIDPEILKWIDQGKYICLFGSEDIEWIRRFTVRAKDVAKRAGIPLELVYAGRSTASKEKLWKLNKIIEAENLSRTWSDYTSTWFFWSRLDSMRCSKAKHHKTIDDDTILREVMSLLSYDGSDHGWVMLWLGSADTVRANGHLTTSTLDDFEAWKPAAAELGFLPELKNQLKQRHEPHHCTRLIIPGFGPDIPDKVVCTECGREMEKFFMFRCCTD

**27276697_peptide|Slycopersicum|Solyc04g026020.2|Solyc04g026020.2.1**

MTSVNPLNEKVSMTTNSTNNTSSGSSDVAANRMHPMSNHINPLNVQINPHSVVKPASHDTIPATAVPAAHHTTPINPQTSNLAARLPHRRGDHRMFLASDDNTMMKHIEETHIPDGRDFDVKPLVHIIEDIVHRATPIAGHVHEAKVQAHLQALDEKAPHSGLTEILYYLAYPIQRIKVELISKCAKKEDAHSTTMSLLHSLTTYAWDTKVAITFAAFAQLYGEFGLLVHQYTTNPLAKSVAIIMELPEIMTHQDVLKQKFDAIHDLIDKMLDVTKCIIEFRDVQSSHSQHVITQELEMLINTAHISTAAYWTMRAAVMCAAMILNLIAIGHEQISSTSEAWEISNLAHKLANILDHLRKVLNLCHQKIEEKRQYDAFEAILRLLRTPQLDNMKILSMLIHSRDDQLPLFDGTHKRRVSLDVLRRKHVLLLISDLDIASEELFVLHHMYDESKTQPNRPESNYDVVWMPVVDKRLTPWTEAKQMKFEEVQASMPWYSVAHPSMMDPAVLRCIKEVWGFKKKPQLVVLDPQGKESNNNAYHILWIWGSLAFPFTKARETALWKEQTWNIELLADSIDQNVFTWISEGKCICLYGGEDIEWIRSFTSATRAVANAARVPLEMLYVGKKNPKERIRKNNSIIQIENLSHVVQDQTLIWFFWERLESMWHSRTQQDIPGETDPILQEIVTILSYDGSDLGWAVFSRGLVEMTRGKGDLIVQVMKGFDRWRNEVSDITTFVPALDRQLRDLHSPHHCTRLILPSTTGHVPERVICAECSRPMEKFIMYRCCTD

**27291328_peptide|Slycopersicum|Solyc03g111810.2|Solyc03g111810.2.1**

MASHALVPAAATTRGMKPTQQAMTKRERPVFSLSDDHAMSKKILDTHNPDGREVDVNVILHIVEDVFQHSYPAAMDGVLNGAGAHHLEGNIEALKLEEKASLAFDGILEGLAYIIHKVSCELTCKCSSGGHDAHSTTMSVLGMLSGYQWDAKLVISLAAFAVTYGEFWLVAQMFATHPLAKSVAILKQLPDIMEHSASLRSRFDAINELIKAILEVTKIIMEFKKLPSQYITEDQPPLSVAISHIPTAVYWTIKSIVACASQLTTLLGMSYDMIVATTADTWEMSSSTHKLKNISEHLRVELNRCYAHIQEKMHVEYFQMLVHLFEATQFDNMKIMRAMIYIKDDLLPLEVGTTHTRASVEVLRRKTVLLLLSDLDASHEEILVLSQIYLESRSRPEFQYEIVWLPIVDRSKGWNDAQEQKFKELQALMPWYTLHHPSLLEPAIVKFVKEKWHFTKKMMLVTLDPQQGKVACPNAIHMAWIWGNLAYPFTLSKEESLWNMESWRLELVVDGIDPNLIDWMASGKFICLYGGEDMDWIRNFTKSARSVAQRAGIDLQMLYVGKSNNKERVRRINSMITAENLSYCLMDLTSVWYFWTRIESMFYSKMQLGKTIQEDKVMQEVLTMLSFDGSDQGWALISRGSFEMARAKSQIITKTLDDYTVWEEDARVKGFVPALIEYFLQLHTPEHCNRLILPGLDGDIPEMIVCAECGRPMERFFMYRCCTD

**27292046_peptide|Slycopersicum|Solyc03g111820.2|Solyc03g111820.2.1**

MASNALVSAATTRGVKPTQHAMVKRERPIFSLSDDHAMFKKILDTHDPDGREVDVDVILHIVEDVFQHSYPAAMDGVLNGTGTDHLEANVGALKLEEKTSLAFDGILEGLAYIIHKVSCELTCNCSSGGHDTHSTTMSVLEMLSNYHWDAKLVICLAAFAVTYGEFWLVAQLFGTHPLAKSVAILKQLPDIMEHHGSLKSRFDAINELIKAILDVTKSIMEFKKLPFQYITEDQPPLSVAITHVPTAVYWTIKSIVACAFQLTTLSGMSYDMIVATTADTWEMSSSTHKLKNISKHLGAELNRCYQHIQEKMHIEYFQMLVHLFEATQFDNMKIMRAMIYIKDDLLPLEVGTTQTRAIVEVLRRKTVLLLLSDLEASHEEILVLSQIYLESRSRPEIQYEIVWLPIVDRSKEWNEEQEQKFKELQALMPWYTLHHPSLLEPAIAKFVKEKWHFSKKMMLVTLDPQQGKVACPNAIHMTWIWGNLAYPFTISKEESLWSMESWRLELVVDGIDPNLMDWMASGKFICLYGGEDMDWIRSFTKSAQSVAKRAGIELQMLYVGKSNNKERVRKINSTITDEKLSNCLTDLTSVWYFWTRIESMFYSKMQLGKTIQEDKIMQEVLTMLSFDGSDQGWALISRGSFEMARAKSQIITKTLEDYTTWEDSAKTKGFVPALIEYFLQLHTPEHCNRLILPGLDGDIPDVIVCAECGRPMERFFMYRCCTD

**27297865_peptide|Slycopersicum|Solyc05g013850.2|Solyc05g013850.2.1**

MTSINPLNEKVSMNNPMTTNPMNNMSSGSAANMMHPISNPTTGHHINPLNAQINPHSVVKPVSHHDMIPASVVPAAHHTAPINPRTSNLAARLPHRRGDHHMFLTSDDNAMMKHIEETHIPDGRDFDVKPLVHIIEDIVHRATPIAGHVHEAKVQAHLEALEEKAPHSGLTDILNYLAYPIHRISMELISKCANKEDAHSTTMSLLHSLTTYAWDTKVVITFAAFAQQYGQFGLLAHQYTTNPLAKSVAIIMELPEIMSRQDVLKHKFDAIHDLIDKMLDVTKCIIEFRDVQTSHSQHVITQELEMLINTAHISTAAYWTMRAAVMCAAMILNLIAIGHEQISSTSESWELSSLTHKLANILDHLKKVLNLCHQKIEEKRQHDAFEALLRLLRTPHIDNMKILSILIHSRDDQLPLFDGTHKRRVSLDVLRRKHVLLLISDLDIAPEELFILHHMYAESKMQPNRPESNYEVVWIPVVDKRLTPWTEAKQMKFEEGQASMPWYSVAHPSMIDPAVIRCIKEVWGFKKKPQLVVLDPQGKESNNNAYHMLWIWGSLAFPFTKARETALWKEQSWNIELLADSIDQNIFTWISEGKCICLYGGEDIEWIRSFTSSTRAVANAARVPLEMLYVGKKNPKERVRKNSSIIQMENLSHVVQDQTLIWFFWERLESMWHSRTQQDIPGESDPILQEIVTILSYDGSDLGWAVFSRGLAEMTRGKGDLIVQVMKGFDRWRNEVSDITTFVPALDRQLRDLHSPHHCTRLILPSTSGHIPERVVCAECGRPMDKFIMYRCCIE

**27298928_peptide|Slycopersicum|Solyc05g013870.2|Solyc05g013870.2.1**

MTSVNPLNEKVSMNNPMNNMPNGSAANMMQPMSNPTTGHHINPLNVQINPHSVVNPVSRDMIPASVVPAAHHTAPINPRTSNLAARLPHRRGDHHMFLTSDDNAMMKHIEETHIPDGRDFDVKPLVHIIEDIVHRATPIAGHVHEAKVQAHLEALEEKAPHNELIEILNYLAYPIQRIKMELISKCANKEDAHSTTMSLLHSLTTYAWDTKVAITFAAFAQLYGEFGLLTHQYTTNPLAKSVAIIMELPEIMTRQDILKQKFDAIHDLIDKMLDVTKCIIEFRDVQSSQNQHVITQELEMLINTAHISTAAYWTMRAAVMCAAMILNLIAIGHEQISSTSEAWEISSLAHKLANILDHLKKVLNLCHQKIEEKRQYDKFEAILRLLRTPQLDNMKILSMLIHSRDDQLPLFDGTHKRRVSLDVLRRKHVLLLISDLDIAPEELFVLHHMYEESKAQPNRPESNYEVVWIPVVDKRLTPWTDAKQVKFEEVQASMPWYTVAHPSMIDPAVLRCIKEVWGFKKKPQLVVLDPQGKESNNNAYHILWIWGSLAFPFTKARETALWKEQTWNVELLADSIDQNVFTWISEGKCICLYGGEDIEWIRSFTSSTRAVANAAGVPLEMLYVGKKNPKERVRKNSSIIQMENLSHVVQDQTLIWFFWERLESMWHSRTQQDIPGETDPILQEIVTILSYDGSDLGWAVFSRGLAEMTRGKGDLIVQVMKGFDRWRNEVSDITTFVPALDRQLRDLHSPHHCTRLILPSTSGHIPERVVCAECSRPMEKFIMYRCCTD

**27421011_peptide|Tcacao|Thecc1EG025178|Thecc1EG025178t1**

METPSAFLSSKTSVQLLDEIRATHANDDQRDVDVKPILLSMTKILDDVEGDIKGTARGRRVTSKGSTSLPPAFDHSMLESILADIRNVSGEFSCNCSEGENAHATTMKLLETLKTYSWNTKVVLALAAFTANLGESWLLLQRGNTNSLATSVALLRQVPEIDRLDLLGSEVGKLIQAIRNLASCNAKFMMKVHPWYFSKDTSPISEAKLEIIRAAYWTIHSVVQIASLIGRRNKSTALSMEKGKTLVAHLETEVSEISKILMYHLKRCKEYIGKEMEDAYQSLLELMQRSGRDIVEILNRFLCHGNMDKVDIEKLRSKHVLFLISDLDISLGEITVLNELYLKGEGYEVVWLPVVDGLYDKKKFVELKSSMKWYTDVPAILDPAVIKYIKEVWHFIKNQIAVVLTPEGKVTCQSALPMLWTWGNEAVPFTAEKIVERKLICLYGGGSIDWIREFNTGLKFVLARIGESVEMVYVGKNNDKDWTEKVIRDLRVIKSERHFWARLQSMLYLYTKMRQGKTLTKDDPFMQEVMKILSYDGGDRGWALFCKGPDVKVRMDGETARAIISKHGDLETYAKRHGFLEGLNYYMEEQDIPHSCVLQLPLIDSEVPGKMCCDQCGREMEMHYTYRCRAL

**27425217_peptide|Tcacao|Thecc1EG025263|Thecc1EG025263t1**

MAATTSNSQSSLHSILQTHAFDQTREVDLGPLVKVIESILQNAAAASVSKGAYEGLSDMIGSLENFDGMRQVLDDIRSISCEMSCNLSDVEATTMELLKRLRNYSWNAKVVLAVAAFASSIGELLMLVNHRTTDPIAKSVDLLKGHSFKLDINVLKQLLKAMMDVVNVNFALLVPTLSKIPKEAPSMKDAMEYFPTATYKILSVIVQIASILSKREQIIDSTIKSLAYEVSCVNYVLQKKLERCKIDAEQGEKYHKHDETEYQRDEDVSELIPKIGFWELFGKIKQHMKIQVPEKLRKKHVLFLISDLDISFEEIKVLDRLYQKNNQRYEILWLPIADLTTPYEKTKFSELKQLMKWGAVEPSKIGPAFIEYIKKEWHFIKNPIAVSLNEEGEVTCRNALPMLWTWGNLAFPFTDEKEHHFWNKIDEKNGWKLELLLDEQIDLDIPPRMRSVTFVCLFGGENISWIQAFTEKVKNATAGVSFKLVYVGKNKREGLPSHLLSPDIHVIDSEFQWQFWTRLETVMVTANGEMALTIMSNYKDWKKDTTGPLFLEALRNYCTYKIPDIHGCINVHLPVVGEIPGIVSCPTCSKEMEMYYTYRCCSE

**27448261_peptide|Tcacao|Thecc1EG014515|Thecc1EG014515t1**

MATPNVHPSKSQQLLRRDRRIFSVSDDNSMMKQIQSTHAPDDRVVDVKPILQIIENVLRHVTPNIDRALNAADQGHIDGFDDRASLAAIDGMLEALAFIVHKISCEISCKCSGGGDAHSTTMALLNMLSSYSWDAKVVLTLAAFSVNIGEFWLIVQLCTTNSLAKSVALLKQLPDILEHSHNLKPQFDALNNLIKAMMDVTKCIVEFTELPSQYILSDVPPMSIAMAHIPTAAYWTMRSVVACASQIASLVGLKHEHITATSEAWELSSLAHKVSSIHDHLQKLLRQCYQHIDEKKQAEAYEGLAHSFGTPQLDNLKILIKLFSLGKEDPQNALLGPDKTKVHMDVLRRKHVLLLISDLDISPDEIQVFEVLYKYERVSSELNYEIVWLPIVDMSTWNDGHQQKFLNLQSIMPWYTVHHPSIIEPAVIKYTKEVWRFVKKPIVVTLDPQGKVTCPNALNMLWIWGNTAFPFNTETEESLWKTQAWTIELLVDGLEANLHTWMKQQKVICLYGGEELDWIESFTSETKKVAQALGIGLEMVYVGKNNARERVRKITGFINEKQLSHAWQDGTIWFFWNRLESMLFSKTRHGKTNETDVIKQEVMTLLGYDGSEHGWAVFFLGSIEMVRAKGDKALSSMQSFETWEYLAREMGFMPALRKYLEGIAEDHHCTRLILPGISGGIAERVVCAECGRPMEMYFMYRCCVD

**27452889_peptide|Tcacao|Thecc1EG017019|Thecc1EG017019t1**

MESKLTPAAKMQQQPIVGEKLKFSASEDGVMLKQIQASHAPDGRVINVRPLLRIVEDIFNRAAPSAIVAPAHTEEVEDQTYQADVIDMIEALSFLIDRISSEIAFKCTETGEAHATTMSILNIVSNYPWDAKLVIALSAFAVNYGEFWLLAQSYTSNQLAKNLAILRQVPEILQHSSMLKYRFETTKNLIRAMVDIAKCIVDFKELPSKYISADVTAMSTAMDHIPIAIYWTIRSMLASASQITGLSGFGNEYLLSTMESWELSSLVHKLDSMHSHLVGLLAACHKHIDERKFLEAYQNLLYLYETAQIDNIKILKALINPKDDPLPLIHGATNRRVNIDVLRKRNVLLLISDLDILQDKIAILEQIYNESRSQPSRLESQYEFVWLPVLDPSVPLSEIKKDKFENLKALMTWYTLQHPSLIDRAVFKFIKEVWHFEKKPILVVLDPQGRVTCPNALYMMWIWGSLAFPFTTGRETALWRAETWRLELLVGGIDPVILNWISEGRFIFLYGGEDMDWIRKFTNAVRTFARASGFSLEMVYVGKSNPKERVSRNMATITAEKLSFCLPNLTAVWYFWIRMESMWYSKYQLGKEDENDPITREIMTLLTYDGSGDGWALLSRGSAELTRAKGSAFLTCLTEYNLWAADLQTKGLVPAVHDYFLQHPAPHHCNRLELPGTTGRIPERVTCSDCGRMMERYILYRCCDE

**27453462_peptide|Tcacao|Thecc1EG017020|Thecc1EG017020t1**

MAQKTSPGALQAPMNAPLNNPAAAAQALLHNPGAAHAFLNNPGAATQALLTTAGATQPLLSNPGVGQQITSHPGAIQPHISTPGTNQPFISKPSAAQALMKSPSASHALIRSDRGSMLSMSDDNVMMKQIMTTHAPDGREVDVRPLLYLVEDILNRATQHVDFLVKGTLAQIELEEKAQQANYIAMLEALTYIIDRIACELSYKAMGGSDAHATTTAIFNLLSSYAWDAKLVLSLSAFALNYGEFWLLAQIYSTNQLAKSMAILKQLPSILEHTAPLKPRFDALNNLIRTMMDVTRCVVEFKELPSMYISQEVPALATAMTHIPTAVYWTIRSMVACATQISSLTSMGHEFAISTSESWELSSLAHKLKNIYEHLKQQLSLCYKYIDERKDVETYQMLLNLFDPSVMHIDNMRILKALIYARDDKLPLLDGSTKRHVSLDVLRRKNVLLLISSLDFSSDELAILEQIYNESRVHATRLESQYEVVWIPVVDHSVVPLTGEIQTKFENLRSTMPWYSVQDPKFIEKPVIRFIKEVWHFRNKPILVVLDAQGKVVCPNAIHMMWIWGSNAFPFTSLREEALWREETWRLELLVDGIDPIILNWIKEDKYIFLFGGDDVEWVRKFATAARSVATAARIPLEMVYVGKSSKREQVRKVTAIINAEKLSYAWQDQAMVWFFWTRLESMLFSKIQLGRADDHDPMMQQIKKLLSYDREGGWAVFSRGSNTMVNGHSSTVLPALGGYDEWKVKVPEIGFESAFKEYHDRLHDVAHPCCRFEFPTTTRIPENMRCPECRRFMEKYTTFLCCHDEQGIPGSLF

**27463651_peptide|Tcacao|Thecc1EG010641|Thecc1EG010641t1**

MELSAMSHGRSDPHMFSSASGGKAIGKQIEAIHDPAGIHTSMKPVLDIVEDIFRRAAPPGRGTVQEAHMQVDASDERALHSSADELIDYLSAIINRISCEIAYRLSIGEDAHATTLAVAHVVRSYSWDAKVVLALAAFAMSYGEFLLIVQLYTTNPLAKGVALLKQLPEVLARADLLKTKFDTLANLINAMHCVAKCVIEFKELPSQYISPEDPELSSANSDIPSAVYWTIRSTVVCASQIIGLIGMGHEFVSSTTDAWELSSLAHNIDSICSDLMEKLKRCRQRINERKDIEAYQTLLRLFDAVHIDNMKILKALIYAKDDQLPLWDGTTKQKVSIDLLRRRTVLLFISDLEIPHDDILILEQMYNESQAHPTRVESQYEVIWIPVVDRSVPFDDTKREQFESLKAMMPWYSVSHPSMIQPAVIRCIKEVWDFSKKPLVVVLDPQGRVVNSNAIHMMFIWGNSAFPFTKIREEALWKEETWRIELLADSIDPSIINWLTEGKFICLYGGEDMDWIRKFTTTAKAVAQTANIKLEMLYVGKSNPTEKVRRNMTTIQRENLSRVLSDISLMRFFWVRLESMWHSRVQHGVTVENDHILPEIMTMLSFDGSEQGWAVISRGSDELARAKAEIVLKSLDQYPVWEALAAEKGFIPALNDHIRGLRTEHHCNRLILPGTAGIRSIHERVVCFDCGKQMEKFFMYRCCTD

**SPECIES LIST**

**“BASAL” ANGIOSPERMS:**

**Amborellaceae:**

*Amborella trichopoda*

**MONOCOTS:**

**Araceae:**

*Spirodela polyrhiza*

**Musaceae:**

*Musa acuminata*

**EUDICOTS:**

**Brassicaceae:**

*Arabidopisis thaliana*

*Arabidopsis lyrata*

*Brassica rapa*

*Eutrema salsugineum*

**Caricaceae:**

*Carica papaya*

**Cucurbitaceae:**

*Cucumis sativus*

**Euphorbiaceae:**

*Manihot esculenta*

*Ricinus communis*

**Fabaceae:**

*Glycine max*

*Medicago truncatula*

*Phaseolus vulgaris*

**Linaceae:**

*Linum usitatissimum*

**Malvaceae:**

*Theobroma cacao*

*Gossypium raimondii*

**Myrtaceae:**

*Eucalyptus grandis*

**Phrymaceae:**

*Mimulus guttatus*

**Ranunculaceae:**

*Aquilegia coerulea*

**Rosaceae:**

*Fragaria vesca*

*Prunus persica*

*Malus domestica*

**Rutaceae:**

*Citrus clementina*

*Citrus sinensis*

**Salicaceae:**

*Populus trichocharpa*

**Solanaceae:**

*Solanum lycopersicum*

*Solanum tuberosum*

**Vitaceae:**

*Vitis vinifera*
